# COVID, crisis, and *un*ordinary order: A critical analysis of Australia’s JobKeeper wage subsidy scheme as an exceptional measure

**DOI:** 10.1007/s41020-022-00166-9

**Published:** 2022-06-02

**Authors:** Vincent Goding

**Affiliations:** University of the Sunshine Coast, Sippy Downs, Australia

**Keywords:** Carl Schmitt, Order, Exception, Crisis, JobKeeper

## Abstract

Carl Schmitt’s famous articulation of the relation between sovereignty and the exception emphasises not simply the basis for a suspension of the law in a state of emergency, but the role of the sovereign in deciding upon the existence of the ‘normal situation’, the ‘everyday frame of life’ which the law requires to function. Our pandemic times have included extreme biopolitical measures deployed to manage the health crisis, but also unprecedented political responses to regularise or stabilise the economic order. One example is Australia’s historic JobKeeper wage subsidy scheme. As law, it was given life by an executive power predicated on nationhood and enlivened by crisis. As policy, it was intended to help businesses retain workers through targeted, proportionate support. In reality, it also provided significant protections and even windfalls to corporations and their investors, leading to critiques of the scheme as corporate welfare. However, rather than highlighting deficiencies of the JobKeeper programme, these outcomes underscore its ultimate function. This article analyses the relationship between norm, exception, and order in the context of Australia’s flagship economic-policy response to the pandemic. First, by analysing the mutually constitutive relationship between norm and exception, employing the theories of Carl Schmitt and Giorgio Agamben. Second, by critically examining the legislative basis for JobKeeper, its political narrative and practical outcomes. Third, by demonstrating that the scheme, though an extraordinary departure from policy, can be understood as fundamentally a different and exceptional method to secure and reproduce our neoliberal corporate order in a state of exception.

**Supplementary Information:**

The online version contains supplementary material available at 10.1007/s41020-022-00166-9.

## Introduction

The COVID-19 pandemic has seen extreme biopolitical measures deployed to manage the health crisis, but also unprecedented political responses to regularise or stabilise the economic order. One example is Australia’s historic JobKeeper wage subsidy scheme. As law, it was given life by an executive power predicated on nationhood and enlivened by crisis. As policy, it was intended to help businesses retain workers through targeted, proportionate support. In reality, it also provided significant protections and even windfalls to corporations and their investors, leading to critiques of the scheme as corporate welfare. However, rather than just highlighting deficiencies of the JobKeeper programme, these outcomes underscore its ultimate function, namely to preserve and realise our normal political and economic order, in which such outcomes represent not exceptionality, but normality.

This article seeks to contribute to the growing body of literature analysing the exceptional legal measures deployed in the context of the pandemic and, in particular, the literature engaging the work of Carl Schmitt for that purpose. The article analyses the relationship between norm, exception, and order in the context of Australia’s flagship economic-policy response to the pandemic in three ways. First, by analysing the mutually constitutive relationship between norm and exception employing the theories of Schmitt and Giorgio Agamben. Second, by critically examining the legislative basis for JobKeeper, its political narrative and practical outcomes. Third, by demonstrating that the scheme, though an extraordinary departure from policy, can be understood as fundamentally a different and exceptional method to secure and reproduce our neoliberal corporate order in a state of exception.

The article begins with an outline of its theoretical framework in terms of a consideration of the jurisprudence of Carl Schmitt and the state of exception (section 2). It then proceeds to analyse the nature of Australia’s JobKeeper scheme (section 3) and work through both the legal (section 4) and political (section 5) justifications for it. The critiques of the scheme, particularly as it applied to large publicly listed companies in Australia, are then considered (section 6) before situating these critiques in a broader consideration of the nature of Australia’s neoliberal corporate order, asking whether the outcomes of the scheme are exceptional or a continuation of a more fundamental underlying norm (section 7).

## Carl Schmitt, exceptional legality, and the COVID-19 pandemic

In the context of the historic global disruption that is the COVID-19 pandemic and given the wide use of exceptional legal measures as part of the health and economic responses to the crisis from governments of all persuasions, the infamous German jurist Carl Schmitt is an obvious reference point. Key to Schmitt’s work is his understanding of sovereignty as inherently intertwined with exceptionality. Schmitt defined the sovereign as ‘he who decides on the exception’.^1^ Schmitt’s definition is distinguishable because, rather than focussing on supreme power, what matters is ‘*who* decides’.^2^ Though while Schmitt unequivocally associated sovereignty with the ‘borderline case’—‘it is precisely the exception that makes relevant the subject of sovereignty’—he was less clear on what *constitutes* the exception, describing it only as some ‘extreme peril, a danger to the existence of the state, or the like’.^3^ On its consequences, however, he was again unambiguous. He said the exception was characterised by ‘unlimited authority’ and the ‘suspension of the entire existing order’.^4^ In this way, he distinguished the exception from chaos or anarchy. The exception suspends the existing ordinary order but, in the exception, ‘order in the juristic sense still prevails even if it is not of the ordinary kind.’^5^ While, for Schmitt, the nature of sovereignty is power unconstrained by formal rules, the *point* of such power is to institute or ‘create a juridical order’ in response to the threat of chaos.^6^

However, Schmitt’s famous articulation of the relation between sovereignty and the exception emphasises not only the basis for a suspension of the law in a state of emergency. It also reveals the role of the sovereign in deciding upon the existence of the ‘normal situation’, the ‘everyday frame of life’, which the law requires to function.^7^ Schmitt rejected the idea that the ‘normal situation’ to which law applies can simply be presupposed.^8^ As Giorgio Agamben states, following Schmitt, ‘The law has a regulative character and is a “rule” not because it commands and proscribes, but because it must first of all create the sphere of its own reference in real life and *make that reference regular*.’^9^

For both Schmitt and Agamben, while ‘[t]he sovereign determines the norm; the norm neither determines nor constrains the sovereign’.^10^ That is, ‘legal order is what the sovereign makes of it.’^11^ In Schmitt’s own words: ‘There exists no norm that is applicable to chaos. For a legal order to make sense, a normal situation must exist, and he is sovereign who definitely decides whether this normal situation actually exists.’^12^ Thus, for Schmitt and Agamben, ‘the sovereign marks the point of indistinction between law and violence’; norm and decision; juridical order and factual order.^13^ The sovereign who decides on the exception belongs to, and stands outside, the ordinarily valid system.^14^ This is, according to Agamben, the paradox of sovereignty.^15^

While the sovereign may decide on exception and normality, Schmitt also implies that ‘the exception can be *good* for the legal order,’ by drawing attention to the rule and the importance of the ‘norm-bound regular situation’.^16^ As Tracy Strong puts it in his foreword to Schmitt’s *Political Theology*: ‘one can only have an exception if one has a rule. Therefore the designation of something as an exception is in fact an assertion of the nature and quality of the rule.’^17^ Where the situation demands an exception to the rule, the decision on the exception speaks to the value of the rule, but also to the rule’s nature as ‘human creation’—not automatically binding, not without exception.^18^ Further, where the exception demands a suspension of the law, the justification for such action is precisely the maintenance of the state in its ‘daily functioning’ and ‘everyday management’.^19^ For Agamben, interpreting Schmitt, while ‘[i]n the decision on the state of exception, the norm is suspended or even annulled’, what is *at issue* in that period of suspension is ‘the creation of a situation that makes the application of the norm possible’,^20^ or ‘the very condition of possibility of juridical rule’.^21^ This highlights the links in Schmitt’s theory between the nature of the political, the state, sovereignty, and the ineradicable humanness of the decision. ‘Convinced that the state is governed by the ever-present possibility of conflict, [Schmitt] held that resolute action’ in the decision on the exception ‘was necessary to combat threats, for the state’s *raison d’etre* was to maintain its integrity in order to ensure order and stability’.^22^ Gian Giacomo Fusco argues that the primary function of sovereign power, for Schmitt, is to act as a ‘normalising power’, aimed at ‘securing and stabilising the existential-normal dimension of the life of the community’.^23^ Thus, despite the centrality of the exception in Schmitt’s thinking, his obsession was stability.^24^ When Schmitt says there is no norm applicable to chaos, and thus entrusts the sovereign with deciding absolutely on the normal situation, one might at first perceive a determination to destroy the normative and the rule of law.^25^ But in the same work in which Schmitt locates his political theory in a ‘power that creates legal order and is always outside the law’,^26^ he also describes the ‘endeavor of a normal state’ as ‘assuring total peace within the state and its territory’ and identifies the creation of ‘tranquillity, security, and order’ as the prerequisite for the validity of legal norms.^27^

Schmitt spoke of the suspension of the entire existing order where the preservation of the possibility of the normal situation is exactly what is at stake. His theory arguably lends itself to being understood in formal, objective terms—where stability and order itself is more important than the underlying political foundation of that order.^28^ This is challenged to an extent when Schmitt’s theory is situated in its historical context, namely the period of ‘radical democratization’^29^ and dysfunctional parliamentary politics of the Weimar Republic. As Benjamin Schupmann reminds us, in Schmitt’s defence of the Weimar Constitution against extreme parties on the left and right seeking to ‘democratically revolutionize’ it, Schmitt identified within the constitution a fundamental ‘political decision to be a bourgeois *Rechtsstaat*^30^ which was above all oriented by its commitment to individual liberty’.^31^ Schmitt defined this political decision, ‘in the sense of bourgeois freedom’, also by reference to ‘private property, contractual liberty, and freedom of commerce and profession’.^32^ Schmitt railed against an interpretation of the constitution which, to him, would permit political parties not sharing in a commitment to this fundamental political status to legally seize power, even if it meant the destruction of the constitution and the state itself.^33^ Continuing the historical contextual analysis, Dimitrios Kivotidis explores Schmitt’s eventual turn to National Socialism and the transition of the Weimar State to the Nazi State.^34^ Kivotidis argues that the ostensibly formal decisionism of Schmitt’s theory is given ‘substantive content’ in his ultimate identification of a *specific enemy* (communism), which had to be defeated in defence of a *specific normative order* (bourgeois capitalism), and which, for Schmitt, ‘the new form of the Nazi state’ could achieve, while ‘the old form of the *Rechtsstaat*’ could not.^35^ What is revealed is the ‘indissoluble union’ between norm and exception, in that each represents only a different method to realise and defend the same underlying political order.^36^ Or as Kivotidis summarises: ‘The “normal” and the “exceptional” forms correspond to different historical situations, to different levels of intensification [of] socio-economic contradictions, *but they both serve the ultimate function of ensuring the reproduction of a regime of power, property and productive relations*.’^37^

In the current times of crisis and exception, the work of Schmitt seems both prescient and predominant. There is a growing body of literature examining states’ responses to the pandemic through the lens—directly or indirectly—of Schmitt and Agamben, along with Walter Benjamin, Michel Foucault, Roberto Esposito, and others.^38^ Even where these authors are not expressly engaged with, a focus of legal scholarship during the pandemic has been the use of emergency or exceptional powers and the related (re)assertion of, internationally, the sovereign state and, domestically, the dominance of the executive and its implications for democracy, human rights, and the rule of law.^39^

Exceptional measures in response to the crisis have manifested in various ways. These include constitutional mechanisms for the suspension of aspects of existing legal orders, including rights protections, as well as the use of pre-existing emergency legislation or newly created (or amended) statutes which, though they do not suspend the constitutional order (but rather operate within it), nonetheless expand the reach of law in the name of necessity.^40^ The latter has become more common in stable democratic regimes where, rather than using emergency constitutional powers, ordinary statutes grant extraordinary power to the executive, ostensibly, until the crisis has passed.^41^ Further, as Przemyslaw Tacik has demonstrated, the pandemic has seen forms of exceptionality that feature not so much in the formal suspension of the legal order but rather in taking statutory and regulatory measures that essentially ignore the existing hierarchy of legal norms.^42^

For some, the ‘hyper-normalisation’ of emergencies, a phenomenon traced to long before the pandemic, means that the state of exception is no longer a compelling question.^43^ It is, therefore, arguable that the COVID-19 pandemic is properly understood not as an aberration which will pass and allow normality to be restored, but rather ‘another stage in [a] continuous catastrophe’ where crisis *is* the norm.^44^ Further, as Tacik points out, what is interesting—and perhaps surprising considering the neoliberal economic orthodoxy of the last four decades which tends to be conceived of as a market ideology with a ‘negative relation to state power’^45^—is how the current nature of states in COVID-times sits rather well with contemporary capitalism.^46^ For example, despite the ‘extraordinary politico-legal weaponry’ used by governments in their responses to the health crisis, with all the attendant disruptions to trade and commerce, indeed precipitating a global recession, we have seen financial markets boom during the pandemic.^47^

It is through the theoretical lens set out above that I now turn to a consideration of the JobKeeper wage subsidy scheme: Australia’s flagship economic measure in response to the crisis and the single largest stimulus programme implemented in the nation’s history.

## Australia’s JobKeeper wage subsidy scheme

Sections 3, 4 and 5 provide an overview and analysis of the JobKeeper wage subsidy scheme. This section describes the legislative framework and basic operation of the scheme. Section 4 then focuses on the constitutional basis for the legislation, which locates the scheme in a jurisprudence of national crisis and emergency. Section 5 turns to the political narrative which accompanied JobKeeper’s introduction, emphasising both the exceptional nature of the scheme and its preservatory objects.

The first case of COVID-19 in Australia was announced by the Health Minister on 25 January 2020.^48^ In addition to the wide-ranging health measures, the fiscal stimulus deployed by Australia’s Commonwealth Government since the beginning of the crisis and through to FY2025 amounts to approximately $312 billion (AUD), or 15.75 per cent of 2020 GDP.^49^ This includes the government’s initial $17.6 billion response announced on 12 March 2020 comprising support for business investment by way of increases to depreciation and write-off tax deductions, one-off payments to households and small and medium-sized businesses to boost cash flow, as well as waivers of governmental fees and charges in certain industries.^50^ The relatively conventional first package was ramped up when second-stage measures were announced later in the month providing a further $66.1 billion in stimulus, including increases to social security and veteran support payments and an early release of superannuation scheme.^51^ The second round of stimulus also included the initial $715 million of assistance targeting the aviation sector through waivers and refunds of various fees and charges, later supplemented by subsidising regional and domestic routes.^52^ Since the beginning of the pandemic, the government’s assistance for the aviation sector alone has amounted to more than $5 billion.^53^ However, it was the government’s job retention scheme, JobKeeper (together with the doubling of the unemployment benefit through JobSeeker), which formed the ‘fiscal centrepiece’ of the state’s economic response to the pandemic.^54^

The Morrison Government announced JobKeeper on 30 March 2020. The joint media release of the Prime Minister and Treasurer described the scheme as a ‘historic wage subsidy’ which would benefit around six million workers in the form of ‘a flat payment of $1,500 per fortnight through their employer, before tax’.^55^ JobKeeper was given effect principally through the Coronavirus Economic Response Package (Payments and Benefits) Act 2020 (Cth) (the JobKeeper Act). The legislation was introduced and passed both houses on 8 April 2020, nine days after the scheme was announced.^56^ The object of the JobKeeper Act was to ‘provide financial support directly or indirectly to entities that are directly or indirectly affected by…COVID-19’.^57^ In short, the Act established a framework for the Treasurer^58^ and, by delegation, the Commissioner of Taxation (both part of the executive branch of government) to make the rules necessary or convenient for giving effect to the Act.^59^ The matters in respect of which the rules could make provision were framed broadly.^60^ These were justified in terms of the need for flexibility and responsiveness in the environment of uncertainty created by the pandemic and to allow the rules to be modified and updated as circumstances required.^61^

Under the original version of the scheme (JobKeeper 1.0), an entity qualified if it projected a decline in turnover of at least 30 per cent for a relevant period in 2020, relative to the same period in 2019.^62^ It was noteworthy that the decline in turnover test under JobKeeper 1.0 required only that the necessary decline be ‘projected’ by the entity. Further, once an entity met the decline in turnover test, there was no requirement to re-test in subsequent months.^63^ That is, once the decline in turnover test was met, the entity would qualify for payments until the end of the first iteration of the scheme, despite the possibility of its decline in turnover (projected or real) not meeting the 30 per cent threshold or indeed the possibility of turnover in fact surpassing 2019 figures.

The second iteration of the scheme (JobKeeper 2.0) was announced on 21 July 2020, extending JobKeeper by six months to 28 March 2021.^64^ The main changes under JobKeeper 2.0 related to business eligibility and the introduction of a two-tier payment system. In terms of business eligibility, from 28 September 2020 businesses seeking to claim JobKeeper were required to reassess their eligibility with reference to actual (rather than projected) turnover in the September and December 2020 quarters.^65^ That is, businesses would now need to show that their turnover had actually declined in those quarters relative to the corresponding quarter in 2019. The two-tier payment progressively stepped down the subsidy at different rates for eligible employees and business participants depending on the number of hours worked each week.^66^ The JobKeeper scheme was initially estimated to cost $130 billion, though by 22 May 2020 the Treasury and Australian Taxation Office had issued a joint statement taking a modest $60 billion off that initial estimate, leaving the government embarrassed and facing calls to widen the scheme’s reach given the gross overestimation.^67^ By the time the 2020–2021 Federal Budget was released on 06 October 2020, payments under JobKeeper totalled ‘around $60 billion’ and the government estimated the total cost (including the extension of the scheme) at $101.3 billion over 2019–2020 and 2020–2021.^68^ Recent reports put the total cost of the scheme at approximately $89 billion, making it the largest economic support programme in the country’s history.^69^

Australia’s economic recovery has been better than expected. After entering recession following falls in GDP of 0.3 per cent and a record 7 per cent in the March and June 2020 quarters respectively, by August 2020 there were signs of economic recovery and by the end of the year the country was ‘technically’ out of recession following economic growth over the last two quarters.^70^ Unemployment was a similar story, peaking at 7.5 per cent in July 2020 but recovering 93 per cent of losses by the beginning of 2021.^71^ By March 2021—just over a year after Australia’s first COVID-19 case—reports were that Australia’s V-shaped recovery was storming ahead thanks to the government’s ‘massive monetary and fiscal stimulus’,^72^ though some commentators were less optimistic at least with respect to certain criteria such as employment and wage growth.^73^ Approximately 18 months after Australia’s first case of the virus and the global disruption which followed (and continues), the Australian economy surpassed pre-pandemic levels.^74^

## JobKeeper and the nationhood power

JobKeeper, as law, does not arise from some constitutional or legislative declaration of emergency or crisis. However, its constitutional foundation does hearken back to Schmitt’s description of the exception as a peril or threat to the existence of the state.^75^ The constitutional basis for JobKeeper, in the context of Australia’s federation, is situated within a jurisprudence of executive power arising from the very existence of the state and the government’s competency for exercising its power for the benefit of the nation or to protect it from some perceived threat. The principal constitutional basis for the JobKeeper Act is expressed as relying on ‘the legislative power that the Parliament has under the Constitution with respect to matters that are *peculiarly adapted to the government of a nation and cannot otherwise be carried on for the benefit of the nation*’.^76^

In framing the constitutional basis this way, the government quoted from the judgment of Mason J in *Victoria v The Commonwealth and Hayden* (the *AAP* case).^77^ Mason J referred to the *Communist Party* case^78^ for the proposition that, apart from the enumerated heads of power in the Australian Constitution, the Commonwealth enjoys certain powers ‘which stem from its existence and its character as a polity’.^79^ His Honour noted that, up until then, it had not been suggested that the implied powers ‘extend beyond the area of internal security and protection of the State against disaffection and subversion’.^80^ Mason J was referring specifically to the judgment of Dixon J who took the view that ‘the power to legislate against subversive conduct has a source in principle that is deeper or wider than a series of combinations of the words of [Parliament’s incidental power under] s. 51(xxxix.) with those of other constitutional powers’.^81^ Intensifying the jurisprudence around s 61 of the Constitution (the basis for executive power of the Commonwealth),^82^ Mason J went further, adding:

in my opinion there is to be deduced *from the existence and character of the Commonwealth as a national government* and from the presence of ss. 51 (xxxix.) and 61 a capacity to engage in enterprises and activities *peculiarly adapted to the government of a nation and which cannot otherwise be carried on for the benefit of the nation*.^83^

Though four justices in the *AAP* case confirmed that s 61 of the Constitution incorporated an implied executive power stemming from the formation of the Commonwealth as a polity and national government, Mason J’s formulation was the most precise.^84^ The existence of this ‘nationhood power’^85^ and Mason J’s determinative criterion—the ‘peculiarly adapted’ test—was subsequently approved by the majority of the High Court in *Davis v The Commonwealth*^86^ and has been consistently applied by the High Court since.^87^ However, the circumstances in which the nationhood power might be enlivened and the scope of that power remain unresolved and the subject of scholarly consideration.^88^ Mason J only hinted at ‘enterprises and activities’, varying from time to time, which would be ‘appropriate to a national government’.^89^ Peta Stephenson, however, argues that the nationhood power has increasingly been defined ‘as a power to *protect the nation* and respond to *national emergencies*’.^90^

The case most analogous to JobKeeper is *Pape v Commissioner of Taxation* (*Pape*),^91^ which arose from the government’s response to the Global Financial Crisis (GFC). In that case, a majority of the High Court found that the executive power of the Commonwealth conferred by s 61 of the Constitution extended to the expenditure of public moneys to avoid or mitigate the effects of the GFC on the national economy. The legislative power ‘to support the exercise of the executive power’ in deploying the economic stimulus arose from the ‘incidental power conferred by s 51(xxxix) of the Constitution’.^92^ Specifically, the nationhood power was found to support stimulus payments of between $250 and $900 to eligible taxpayers.^93^ Though JobKeeper far exceeded the stimulus payments to taxpayers in response to the GFC, parallels can be drawn between the exceptional financial and economic conditions in which both programmes were implemented.

While noting that questions regarding the scope could not be answered in the compass of a single case, French CJ suggested that the executive and incidental legislative power extended to ‘short-term fiscal measures to meet adverse economic conditions affecting the nation as a whole, where such measures are on their face peculiarly within the capacity and resources of the Commonwealth Government’.^94^ The ‘urgent national economic problem’ was relevant for French CJ,^95^ though His Honour was wary of interfering with the constitutional distribution of powers and emphasised that the executive power in that particular case did not ‘equate it to a general power to manage the national economy’.^96^ Further, His Honour noted that whilst ‘national concern’ or ‘national emergency’ might be the ‘general rubric’ relative to the executive power, he found it unnecessary to identify a specific class or circumstances of events under that classification which would enliven the power.^97^

For the other majority justices, who recognised ‘a more expansive inherent national executive power in s 61’,^98^ the extraordinary global conditions which precipitated the use of that executive power were seemingly of greater significance. In determining whether the stimulus payments were supported under ss 61 and 51(xxxix), Gummow, Crennan, and Bell JJ said it was ‘necessary to ask whether determining that there is [a] need for an immediate fiscal stimulus to the national economy…falls within executive power’.^99^ Accepting that it could hardly be doubted that the GFC concerned Australia as a nation, Their Honours went on to draw an analogy between determining the need for fiscal stimulus in response to the crisis and determining a state of emergency following a natural disaster, concluding that ‘[t]he Executive Government is the arm of government capable of and empowered to respond to a crisis.’^100^ In doing so, Their Honours, first, justified the existence of such executive power ‘by reference to Australia’s status as a nation’; and second, characterised the fiscal measures deployed as necessary for the protection of the nation from the GFC.^101^ The centrality of the concept of national crisis or emergency was stated unequivocally. Applying Mason J’s test, Their Honours concluded: ‘in considering what enterprises and activities are peculiarly adapted to the government of the country and which cannot otherwise be carried on for its benefit, this case may be resolved *without going beyond the notions of national emergency* and the fiscal means of promptly responding to that situation.’^102^

After *Pape*, a majority of the High Court in *Williams v The Commonwealth*^103^ (*Williams (No 1)*) emphasised the importance of the element of national crisis or emergency, which element was found to be absent in the circumstances of that case.^104^ Hayne J was most explicit, describing the ‘species of executive power’ which was executed in *Pape* as ‘the determination of the existence of a national crisis or emergency’.^105^ Thus, the fiscal stimulus determined to be necessary in conditions of national crisis was an example of Mason J’s activities ‘peculiarly adapted’ to the Commonwealth Government and which otherwise could not be carried out for the public benefit.^106^ The other majority justices at least highlighted the essentiality of the economic and financial crisis in *Pape*, which distinguished it from the facts in *Williams (No 1)*.^107^ While distinguishing *Pape* in *CPCF v Minister for Immigration*,^108^ Hayne and Bell JJ described the relevant power as ‘an implied executive “nationhood power” *to respond to national emergencies*’.^109^

The considerations of nationhood and the associated limits of executive power may continue to be elusive,^110^ and indeed the merits of this implied executive power and its implications, especially for the distribution of powers, continue to be debated.^111^ However, Mason J’s ‘peculiarly adapted’ test has been firmly established, along with the concept of national crisis or emergency as at least an example of circumstances in which the nationhood power will be enlivened, if not an element essential to the exercise of that power.^112^ Of course, as Peter Gerangelos points out, if the ‘seriousness of the crisis…[is] the criterion for the valid exercise’ of the nationhood power, one might ask whether that criterion is best suited to judicial determination.^113^ Regardless, the government clearly had this jurisprudence in mind when it described the constitutional basis for the JobKeeper Act by reference to Mason J’s seminal judgment. Further, echoes of Schmitt’s theory of sovereignty can be detected in the High Court’s jurisprudence and the academic commentary on the nationhood power. Schmitt was concerned with the exceptional case—some ‘danger to the existence of the state’—which the positive legal order could neither predict nor overcome, thereby necessitating the sovereign who ‘decides whether there is an extreme emergency as well as what must be done to eliminate it’.^114^ For Schmitt, it is in the exception—where the ‘state suspends the law…on the basis of its right of self-preservation’—that the superiority of the state over the legal norm is undoubtedly proved.^115^ Of course, the emergencies in which the nationhood power has been exercised arguably fall short of Schmitt’s evocative ‘suspension of the entire existing order’.^116^ Nonetheless, in examining the power which comprised the constitutional foundation for JobKeeper, it is notable that such power: (*a*) arises from the existence and character of national government and an implied authority inextricably linked to *nationhood* and the ‘protection of the state’;^117^ (*b*) is enlivened by national crisis or emergency; and (*c*) is located within an authority (i.e., the executive government) precisely because of that authority’s *peculiar* capability to respond to the crisis. Finally, while it cannot be said that the nationhood power approaches the ‘unlimited authority’ of Schmitt’s sovereign,^118^ it is interesting that Mason J himself was cautious enough to note that an improper formulation of the power might threaten an aspect of Australia’s *legal order*, namely the distribution of power between the Federal and State Governments.^119^ He emphasised that the nationhood power would not be initiated by mere convenience, but should have jurisdiction only in truly *national* areas and outside the scope of the States’ legislative and executive competence.^120^

## Political narrative: Crisis, sovereignty, and order

The way in which a crisis is framed and the narrative constructed around it has implications for the state’s response to that crisis.^121^ Scott Morrison emphasised the dual nature of the COVID-19 crisis, in terms of health and the economy.^122^ Employing a device commonly adopted by states in public health crises,^123^ he also framed the pandemic as a threat to national security, to Australia’s very sovereignty.^124^ Arguably by doing so, the Prime Minister, at least oratorically, pushed the crisis above Schmitt’s threshold of ‘extreme peril, a danger to the existence of the state, or the like’.^125^ Following Schmitt’s theory, such a categorisation precipitates the suspension of the entire existing order, albeit not resulting in chaos but rather a new order of a different and unordinary kind.^126^ The preceding section has shown how, constitutionally, JobKeeper was born out of an executive (and incidental legislative) power, arising from the existence and character of the national government and actuated by national crisis or emergency. This section will consider the political narrative around JobKeeper and demonstrate how the crisis was characterised as a national peril and threat to sovereignty, while the policy-response was framed as a necessary exceptional measure to preserve and ultimately restore *normal* order. The initial parameters or guiding principles of the scheme will also be noted, to provide a theoretical baseline against which the reality of its roll-out might be compared, and context to the subsequent criticisms of JobKeeper.

When the Prime Minister announced on 27 February 2020 that ‘the risk of a global pandemic is very much upon us’ and that the government was implementing its emergency response plan, his language was noticeably measured.^127^ He pointed out that the response of the Australian Government was ‘well ahead’ of the World Health Organization (WHO).^128^ The health crisis was acknowledged, but the actions of the government in response were out of an ‘abundance of caution’, while any economic stimulus would be ‘targeted, modest and scalable’, with ‘broader larger fiscal stimulus-type responses’ described as being ‘quite the opposite’ of the then advice of the Treasury.^129^ Responding to ‘Dorothy Dixers’^130^ in Parliament, the Prime Minister noted that Australia was not immune, and coronavirus was a serious issue, but that Australia was as prepared as any country could be—Australia had got ahead of COVID-19 and was staying ahead.^131^

In the Prime Minister’s speech on 23 March 2020, the future was more foreboding. By this stage, the nation itself was facing a time of ‘great challenge’ and being put ‘to the test’, in response to which the Prime Minister appealed to the ‘Australian Spirit’, the legacy of the Anzacs, and the generations who survived the Great Depression.^132^ The Treasurer’s language had a militaristic flavour. COVID-19 was ‘an enemy without a flag or a face’ against whom ‘every weapon in our arsenal’ would be deployed; we were now ‘Team Australia’ and all sections of the community were called upon to ‘join this struggle’.^133^ In a comment which might have been directed at Schmitt himself, the Prime Minister even vowed to prove wrong those who would claim that liberal democracies were inept at responding to challenges of such magnitude.^134^

However, by the time the JobKeeper Act was introduced to Parliament, the rhetoric of national crisis and emergency had reached a new level. The times were no longer of great challenge, they were now ‘extreme’.^135^ The menace of the virus took on an anthropomorphic character as an enemy with whom the nation was in a ‘battle’ or a ‘fight’ for its very life.^136^ The nation was suddenly under threat and sovereignty became the central theme of the crisis.^137^ That sovereignty, according to the Prime Minister, is ‘measured in our capacity and freedom to live our lives as we choose in a free, open and democratic society’ and ‘enabled by having a vibrant market economy…that gives all Australians the opportunity to fulfill their potential’.^138^ This was something, he said, ‘we will not *surrender*’.^139^ Deliverance would be achieved through our collective support of the national interest and, in the spirit of sacrifice, the ‘great cost’ of protecting our sovereignty would be willingly paid.^140^ The Explanatory Memorandum accompanying the JobKeeper Bill included similar language of crisis and nationalism. Though the document spoke largely to the framework of, and justifications for, the stimulus package itself, JobKeeper was described as part of the government’s response to the ‘potential national emergency’ arising from the pandemic.^141^ The actions of the government were ‘decisive’ in a time of uncertainty and taken in the ‘national interest’.^142^ This political framing is important. The wartime analogy and rhetoric of the nation under threat seemed to serve as the foundation to enable and justify, if not a complete suspension of the economic order, at least a suspension of existing ideological orthodoxy to permit a state intervention and a reorienting of the economy toward a political objective.^143^

Emphasising the indiscriminate nature of a threat which demanded a unified response, the Prime Minister claimed that political ideology was being put aside: ‘make no mistake: today is not about ideologies. We checked those in at the door. Today is about defending and protecting Australia’s national sovereignty.’^144^ Citing advice from former Prime Minister John Howard, who arguably led the country’s most hard-line neoliberal government,^145^ the Treasurer similarly freed himself of ideological constraints.^146^ The contradiction of the government’s historic intervention in the economy compared with its historical philosophical position was not missed by the Leader of the Opposition or the media.^147^ Though Scott Morrison’s comment was clearly meant as a self-granted licence to adopt whatever policy the situation demanded, free from ideological shackles, one could hardly say ideology was truly checked at the door. Familiar political and economic ideology plainly informed the Prime Minister’s framing of ‘our sovereignty’ when he described it as being ‘enabled by having a vibrant market economy’ which allowed all individuals to fulfil their potential.^148^

The political discourse discussed above suggests that the threat of the pandemic and its economic implications was used to justify a suspension of at least the normal (neoliberal) politico-economic order and the use of exceptional measures at odds with the government’s traditional ideological position. Certainly, that seemed to be the Prime Minister’s personal reconciliation of his government’s historic intervention. In recent statements (as of writing this article), the Prime Minister proclaimed that ‘telling [people] what to do’ was wholly inconsistent with his government’s ideological foundations: ‘That approach does not come easy to us…It just doesn’t. Every fibre of our being in many of the decisions we’ve had to take, it goes against our instincts, it goes against our grain’.^149^ However, consistent with Schmitt’s and Agamben’s *preservatory* conception of the measures taken in the decision on the exception, the Prime Minister also made clear that he had the bigger picture, the bigger *order* in mind when he added that, notwithstanding the *extraordinary* interventions of his government, ‘*it was necessary*’ in the face of crisis.^150^ The Treasurer employed biblical terms, describing the crisis as an ‘economic armageddon’ which the exceptional economic support measures had prevented.^151^ Such comments speak to the prevalence of the concepts theorised by Schmitt and Agamben and their emphasis on the mutually constitutive relationship between norm and exception.

However, to the extent that Schmitt’s theory of the exceptional case is characterised by ‘unlimited power’,^152^ it must be said that the political narrative which accompanied the government’s economic intervention suggests something less than that, in the sense that certain guiding principles and parameters were discernible. If the government was laying the foundation for exceptional measures to preserve at least an *unordinary* order during the crisis, it had some idea of how JobKeeper, as an exceptional measure, was intended to work and what it was intended to achieve. According to the statute itself, the object of JobKeeper was to ‘provide financial support directly or indirectly to entities that are directly or indirectly affected by…COVID-19’.^153^ This object points to the first of three themes which largely dominated the political narrative accompanying the introduction of JobKeeper, namely that the measures introduced by the government would be targeted, temporary, and proportionate.

Needless to say, the target of the scheme is implicit in the title of the legislation: JobKeeper was aimed at workers and the businesses which employed them. The scheme was promoted as a support measure for businesses affected by the pandemic to cover the cost of wages, so that employees would retain jobs and maintain incomes.^154^ Acknowledging the dual nature of the crisis not only for health but the economy also,^155^ the scheme was intended as a lifeline for businesses struggling to retain employees in the face of the disruption caused by the pandemic.^156^ Despite the obvious link between business and jobs, the primacy of the worker in the political narrative could hardly have been mistaken when the Treasurer introduced the JobKeeper Bill to Parliament, announcing ‘Today is the day that this Bill saves millions of Australian jobs. Today is the day that the people’s house delivers for the Australian people.’^157^

The narrative also regularly reiterated the temporally limited nature of the scheme. Indeed, the temporary nature of JobKeeper, as a response to a crisis expected to eventually pass, partly explains the willingness of a conservative government to roll out a historic welfare policy.^158^ JobKeeper was about assisting businesses and their employees *through* the economic downturn brought about by lockdowns and shutdowns.^159^ It anticipated the end of the crisis, by promising to help maintain the connection between employers and employees so that businesses could ‘reactivate their operations quickly’ when the crisis was over.^160^ JobKeeper might have been unprecedented both in the degree of government intervention and its political palatability,^161^ but the *exceptional and temporary* nature of the policy was underscored by the Prime Minister when he described it, along with other stimulus measures, as an ‘economic bridge’ for workers and businesses and reminded us that the pandemic and ‘not the economy’ was the problem.^162^ The bridge is a fitting metaphor for a measure designed to allow the nation to safely pass over a dangerous obstacle, but continue essentially along the same path, or perhaps pass back over that bridge. According to John Quiggin, the assumption of the crisis as an aberration, followed by a recovery and ‘return to normality’, is typical of governments and central banks in all crises since the late 1970s.^163^

What the government meant when it described its response measures as being ‘proportionate’ is perhaps more ambiguous. The Treasurer provided some assistance when he referred to the government’s guiding principles as shaping the response, which would be proportionate and ‘scalable to the challenges we face’.^164^ Taken literally, the scale of the response might be virtually unlimited, given the global nature of the pandemic and its historic disruption to the world economy. However, the proportionality of the scheme is arguably best understood by reference to the characteristic unequivocally attached to the businesses which would be targeted by it. That is, the explanation for the scheme, indeed the eligibility criteria themselves, suggested *only* certain businesses—namely those which were ‘struggling’, ‘significantly impacted’, which were suffering a substantial decline in turnover—were the intended beneficiaries.^165^ When the scheme was extended under JobKeeper 2.0, it targeted those ‘that [needed] assistance the most’.^166^ This clearly suggested the limits of the scheme would be determined and indeed constrained by reference to the extent of the genuine impact of the pandemic on businesses.

## JobKeeper as corporate welfare

Understood as a wage subsidy which would support businesses and their workers significantly impacted by the pandemic, JobKeeper was widely supported. The JobKeeper Act passed both houses of Parliament with bipartisan support. At its peak, the scheme supported approximately 3.5 million individuals and over 900,000 organisations.^167^ However, with the passage of time, it has also been criticised as corporate welfare for channelling billions of dollars of public money into the hands of corporations and their investors.^168^ From as early as June 2020 the Treasury flagged potential changes to JobKeeper arising from the looseness of eligibility criteria. While noting that the scheme had achieved its objectives and was supposedly ‘well targeted’ towards businesses which had suffered a decline under the pandemic, the government’s three-month review identified matters which might need reconsideration in any future changes to the scheme, including eligibility being based on projected (rather than actual) decline in turnover and the fact that eligibility requirements needed only to be met once rather than on some regular basis.^169^

In February 2021, there was already media interest in companies which had received JobKeeper payments despite remaining profitable and even paying multimillion dollar dividends and executive bonuses.^170^ At the same time it was announced that the Auditor General would investigate the operation of the scheme, examining whether the rules had been effectively administered and whether appropriate measures had been implemented to protect the integrity of the scheme.^171^ Public servants responsible for its administration highlighted that those businesses which took advantage of the scheme, despite remaining profitable, were operating within the law as it was designed, if perhaps not true to its spirit. In response to questions from the Senate Select Committee on COVID-19 regarding businesses which had, despite the pandemic, profited and paid dividends or bonuses, Australian Taxation Office officials pointed out that once a business had qualified, that the same business might subsequently enjoy boon times was ‘irrelevant to their entitlements in JobKeeper 1’.^172^

A flurry of media attention followed in March 2021 after wide reporting of an analysis by governance advisory firm Ownership Matters of ASX300 companies which received JobKeeper and other subsidies (the OM report).^173^ Most notably, the OM report revealed that one quarter of ASX300 listed companies disclosed receiving more than $2.4 billion in JobKeeper payments in the 2020 calendar year, while in the half-year to 31 December 2020 more than $1 billion went to companies which reported positive earnings metrics or an increase to earnings from pre-pandemic levels.^174^ In a particularly pointed comparison, it was reported that JobKeeper payments received by seven ASX300 companies in the half-year July–December 2020 equated to more than 50 per cent of the dividends paid by each of those companies.^175^ In the case of Eagers Automotive Ltd, dividends of more than $64 million were proposed in its 2020 annual report, after receiving over $67 million in JobKeeper payments during the half-year to December 2020 (approximately 104 per cent of the dividend payment).^176^ Subsequently, it was reported following analysis conducted by request from the Shadow Assistant Minister for Treasury^177^ that investors in companies which received JobKeeper enjoyed returns over 40 per cent greater than investors in companies which did not receive the subsidy.^178^ Billionaires Gerry Harvey, Solomon Lew, and Nick Politis were named and shamed in news articles for personally pocketing dividends worth $78 million, $24.25 million, and $17 million respectively, after their companies received around $22 million, $70 million, and $130 million respectively in JobKeeper payments.^179^ More recent analysis from the Parliamentary Budget Office^180^ confirmed that at least $38 billion went to companies whose turnover, despite their projections, did not fall by the requisite threshold, while $2.6 billion went to companies which saw quarterly turnover double or triple compared with 2019.^181^

Public scrutiny led a number of companies which received JobKeeper payments but remained profitable to pledge their intention to return part or all of the wage subsidies received, including Adairs, Nick Scali, Toyota, Domino’s, Super Retail Group, Cochlear, Santos, Nine Entertainment, Seek, and Blackmores.^182^ Others, including Harvey Norman, Eagers Automotive, and Accent Group, refused.^183^ When Scott Morrison was asked whether ‘concrete action’ would be taken to recoup JobKeeper subsidies converted by profitable corporations into dividends and executive bonuses, the Prime Minister dismissed the question, stating that he was not into the ‘politics of envy’, and issued the reminder that ten months earlier ‘we were staring into the abyss’ and that JobKeeper, despite such criticisms, changed ‘the course of the nation’.^184^ The Treasurer expressed similar sentiments in response to scrutiny of the scheme, emphasising that JobKeeper ‘saved lives and livelihoods…during the greatest economic shock since the Great Depression’.^185^ For the Prime Minister and Treasurer, as well as in the Treasury’s own review and in some media reports, the initial framing of the scheme as saving jobs and businesses struggling in the pandemic via targeted, temporary, and proportionate support (discussed above) was de-emphasised, while the macroeconomic benefits of JobKeeper—its positive effect on business confidence, the ‘certainty’ it provided in an uncertain environment, and its ‘economy-saving’ effectiveness—were brought to the fore.^186^

The Australian scheme was distinguishable due to the absence of a public register of recipients and the triggering of employer entitlements by (initially) an *anticipated* downturn in turnover, compared with other models such as the UK and New Zealand (which paid a portion of the wages of stood-down workers).^187^ That the public has the benefit of the analyses conducted by the Parliamentary Budget Office is due almost entirely to a Shadow Assistant Minister having made such requests, though a policy costing in relation to recouping JobKeeper payments from certain publicly listed companies was also requested by the Australian Greens.^188^ Citing the lack of ‘transparency’ around the scheme and the delivery of ‘public money that was meant to be for workers’ wages’ into the hands of corporations and their ‘super wealthy directors’, the Greens’ Deputy Leader Senator Nicholas McKim introduced the Coronavirus Economic Response Package Amendment (Ending JobKeeper Profiteering) Bill 2021.^189^ The Bill sought to amend the JobKeeper Act by introducing a recovery mechanism, applicable where entities with a turnover of more than $50 million made a profit, or paid dividends or executive bonuses.^190^ Unless the entity repaid the amount received in JobKeeper subsidies, or the ‘relevant profit’ (defined as profit, dividends, or bonuses) where such amount was less than the amount received in JobKeeper, then the entity would be prevented from claiming input tax credits for a period of up to ten years.^191^ The Bill would also enact provisions compelling the Commissioner of Taxation to maintain a public register with information in relation to entities captured by the amended legislation, including the name of the entity, the amount received in JobKeeper payments, and details regarding any repayments.^192^

The Greens’ Bill was referred to the Economics Legislation Committee for consideration. The window for public submissions was just 15 days and only seven submissions were made to the inquiry and published.^193^ A common theme among them was the intended purpose of JobKeeper in supporting workers, the unmet need for transparency, and the influencing effect of public scrutiny on the decisions of some corporations to repay part or all of JobKeeper.^194^ Domino’s Pizza, for example, supported the proposed public register as being in line with ‘community expectations’, while expressing reservations about retrospectively changing eligibility rules and the impact such might have on take-up of future government programmes and their economic impact.^195^ While all of the seven submissions were wholly or partly supportive of the Bill, or at least seemingly neutral or not opposed,^196^ a submission from economists Rabee Tourky and Rohan Pitchford of the Australian National University argued that the proposed retrospective intervention ought to go further by imposing a direct and involuntary repayment mechanism and apply to more than just the large corporations targeted by the Bill.^197^ Ultimately, the Committee recommended that the Bill not be passed, noting its belief that ‘JobKeeper was an unambiguously successful program.’^198^ Two reasons for the recommendation were emphasised in the Committee’s conclusion. On the question of the potential clawback mechanism, the retrospective nature of the legislation was opposed.^199^ On the question of the public register, while the Committee acknowledged the need for ‘transparency of government processes to ensure accountability’, its concern around the inclusion of the catch-all words ‘further information’ in the relevant provision requiring the Commissioner to publish information on JobKeeper ‘profiteers’, proved to be a deciding factor.^200^ An ironically minor concern about the implications of a lack of specificity, given the structure of Australia’s largest ever economic support programme.

## JobKeeper as exception*norm*

As described above, the legalities and parameters of JobKeeper have faced criticism on a number of fronts, particularly with the benefit of hindsight. Critiques of the structure of the scheme have gone to the generous nature of the eligibility criteria, including, in its first iteration, the use of a projected reduction in turnover and the lack of any conditions or mutual obligations attached to the subsidy, such as an obligation to return subsidies, or a mechanism to recoup them, if they proved to be unnecessary. In addition, unlike comparable schemes in other countries, the Australian Government did not include a public register of employers who received the subsidy, meaning there has been limited transparency around who received benefits.^201^ The result has been that some companies and their shareholders have received significant windfalls, with limited ability for public scrutiny. This would appear to be at odds with the spirit and (at least original) intent of the scheme as a worker-focussed, job-saving measure. It should be noted, though, that the scheme did require the continued employment of workers to qualify for the subsidy. So, in that sense, it ensured continued employment even if for many this might have meant that working hours were reduced to become equivalent to what the firms received from the subsidy, while for others their employment apparently might never have been in jeopardy anyway.

However, if we return to the contextual framing of the programme as a tool of exceptionality, it invites us to consider JobKeeper through the theoretical lens set out in section 2 and to explore Schmitt’s conception of the relationship between norm and exception and the ultimate function of normative and exceptional powers. Schmitt described a suspension of the norm in the state of exception, but two points must be re-emphasised. First, that the impetus for that suspension is the *preservation* or *realisation* of the ‘normal situation’ necessary for a legal order to make sense.^202^ Further, as Kivotidis and Schupmann have pointed out, Schmitt himself had in mind not just *any* objective order, but rather a bourgeois capitalist legal state, committed to individual liberty, private property rights, and freedom of contract.^203^ Second, in the exception a kind of *un*ordinary order still prevails.^204^ Agamben describes an ‘intimate cohesion’ between the norm and its realisation or concrete application, which reaches its greatest intensity in the exception.^205^ This cohesion is exemplified by the view that at the heart of the suspension of the normal order in the state of exception is the very preservation of the possibility of the normal situation. As noted previously, Kivotidis refers to the ‘unity’ and common purpose of norm and exception by suggesting that each is a different form of public power, but where *both* are essential ‘for the reproduction of bourgeois rule’—the form that is employed being ‘contingent upon the intensification of socio-economic antagonisms’.^206^ This allows us to consider the extent to which JobKeeper might be understood as an exceptional measure, an exceptional form of public power, utilised during the historic socio-economic disruption of the pandemic, in order to reaffirm a fundamental underlying politico-economic order. In turn we can re-evaluate the criticisms of the scheme and see that—rather than merely pointing out deficiencies or unintended failings of JobKeeper—such criticisms in fact underscore the *un*exceptional and *normalising* outcomes of the neoliberal political and economic order JobKeeper sought to preserve.

As has already been noted, JobKeeper was an exceptional policy, but it was not *extra-legal* in the sense of being outside of or wholly suspending the legal order in the way Schmitt described. That said, JobKeeper arguably fits within Aihwa Ong’s broadening of the concept of the exception, beyond Schmitt’s suspension of ‘generalized political normativity’ to an ‘*extraordinary departure in policy* that can be deployed to *include* as well as to exclude’ and *allocate value* within society.^207^ The Prime Minister made clear that JobKeeper, as his government’s policy, was extraordinary when he declared on the day it was introduced to Parliament that ideology had been put aside.^208^ In terms of allocating value, whatever JobKeeper’s broader benefits for the Australian economy, thanks to the analyses from the OM Report and the Parliamentary Budget Office we have seen how JobKeeper became a tailwind to profit for many large companies and added to the wealth of already wealthy investors and executive managers. There is nothing *exceptional* about this outcome. JobKeeper was overwhelmingly deployed *through* the corporate form which—seen under the conditions of neoliberalism as a private association of individuals based on a ‘nexus of contracts’—prioritises shareholder value maximisation.^209^

Thus, whilst one may commend the position of companies such as Toyota and Domino’s in returning what turned out to be unnecessary financial support, the basis for doing so becomes important, particularly where there is apparently no legal obligation (as the Treasurer made clear),^210^ but only a social or moral one. Under the neoliberal paradigm of shareholder primacy, which insists ‘that company earnings should be returned to shareholders as much as possible’,^211^ one could argue that, notwithstanding the exceptional conditions of the pandemic or the spirit in which the assistance was provided, if there exists no legal obligation to give back the funds, then the company’s obligation *is* to keep funds legally received *and* distribute as much profits to shareholders as possible. The limitation to this would only appear to be the tradition of ‘enlightened shareholder value’, according to which the potential impact on a company’s reputation *may* require actions that are not so immediately self-serving. Such would appear to be at least partially effective. For example, Harvey Norman eventually agreed to repay at least some of its JobKeeper subsidies in response to sustained public pressure and (seemingly) to protect the corporate reputation.^212^ Corporations, mostly publicly listed, have pledged to return more than $225 million in unneeded JobKeeper subsidy payments.^213^ But for the vast majority of companies which shared in $38 billion in JobKeeper subsidies, despite their decline in turnover not reaching the projected threshold, that such financial assistance may be realised as profits and converted to returns for investors represents only the *ordinary* functioning of the neoliberal corporate order, albeit under *extraordinary* socio-economic conditions.

While perceived ‘profiteering’ from JobKeeper might be the predictable result of deploying financial assistance through the corporate form, it is interesting to note that a concern with *order* has also coloured the resistance to attempts to rectify the perceived deficiencies of the scheme, such as through the Greens’ Bill discussed in section 6. The apprehension about taking up such approaches is itself framed in terms of stability and order.^214^ That is, a retrospective ‘changing of the rules’, it is thought, would potentially impact any future take-up of government programmes and limit their effectiveness, while a public register would infringe on corporations’ expectations and rights in relation to privacy. Whereas the deployment of the subsidy was an exceptional measure intended as a bridge towards some degree of economic normality, the proposed turn to retrospectivity is itself cast as a tool of exceptionality—a breach or suspension of a key principle of the rule of law which might undermine the very stability it is supposed to provide (and which JobKeeper was intended to return us to). Here one form of exceptionality—an exceptional measure aimed at returning us to the norm—is pitched against another, namely the need for the rule of law to apply in a consistent fashion (even if the companies in question would not appear to have adhered to the intent or spirit of that law).

As a final point on the relationship between norm and exception and the ultimate function of JobKeeper, consider the connection between crises and the neoliberal corporation. The rise of neoliberal corporate governance promoted not only the return of profits and retained earnings to shareholders but also shifted the corporation’s funding of investment, innovation, and development *away from* those retained earnings and towards cheaply sourced loans and debts.^215^ As was seen during the GFC, this often left companies at the mercy of changes in markets—when markets were disrupted, social bail-outs were needed.^216^ What both JobKeeper and the pandemic have revealed, however, is the way in which this paradigm also *presumes* the inability of corporations to be able to sustain themselves through periods of disruption. That is, it *normalises* the need for government support in order to sustain the employment of workers, rather than relying on retained earnings and capital reserves which could and possibly should be drawn upon in economic (and other) crises. Whereas shareholders have benefited from the return of profits for many years, it seems a threatened economic crisis—cast in catastrophic terms but ultimately measured by a more anaemic-sounding downturn in corporate turnover of 30 per cent (whether projected or otherwise)—supposedly warrants financial support from the government to ensure the continued employment of workers. This in turn reaffirms and entrenches the reliance of firms on governments to provide some sense of *normality*, or *normal economic order*, in which they can sustain themselves.

## Conclusion

This article has analysed Australia’s flagship economic-policy response to the pandemic, the JobKeeper wage subsidy scheme. JobKeeper has been examined by drawing upon the ideas of Carl Schmitt and Giorgio Agamben, particularly as to the mutually constitutive relationship between norm and exception and the idea of the exceptional measure as a crisis-specific form for securing and reproducing a fundamental underlying order. Viewed through this lens, I have highlighted that JobKeeper’s legislative foundation was based in nationhood, sovereignty, and the need for exceptional measures in the face of crisis, while its accompanying political narrative emphasised the same themes, as well as the scheme’s preservatory objects. The outcomes of Australia’s historic wage subsidy scheme were considered, particularly critiques of JobKeeper as corporate welfare which delivered billions of dollars to companies and their investors. However, rather than simply highlighting deficiencies of the JobKeeper programme, these outcomes actually underscore its ultimate function and demonstrate that the scheme, though an extraordinary departure from policy, can be understood as fundamentally a different and exceptional method to secure and reproduce our neoliberal corporate order in a state of exception. That is, the outcomes of the implementation of the scheme which have attracted justifiable criticism are simply symptomatic of the paradigm of our neoliberal political and economic order. To the extent such outcomes can and should be challenged, what is needed is therefore not a ‘return to normal’ but rather a more fundamental disruption to that enduring order.

## Supplementary Information

Below is the link to the electronic supplementary material.

Supplementary file1 (PDF 5423 kb)

Supplementary file2 (PDF 1979 kb)

Supplementary file3 (PDF 397 kb)

## Footnotes

1 Carl Schmitt, *Political Theology: Four Chapters on the Concept of Sovereignty* (George Schwab tr, University of Chicago Press 2005) 5.

2 Jessica Whyte, *Catastrophe and Redemption: The Political Thought of Giorgio Agamben* (State University of New York Press 2013) 58; emphasis added.

3 Schmitt, *Political Theology* (n 1) 6.

4 Ibid. 12.

5 Ibid.

6 Ibid. xx (Foreword by Tracy B Strong).

7 Ibid. 13.

8 Jens Meierhenrich and Oliver Simons, ‘“A Fanatic of Order in an Epoch of Confusing Turmoil”: The Political, Legal, and Cultural Thought of Carl Schmitt’ in Jens Meierhenrich and Oliver Simons (eds), *The Oxford Handbook of Carl Schmitt* (Oxford University Press 2016) 35.

9 Giorgio Agamben, *The Omnibus Homo Sacer* (Daniel Heller-Roazen et al. trs, Stanford University Press 2017) 25.

10 Whyte, *Catastrophe and Redemption* (n 2) 58.

11 Meierhenrich and Simons, ‘A Fanatic of Order in an Epoch of Confusing Turmoil’ (n 8) 35.

12 Schmitt, *Political Theology* (n 1) 13.

13 Whyte, *Catastrophe and Redemption* (n 2) 58.

14 Schmitt, *Political Theology* (n 1) 7.

15 Agamben, *The Omnibus Homo Sacer* (n 9) 17.

16 John P McCormick, *Carl Schmitt’s Critique of Liberalism: Against Politics as Technology* (Cambridge University Press 1997) 226–227. Though, Schmitt gives pre-eminence to the exception as that which first brings about and legitimates the normal order or situation which makes law possible and ‘proves everything’. See Schmitt, *Political Theology* (n 1) 13, 15.

17 Schmitt, *Political Theology* (n 1) xxi (Foreword by Tracy Strong).

18 Ibid.

19 Michel Foucault, *Security, Territory, Population: Lectures at the Collège de France 1977–1978* (Michel Senellart ed, Graham Burchell tr, Palgrave Macmillan 2009) 238.

20 Agamben, *The Omnibus Homo Sacer* (n 9) 196.

21 Ibid. 18.

22 Schmitt, *Political Theology* (n 1), ‘Introduction’ by George Schwab, xlii; emphasis added.

23 Gian Giacomo Fusco, ‘Normalising Sovereignty: Reflections of Schmitt’s Notions of Exception, Decision and Normality’ (2017) 26(1) *Griffith Law Review* 128, 129.

24 Schmitt, *Political Theology* (n 1), ‘Introduction’ by George Schwab, li.

25 Ellen Kennedy, *Constitutional Failure: Carl Schmitt in Weimar* (Duke University Press 2004) 85; Fionnuala Ní Aoláin and Oren Gross, *Law in Times of Crisis: Emergency Powers in Theory and Practice* (Cambridge University Press 2006) 164.

26 Kennedy, *Constitutional Failure* (n 25) 85–86.

27 Carl Schmitt, *The Concept of the Political* (George Schwab tr, University of Chicago Press 2007) 46.

28 Benjamin Schupmann, *Carl Schmitt’s State and Constitutional Theory: A Critical Analysis* (Oxford University Press 2017) 173–175.

29 Bonn Juego, ‘Authoritarian Neoliberalism: Its Ideological Antecedents and Policy Manifestations from Carl Schmitt’s Political Economy of Governance’ (2018) 19(1) *Administrative Culture* 105, 116.

30 That is, a legal state or state governed by the rule of law.

31 Schupmann, *Carl Schmitt’s State and Constitutional Theory* (n 28) 177, 180.

32 Carl Schmitt, *Constitutional Theory* (Jeffrey Seitzer tr, Duke University Press 2008) 169.

33 Schupmann, *Carl Schmitt’s State and Constitutional Theory* (n 28) 178–179.

34 Dimitrios Kivotidis, ‘“Norm” and “Exception”: From the Weimar Republic to the Nazi State Form’ in Cosmin Cercel, Gian Giacomo Fuso, and Simon Lavis (eds), *States of Exception: Law, History, Theory* (Routledge 2021).

35 Ibid. 122–125.

36 Ibid. 138.

37 Kivotidis, ‘“Norm” and “Exception”’ (n 34) 138; emphasis added.

38 See, e.g., Przemyslaw Tacik, ‘The Blizzard of the World: COVID-19 and the Last Say of the State of Exception’ (2021) 96 *Folia Iuridica* 17; Catherine Larocque and Thomas Foth, ‘Which Lives Are Worth Saving? Biolegitimacy and Harm Reduction during COVID-19’ (2021) 28(4) *Nursing Inquiry* e12417; Btihaj Ajana, ‘Immunitarianism: Defence and Sacrifice in the Politics of Covid-19’ (2021) 43(1) *History and Philosophy of the Life Sciences* 25; Guillermo Andrés Duque Silva and Cristina Del Prado Higuera, ‘Political Theology and COVID-19: Agamben’s Critique of Science as a New “Pandemic Religion”’ (2021) 7(1) *Open Theology* 501.

39 See, e.g., Matthew Flinders, ‘Democracy and the Politics of Coronavirus: Trust, Blame and Understanding’ (2020) *Parliamentary Affairs* gsaa013; Tacik, ‘The Blizzard of the World’ (n 38); Cercel et al., *States of Exception* (n 34); Victor V Ramraj (ed), *COVID-19 in Asia: Law and Policy Contexts* (Oxford University Press 2021) Part 2; Kylie Evans and Nicholas Petrie, ‘COVID-19 and the Australian Human Rights Acts’ (2020) 45(3) *Alternative Law Journal* 175.

40 See, e.g., Evans and Petrie, ‘COVID-19 and the Australian Human Rights Acts’ (n 39); Cercel et al., *States of Exception* (n 34); Tacik, ‘The Blizzard of the World’ (n 38).

41 Gian Giacomo Fusco, Cosmin Cercel, and Simon Lavis, ‘Introduction: Untimely Considerations on the State of Exception’ in Cercel et al., *States of Exception* (n 34) 5.

42 Tacik, ‘The Blizzard of the World’ (n 38) 23.

43 Fusco et al., ‘Introduction’ (n 41) 1.

44 Tacik, ‘The Blizzard of the World’ (n 38) 18.

45 Aihwa Ong, *Neoliberalism as Exception: Mutations in Citizenship and Sovereignty* (Duke University Press 2006) 3.

46 Tacik, ‘The Blizzard of the World’ (n 38) 17.

47 Ibid.

48 Australian Government, ‘Media Release: First Confirmed Case of Novel Coronavirus in Australia’ (25 January 2020). www.health.gov.au/ministers/the-hon-greg-hunt-mp/media/first-confirmed-case-of-novel-coronavirus-in-australia. Accessed 24 July 2021.

49 International Monetary Fund, ‘Policy Responses to COVID-19’ (2021). www.imf.org/en/Topics/imf-and-covid19/Policy-Responses-to-COVID-19. Accessed 23 June 2021.

50 Prime Minister of Australia, ‘Media Release: Economic Stimulus Package’ (12 March 2020). www.pm.gov.au/media/economic-stimulus-package. Accessed 23 June 2021.

51 Prime Minister of Australia, ‘Media Release: Supporting Australian Workers and Business’ (22 March 2020). www.pm.gov.au/media/supporting-australian-workers-and-business. Accessed 23 June 2021.

52 Australian Government, ‘Aviation’ (07 May 2021). www.infrastructure.gov.au/infrastructure-transport-vehicles/aviation. Accessed 23 June 2021.

53 The Hon Barnaby Joyce MP, ‘Media Release: Readying Australia’s International Aviation Industry for Take-Off’ (20 September 2021). https://minister.infrastructure.gov.au/joyce/media-release/readying-australias-international-aviation-industry-take. Accessed 25 October 2021.

54 Ben Spies-Butcher, ‘The Temporary Welfare State: The Political Economy of JobKeeper, JobSeeker and “Snap Back”’ (2020) 85 *Journal of Australian Political Economy* 155, 155.

55 Prime Minister of Australia, ‘Media Release: $130 Billion JobKeeper Payment to Keep Australians in a Job’ (30 March 2020). www.pm.gov.au/media/130-billion-jobkeeper-payment-keep-australians-job. Accessed 23 June 2021.

56 Parliament of Australia, ‘Coronavirus Economic Response Package (Payments and Benefits) Bill 2020’ (08 April 2020). www.aph.gov.au/Parliamentary_Business/Bills_Legislation/Bills_Search_Results/Result?bId=r6533#. Accessed 07 April 2022.

57 Coronavirus Economic Response Package (Payments and Benefits) Act 2020 (Cth) s 3.

58 The Treasurer is the government minister responsible for the department of the Treasury.

59 Coronavirus Economic Response Package (Payments and Benefits) Act 2020 (Cth) s 20(1).

60 Ibid. s 7.

61 Explanatory Memorandum, Coronavirus Economic Response Package (Payments and Benefits) Bill 2020 (Cth) and Coronavirus Economic Response Package Omnibus (Measures No. 2) Bill 2020 (Cth) para 2.69.

62 Coronavirus Economic Response Package (Payments and Benefits) Rules 2020 (Cth) ss 7–8. The scheme applied to entities which on 01 March 2020 carried on a business in Australia, or were other eligible entities as of that date, including non-profit bodies that pursued their objectives principally in Australia and eligible deductible gift recipients. The relevant decline in turnover was at least 15 per cent for qualifying charities, or at least 50 per cent for entities with an aggregated turnover of $1 billion or more.

63 Explanatory Statement, Coronavirus Economic Response Package (Payments and Benefits) Rules 2020 (Cth) 6.

64 Prime Minister of Australia, ‘Media Release: JobKeeper Payment and Income Support Extended’ (21 July 2020). www.pm.gov.au/media/jobkeeper-payment-and-income-support-extended. Accessed 23 September 2021.

65 Coronavirus Economic Response Package (Payments and Benefits) Rules 2020 (Cth) s 8B.

66 Ibid. s 13.

67 Australian Government, ‘Media Release: Joint Treasury and ATO Statement—JobKeeper Update’ (22 May 2020). www.ato.gov.au/Media-centre/Media-releases/Joint-Treasury-and-ATO-statement---JobKeeper-update/. Accessed 23 June 2021; Michelle Gratton, ‘Treasury Revises JobKeeper’s Cost Down by Massive $60 Billion, Sparking Calls to Widen Eligibility’ (*The Conversation*, Melbourne, 22 May 2020). https://theconversation.com/treasury-revises-jobkeepers-cost-down-by-massive-60-billion-sparking-calls-to-widen-eligibility-139231. Accessed 23 June 2021.

68 Australian Government, *Budget 2020–21: Economic Recovery Plan for Australia—COVID-19 Response—Supporting Australians through the Crisis* (Commonwealth of Australia 2020) 21.

69 Dan Conifer, ‘At Least $38b in JobKeeper Went to Companies Where Turnover Did Not Fall below Thresholds, Data Finds’ (ABC News, Sydney, 02 November 2021). www.abc.net.au/news/2021-11-02/38b-in-jobkeeper-went-to-companies-where-turnover-did-not-fall-/100586310. Accessed 05 November 2021.

70 Australian Bureau of Statistics, ‘One Year of COVID-19: Aussie Jobs, Business and the Economy’ (17 March 2021). www.abs.gov.au/articles/one-year-covid-19-aussie-jobs-business-and-economy. Accessed 23 June 2021; Stephanie Chalmers and Rachel Clayton, ‘Australia “Emerges from Recession” after GDP Figures Show Economy Growing for the First Time This Year’ (ABC News, Sydney, 02 December 2020). www.abc.net.au/news/2020-12-02/australia-september-quarter-economic-growth-gdp-figures/12934336. Accessed 23 June 2021.

71 Australian Bureau of Statistics, ‘One Year of COVID-19’ (n 70).

72 Swati Pandey, ‘Australian Economy Storms Ahead as COVID Recovery Turns “V-Shaped”’ (Reuters, New York, 03 March 2021). www.reuters.com/article/us-australia-economy-gdp-idUSKCN2AV02X. Accessed 23 June 2021.

73 Richard Holden, ‘Budget 2021: The Floppy-V-Shaped Recovery’ (*The Conversation*, Melbourne, 11 May 2021). https://theconversation.com/budget-2021-the-floppy-v-shaped-recovery-159230. Accessed 23 June 2021.

74 Australian Bureau of Statistics, ‘Media Release: Economic Activity Increased 1.8% in March Quarter’ (02 June 2021). https://www.abs.gov.au/media-centre/media-releases/economic-activity-increased-18-march-quarter. Accessed 23 June 2021; Michael Janda and Stephanie Chalmers, ‘Australia’s Economy 1.1 Per Cent Bigger Than at the Start of the COVID Pandemic, GDP Data Shows’ (ABC News, Sydney, 02 June 2021). www.abc.net.au/news/2021-06-02/gdp-march-quarter-economic-growth-covid-rebound/100184004. Accessed 23 June 2021.

75 Schmitt, *Political Theology* (n 1) 6.

76 Coronavirus Economic Response Package (Payments and Benefits) Act 2020 (Cth) s 21(1); emphasis added. An additional constitutional basis describes the JobKeeper Act as relying ‘on all other legislative powers that the Parliament has under the Constitution relevant to a kind of Coronavirus economic response payment’. Coronavirus Economic Response Package (Payments and Benefits) Act 2020 (Cth) s 21(2).

77 *Victoria v The Commonwealth and Hayden* (1975) 134 CLR 338.

78 *Australian Communist Party v The Commonwealth* (1951) 83 CLR 1.

79 *Victoria v The Commonwealth and Hayden* (n 77) 397.

80 Ibid.

81 *Australian Communist Party v The Commonwealth* (n 78) [25].

82 Nicholas Condylis, ‘Debating the Nature and Ambit of the Commonwealth’s Non-statutory Executive Power’ (2015) 39(2) *Melbourne University Law Review* 385, 392–393.

83 *Victoria v The Commonwealth and Hayden* (n 77) [19]; emphases added.

84 Ibid. at 362 (Barwick CJ), 375 (Gibbs J), 397 (Mason J), and 412 (Jacobs J); Peta Stephenson, ‘Nationhood and Section 61 of the Constitution’ (2018) 43(2) *University of Western Australia Law Review* 149, 151.

85 The expression ‘nationhood power’ was initially used by academics as a shorthand way of describing the power and was not adopted by a majority of the High Court until later decisions. Stephenson, ‘Nationhood and Section 61 of the Constitution’ (n 84) 151; Anne Twomey, ‘Post-*Williams* Expenditure: When Can the Commonwealth and States Spend Public Money without Parliamentary Authorisation?’ (2014) 33(1) *University of Queensland Law Journal* 9, 23.

86 *Davis v The Commonwealth* (1988) 166 CLR 79 [13].

87 Stephenson, ‘Nationhood and Section 61 of the Constitution’ (n 84) 153.

88 Anne Twomey, ‘Pushing the Boundaries of Executive Power: *Pape*, the Prerogative and Nationhood Powers’ (2010) 34(1) *Melbourne University Law Review* 313; Gabrielle Appleby and Stephen McDonald, ‘The Ramifications of *Pape v Federal Commissioner of Taxation* for the Spending Power and Legislative Powers of the Commonwealth’ (2011) 37(2) *Monash University Law Review* 162; Peter Gerangelos, ‘The Executive Power of the Commonwealth of Australia: Section 61 of the Commonwealth Constitution, “Nationhood” and the Future of the Prerogative’ (2012) 12(1) *Oxford University Commonwealth Law Journal* 97; Stephenson, ‘Nationhood and Section 61 of the Constitution’ (n 84) 149.

89 *Victoria v The Commonwealth and Hayden* (n 77) [20].

90 Stephenson, ‘Nationhood and Section 61 of the Constitution’ (n 84) 177; emphasis added.

91 *Pape v Commissioner of Taxation* (2009) 238 CLR 1.

92 Ibid. [8].

93 Pursuant to s 6 of the Tax Bonus for Working Australians Act (No. 2) 2009 (Cth).

94 *Pape v Commissioner of Taxation* (n 91) [133].

95 Ibid. [127].

96 Ibid. [133].

97 Ibid. [10].

98 Peter Gerangelos, ‘Reflections on the Executive Power of the Commonwealth: Recent Developments, Interpretational Methodology and Constitutional Symmetry’ (2018) 37(2) *University of Queensland Law Journal* 191, 203.

99 *Pape v Commissioner of Taxation* (n 91) [232].

100 Ibid. [233]; Twomey, ‘Pushing the Boundaries of Executive Power’ (n 88) 315.

101 Twomey, ‘Pushing the Boundaries of Executive Power’ (n 88) 315; Stephenson, ‘Nationhood and Section 61 of the Constitution’ (n 84) 162.

102 *Pape v Commissioner of Taxation* (n 91) [241]; emphasis added.

103 *Williams v The Commonwealth* (2012) 248 CLR 156.

104 The case concerned an agreement for funding between the Commonwealth Government and a Christian not-for-profit organisation, Scripture Union Queensland, to fund a national school chaplaincy programme, through which chaplains are placed in state schools and provide spiritual support to students and the school community.

105 *Williams v The Commonwealth* (n 103) [240].

106 Ibid. [196].

107 Ibid. [146] (per Gummow and Bell JJ), [499] (per Crennan J), [559] (per Kiefel J).

108 *CPCF v Minister for Immigration and Border Protection* (2015) 255 CLR 514.

109 Ibid. [150].

110 Gerangelos, ‘The Executive Power of the Commonwealth of Australia’ (n 88) 99; Condylis, ‘Debating the Nature and Ambit of the Commonwealth’s Non-statutory Executive Power’ (n 82) 388–389.

111 See Twomey, ‘Pushing the Boundaries of Executive Power’ (n 88); Twomey, ‘Post-*Williams* Expenditure’ (n 85); Stephenson, ‘Nationhood and Section 61 of the Constitution’ (n 84); Gerangelos, ‘Reflections on the Executive Power of the Commonwealth’ (n 98).

112 George Duke, however, points out that ‘[o]ne of the paradoxical features of the nationhood power’ is that the events in the context of which the power was developed, even events as significant as the GFC in *Pape*, are not ‘existential threats to the Australian polity’. See George Duke, ‘Popular Sovereignty and the Nationhood Power’ (2017) 45(3) *Federal Law Review* 415, 443.

113 Gerangelos, ‘Reflections on the Executive Power of the Commonwealth’ (n 98) 204.

114 Schmitt, *Political Theology* (n 1) 6–7.

115 Ibid. 12.

116 Ibid.

117 *Victoria v The Commonwealth and Hayden* (n 77) [19].

118 Schmitt, *Political Theology* (n 1) 12.

119 *Victoria v The Commonwealth and Hayden* (n 77) [21].

120 Ibid. See also Gerangelos, ‘Reflections on the Executive Power of the Commonwealth’ (n 98) 199; Twomey, ‘Pushing the Boundaries of Executive Power’ (n 88) 327.

121 Raymond CF Yiu, Chin-Pang Bennu Yiu, and Veronica QT Li, ‘Evaluating the WHO’s Framing and Crisis Management Strategy during the Early Stage of COVID-19 Outbreak’ (2021) 4(1) *Policy Design and Practice* 94, 96.

122 Parliament of Australia, ‘House of Representatives, Ministerial Statements—COVID-19’ (08 April 2020). https://www.aph.gov.au/Parliamentary_Business/Hansard/Hansard_Display?bid=chamber/hansardr/247e20e8-7bbe-4712-afcb-c8833dc6a228/&sid=0013. Accessed 23 June 2021; Prime Minister of Australia, ‘Transcript—Interview on Today’ (14 April 2020). www.pm.gov.au/media/interview-today-1. Accessed 23 June 2021.

123 Yiu et al., ‘Evaluating the WHO’s Framing and Crisis Management Strategy’ (n 121) 96.

124 Parliament of Australia, ‘House of Representatives, Ministerial Statements—COVID-19’ (08 April 2020) (n 122).

125 Schmitt, *Political Theology* (n 1) 6.

126 Ibid. 12.

127 Prime Minister of Australia, ‘Press Conference: Australian Parliament House’ (27 February 2020). www.pm.gov.au/media/press-conference-australian-parliament-house-4. Accessed 23 June 2021. The government’s response plan being the Australian Health Sector Emergency Response Plan for Novel Coronavirus (COVID-19).

128 The WHO characterised COVID-19 as a pandemic almost two weeks later. World Health Organization, ‘WHO Director-General’s Opening Remarks at the Media Briefing on COVID-19—11 March 2020’ (11 March 2020). www.who.int/director-general/speeches/detail/who-director-general-s-opening-remarks-at-the-media-briefing-on-covid-19---11-march-2020. Accessed 23 June 2021.

129 Prime Minister of Australia, ‘Press Conference: Australian Parliament House’ (27 February 2020) (n 127).

130 Dorothy Dixer is a term used to refer to a pre-arranged question asked of a government minister by a government backbencher, which creates an opportunity for the minister to highlight the government’s policies or achievements.

131 House of Representatives, *Commonwealth Parliamentary Debates*, 02 March 2020, 2140 (Scott Morrison); 03 March 2020, 2353 (Scott Morrison); 04 March 2020, 2544 (Scott Morrison); 05 March 2020, 2708 (Scott Morrison).

132 House of Representatives, *Ministerial Statements*, 23 March 2020, 2772 (Scott Morrison).

133 House of Representatives, *Commonwealth Parliamentary Debates*, 23 March 2020, 2777 (Josh Frydenberg).

134 House of Representatives, *Ministerial Statements*, 23 March 2020, 2774 (Scott Morrison).

135 House of Representatives, *Ministerial Statements*, 08 April 2020, 2909 (Scott Morrison).

136 Ibid. 2909–2910.

137 Paul Kelly, ‘Coronavirus: Nothing Will Challenge Australia’s Pre-crisis Values’ (*The Australian*, Surry Hills, 15 April 2020). https://www.theaustralian.com.au/commentary/coronavirus-nothing-will-challenge-australias-precrisis-values/news-story/843114fd4e062caf01049a24664eb4b2. Accessed 23 June 2021.

138 House of Representatives, *Ministerial Statements*, 08 April 2020, 2909 (Scott Morrison).

139 Ibid. Emphasis added.

140 Ibid. 2909–2910.

141 Explanatory Memorandum, Coronavirus Economic Response Package (Payments and Benefits) Bill 2020 (Cth) and Coronavirus Economic Response Package Omnibus (Measures No. 2) Bill 2020 (Cth) 4, 33, 35.

142 Ibid. 4, 9.

143 Spies-Butcher, ‘The Temporary Welfare State’ (n 54) 156.

144 House of Representatives, *Ministerial Statements*, 08 April 2020, 2909 (Scott Morrison).

145 Damien Cahill, ‘“Actually Existing Neoliberalism” and the Global Economic Crisis’ (2010) 20(3) *Labour & Industry: A Journal of the Social and Economic Relations of Work* 298, 301.

146 The Hon Josh Frydenberg MP, Treasurer of the Commonwealth of Australia, ‘COVID19: Australia’s Path to Recovery and Reform’, Address to the National Press Club (05 May 2020). https://ministers.treasury.gov.au/ministers/josh-frydenberg-2018/speeches/address-national-press-club. Accessed 23 June 2021.

147 House of Representatives, *Ministerial Statements*, 08 April 2020, 2914 (Anthony Albanese); Peter Van Onselen, ‘Coronavirus: “Ideological Constraints” Are Out the Window, Where They Belong’ (*The Australian*, Surry Hills, 11 April 2020) 20. https://www.theaustralian.com.au/inquirer/coronavirus-ideological-constraints-are-out-the-window-where-they-belong/news-story/88c4673ff4a7752b7b0ec329af6466cd. Accessed 23 June 2021; Carol Johnson, ‘Has Coronavirus Killed Ideology? No, It’s Just Cycled It Around Again’ (*The Conversation*, Melbourne, 19 April 2020). https://theconversation.com/has-coronavirus-killed-ideology-no-its-just-cycled-it-around-again-136615. Accessed 23 June 2021.

148 House of Representatives, *Ministerial Statements*, 08 April 2020, 2909 (Scott Morrison).

149 Prime Minister of Australia, ‘Speech: Address, Victorian Chamber of Commerce and Industry’ (10 November 2021). www.pm.gov.au/media/address-victorian-chamber-commerce-and-industry. Accessed 11 November 2021.

150 Ibid. Emphasis added. See also Simon Benson and Joe Kelly, ‘PM Backs “Can-Do” Capitalism in Election Shift’ (*The Australian*, Surry Hills, 10 November 2021). https://www.theaustralian.com.au/nation/scott-morrison-backs-cando-capitalism/news-story/705371b71385112319b6c34cc9ca636c. Accessed 11 November 2021.

151 Editorial, ‘The Prime Minister’s Phrase Book Has Election Written All over It’ (*The Age*, Melbourne, 11 November 2021). www.theage.com.au/politics/federal/the-prime-minister-s-phrase-book-has-election-written-all-over-it-20211110-p597nk.html. Accessed 11 November 2021.

152 Schmitt, *Political Theology* (n 1) 12.

153 Coronavirus Economic Response Package (Payments and Benefits) Act 2020 (Cth) s 3.

154 Australian Government, ‘Fact Sheet: JobKeeper Payment: Supporting Businesses to Retain Jobs’ (25 April 2020). https://treasury.gov.au/sites/default/files/2020-04/Fact_sheet_supporting_businesses_0.pdf. Accessed 23 June 2021.

155 House of Representatives, *Ministerial Statements*, 08 April 2020, 2909 (Scott Morrison); House of Representatives, *Commonwealth Parliamentary Debates*, 08 April 2020, 2918 (Josh Frydenberg).

156 Explanatory Memorandum, Coronavirus Economic Response Package (Payments and Benefits) Bill 2020 (Cth) and Coronavirus Economic Response Package Omnibus (Measures No. 2) Bill 2020 (Cth) 34.

157 House of Representatives, *Commonwealth Parliamentary Debates*, 08 April 2020, 2918 (Josh Frydenberg).

158 Spies-Butcher, ‘The Temporary Welfare State’ (n 54) 155.

159 Explanatory Memorandum, Coronavirus Economic Response Package (Payments and Benefits) Bill 2020 (Cth) and Coronavirus Economic Response Package Omnibus (Measures No. 2) Bill 2020 (Cth) 4.

160 Ibid. 34.

161 Sarah Kaine, ‘Australian Industrial Relations and COVID-19’ (2020) 85 *Journal of Australian Political Economy* 130, 132.

162 Prime Minister of Australia, ‘Transcript—Address, National Press Club’ (26 May 2020). www.pm.gov.au/media/address-national-press-club-260520. Accessed 23 June 2021.

163 John Quiggin, ‘Crises and Recession as the Norm’ (2020) 85 *Journal of Australian Political Economy* 39, 40.

164 House of Representatives, *Commonwealth Parliamentary Debates*, 08 April 2020, 2918 (Josh Frydenberg).

165 Coronavirus Economic Response Package (Payments and Benefits) Act 2020 (Cth) ss 3, 7(1); Explanatory Memorandum, Coronavirus Economic Response Package (Payments and Benefits) Bill 2020 (Cth) and Coronavirus Economic Response Package Omnibus (Measures No. 2) Bill 2020 (Cth) 33–35; Australian Government, ‘Fact Sheet: JobKeeper Payment: Supporting Businesses to Retain Jobs’ (25 April 2020) (n 147); House of Representatives, *Commonwealth Parliamentary Debates*, 08 April 2020, 2919 (Josh Frydenberg).

166 Explanatory Statement, Coronavirus Economic Response Package (Payments and Benefits) Amendment Rules (No. 8) 2020 (Cth) 1.

167 The Hon Josh Frydenberg MP, Treasurer of the Commonwealth of Australia, ‘Media Release: JobKeeper Update’ (30 November 2020). https://ministers.treasury.gov.au/ministers/josh-frydenberg-2018/media-releases/jobkeeper-update-0. Accessed 23 June 2021; Australian Government: The Treasury, *The JobKeeper Payment: Three Month Review* (TSY/AU June 2020) 7.

168 Ian Verrender, ‘How JobKeeper Turned into Profit Maker’ (ABC News, Sydney, 22 March 2021). www.abc.net.au/news/2021-03-22/how-jobkeeper-turned-into-profit-maker-ian-verrender/100020236. Accessed 23 June 2021.

169 Australian Government: The Treasury, *The JobKeeper Payment: Three Month Review* (TSY/AU June 2020) 36.

170 Gareth Hutchens, ‘ATO Has Not Investigated Companies Paying Executive Bonuses while Receiving JobKeeper’ (ABC News, Sydney, 12 February 2021). www.abc.net.au/news/2021-02-12/ato-and-jobkeeper/13145990. Accessed 23 June 2021; Ben Butler, ‘Auditor General to Investigate JobKeeper after It Was Used to Pay Dividends and Bonuses’ (*The Guardian*, Sydney, 02 February 2021). www.theguardian.com/australia-news/2021/feb/03/auditor-general-to-probe-jobkeeper-after-it-was-used-to-pay-dividends-and-executive-bonuses. Accessed 23 June 2021.

171 The Auditor General’s report was due to be tabled in March 2022. Australian National Audit Office, ‘Performance Audit—Administration of the JobKeeper Scheme’ (2021). www.anao.gov.au/work/performance-audit/administration-the-jobkeeper-scheme. Accessed 05 November 2021; Butler, ‘Auditor General to Investigate JobKeeper’ (n 170).

172 Senate, *Official Committee Hansard*, 11 February 2021 (Senate Select Committee on Covid-19) 12; Hutchens, ‘ATO Has Not Investigated Companies Paying Executive Bonuses’ (n 170).

173 Nikitha Kariyawasam and James Samson, ‘Update on JobKeeper & Other Government Subsidies in ASX300’ (Ownership Matters, 2021).

174 Ibid. 1.

175 Madeleine Morris, ‘Shareholders Reap Millions from Top Companies Pocketing JobKeeper, New Analysis Finds’ (ABC News, Sydney, 26 March 2021). www.abc.net.au/news/2021-03-26/top-companies-pocketing-jobkeeper-new-analysis-finds/100030274. Accessed 23 June 2021.

176 Eagers Automotive Limited, *Annual Report 2020* (Eagers Automotive Ltd 2020).

177 A shadow minister is a member of the opposition party with responsibility for closely scrutinising the performance of the government with respect to a particular ministry. The shadow minister would ostensibly assume the relevant ministerial position in the event of a change of government, and (if a member of the shadow cabinet) contributes to the development of opposition policy.

178 Ben Butler, ‘JobKeeper Doubled Return for Investors Compared with Companies That Did Not Have Subsidy’ (*The Guardian*, Sydney, 14 April 2021). www.theguardian.com/business/2021/apr/15/jobkeeper-doubled-return-for-investors-compared-to-companies-that-did-not-have-subsidy. Accessed 23 June 2021.

179 Paul Karp, ‘Australia’s Biggest Companies Pocketed Hundreds of Millions in JobKeeper despite Positive Earnings’ (*The Guardian*, Sydney, 17 March 2021). www.theguardian.com/australia-news/2021/mar/18/australias-biggest-companies-pocketed-hundreds-of-millions-in-jobkeeper-despite-positive-earnings. Accessed 23 June 2021; Dominic Powell, ‘“Hit by a Rainbow”: Fears Millions Wasted on JobKeeper Payments to Profitable Companies’ (*The Sydney Morning Herald*, Sydney, 18 March 2021). www.smh.com.au/business/companies/hit-by-a-rainbow-fears-millions-wasted-on-jobkeeper-payments-to-profitable-companies-20210317-p57blx.html. Accessed 23 June 2021; Ben Butler and AAP, ‘Harvey Norman to Keep $22m in JobKeeper despite Profits Doubling to $462m in Pandemic’ (*The Guardian*, Sydney, 26 February 2021). www.theguardian.com/australia-news/2021/feb/26/harvey-norman-to-keep-22m-in-jobkeeper-despite-profits-doubling-to-462m-in-pandemic. Accessed 23 June 2021; Ben Butler, ‘Car Dealer AP Eagers to Give Shareholders $64m in Dividends after Receiving $130m in JobKeeper Subsidies’ (*The Guardian*, Sydney, 24 February 2021). www.theguardian.com/australia-news/2021/feb/24/car-dealer-ap-eagers-to-give-shareholders-64m-in-dividends-after-receiving-130m-in-jobkeeper-subsidies. Accessed 23 June 2021.

180 Reported in the media but not publicly available.

181 Conifer, ‘At Least $38b in JobKeeper Went to Companies Where Turnover Did Not Fall Below Thresholds’ (n 69).

182 Nassim Khadem, ‘JobKeeper and Early Super Release Rorts and Overpayments on the Rise’ (ABC News, Sydney, 12 March 2021). www.abc.net.au/news/2021-03-12/ato-owed-millions-jobkeeper-rorts-and-overpayments-early-super/13231222. Accessed 23 June 2021.

183 Dominic Powell, ‘“Not a Good Look”: Push Grows for JobKeeper Payments to Be Returned as Profits, Dividends Boom’ (*The Sydney Morning Herald*, Sydney, 02 March 2021). www.smh.com.au/business/companies/not-a-good-look-push-grows-for-jobkeeper-payments-to-be-returned-as-profits-dividends-boom-20210302-p576zn.html. Accessed 23 June 2021; Simon Evans, ‘Eagers Automotive Won’t Repay Any of $130m in JobKeeper’ (*The Australian Financial Review*, Sydney, 24 February 2021). www.afr.com/companies/transport/eagers-automotive-won-t-repay-any-of-130m-in-jobkeeper-20210223-p57553. Accessed 23 June 2021.

184 Prime Minister of Australia, ‘Transcript: Q&A, National Press Club—Barton, ACT’ (01 February 2021). www.pm.gov.au/media/qa-national-press-club-barton-act. Accessed 23 June 2021.

185 Conifer, ‘At Least $38b in JobKeeper Went to Companies Where Turnover Did Not Fall Below Thresholds’ (n 69).

186 Australian Government: The Treasury, *Insights from the First Six Months of JobKeeper* (Commonwealth of Australia 2021) 1; Steven Hamilton and Richard Holden, ‘ALP Takes the Low Road on JobKeeper’ (*The Australian*, Sydney, 23 September 2021). www.theaustralian.com.au/commentary/alp-takes-the-low-road-on-jobkeeper/news-story/e2d6fb0add94544954a7f73b0fff4e2a. Accessed 05 November 2021.

187 Verrender, ‘How JobKeeper Turned into Profit Maker’ (n 168); Kariyawasam and Samson, ‘Update on JobKeeper & Other Government Subsidies in ASX300’ (n 173) 11.

188 Parliamentary Budget Office, ‘Policy Costing: Profitable Corporations to Return JobKeeper Payments’ (22 April 2021). www.aph.gov.au/-/media/05_About_Parliament/54_Parliamentary_Depts/548_Parliamentary_Budget_Office/Costings/Publicly_released_costings/2021/Profitable_corporations_to_return_JobKeeper_payments_PDF.pdf?la=en&hash=8FBF72C42CCAF0FD8395F5C74CC4697AC04F7D5A. Accessed 23 June 2021. See also Andrew Brown, ‘Greens’ JobKeeper Payback Scheme Could Reap More Than $1 Billion’ (*Canberra Times*, Canberra, 03 May 2021). www.canberratimes.com.au/story/7234502/push-for-businesses-to-pay-back-jobkeeper-funds-could-reap-1-billion/. Accessed 23 June 2021.

189 Coronavirus Economic Response Package Amendment (Ending JobKeeper Profiteering) Bill 2021 (Cth); Senate, *Commonwealth Parliamentary Debates*, 21 June 2021, 3319 (Nicholas McKim).

190 Coronavirus Economic Response Package Amendment (Ending JobKeeper Profiteering) Bill 2021 (Cth) s 19A. See also John Buckley, ‘Greens JobKeeper Repayment Bill Surfaces in the Senate’ (*Accountants Daily*, 24 June 2021). www.accountantsdaily.com.au/tax-compliance/15843-greens-jobkeeper-repayment-bill-surfaces-in-the-senate. Accessed 23 September 2021.

191 Coronavirus Economic Response Package Amendment (Ending JobKeeper Profiteering) Bill 2021 (Cth) s 19A(3).

192 Ibid. s 19B.

193 The Committee not being obliged to accept every document it received as a submission, it is unknown whether other submissions were made to the Committee and not accepted or not published.

194 Parliament of Australia, ‘Submissions’. https://www.aph.gov.au/Parliamentary_Business/Committees/Senate/Economics/TLABjobkeeperprofiteering/Submissions. Accessed 26 July 2021. Submissions were received from Ownership Matters Pty Ltd, Australian Council of Trade Unions, Australasian Centre for Corporate Responsibility, Australian Shareholders Association Limited, Domino’s Pizza Enterprises, Professor Rabee Tourky, Professor Rohan Pitchford, and Toyota Motor Corporation Australia Ltd.

195 Domino’s Pizza Enterprises Ltd, ‘Submission to the Economics Legislation Committee—Inquiry into Coronavirus Economic Response Package Amendment (Ending JobKeeper Profiteering) Bill 2021’ (Senate Economics Legislation Committee 2021). www.aph.gov.au/Parliamentary_Business/Committees/Senate/Economics/TLABjobkeeperprofiteering/Submissions. Accessed 26 July 2021.

196 As in the case of Toyota Motor Corporation Australia Ltd. See Toyota Motor Corporation Australia Ltd, ‘Submission to the Economics Legislation Committee—Inquiry into Coronavirus Economic Response Package Amendment (Ending JobKeeper Profiteering) Bill 2021’ (16 July 2021). www.aph.gov.au/Parliamentary_Business/Committees/Senate/Economics/TLABjobkeeperprofiteering/Submissions. Accessed 26 July 2021.

197 Rabee Tourky and Rohan Pitchford, ‘Submission to the Economics Legislation Committee—Inquiry into Coronavirus Economic Response Package Amendment (Ending JobKeeper Profiteering) Bill 2021’ (16 July 2021). www.aph.gov.au/Parliamentary_Business/Committees/Senate/Economics/TLABjobkeeperprofiteering/Submissions. Accessed 26 July 2021.

198 Senate Economics Legislation Committee, *Report: Coronavirus Economic Response Package Amendment (Ending JobKeeper Profiteering) Bill 2021* (Commonwealth of Australia, October 2021) [2.73].

199 Ibid. [2.75].

200 Ibid. [2.74].

201 For example, the New Zealand Government provided a publicly accessible ‘COVID-19 Wage Subsidies—Employer Search’ function via the website of its Ministry of Social Development. New Zealand Government, Ministry of Social Development, ‘COVID-19 Wage Subsidies—Employer Search’ (2021). www.msd.govt.nz/about-msd-and-our-work/newsroom/2020/covid-19/covid-19-wage-subsidy-employer-search.html. Accessed 20 September 2021. Whereas the Government of the United Kingdom published data monthly starting from December 2020 regarding employers which had claimed through the UK’s Coronavirus Job Retention Scheme. See Government of the United Kingdom—HM Revenue & Customs, ‘Transparency Data: Employers Who Have Claimed through the Coronavirus Job Retention Scheme’ (2021). www.gov.uk/government/publications/employers-who-have-claimed-through-the-coronavirus-job-retention-scheme. Accessed 20 September 2021.

202 Schmitt, *Political Theology* (n 1) 13.

203 Schupmann, *Carl Schmitt’s State and Constitutional Theory* (n 28) 180; Kivotidis, ‘“Norm” and “Exception”’ (n 34) 122–125; Schmitt, *Constitutional Theory* (n 32) 169.

204 Schmitt, *Political Theology* (n 1) 12–13.

205 Agamben, *The Omnibus Homo Sacer* (n 9) 196–197.

206 Kivotidis, ‘“Norm” and “Exception”’ (n 34) 119.

207 Ong, *Neoliberalism as Exception* (n 45) 5; emphases added.

208 House of Representatives, *Ministerial Statements*, 08 April 2020, 2909 (Scott Morrison).

209 Kean Birch, *We Have Never Been Neoliberal: A Manifesto for a Doomed Youth* (Zero Books 2015) 132–134.

210 Morris, ‘Shareholders Reap Millions’ (n 175).

211 Lorraine Talbot ‘Why Shareholders Shouldn’t Vote: A Marxist-Progressive Critique of Shareholder Empowerment’ (2013) 76(5) *Modern Law Review* 791, 806.

212 Paul Karp, ‘Harvey Norman Repays $6m of the $22m It Claimed in JobKeeper after Record Profits’ (*The Guardian*, Sydney, 31 August 2021). www.theguardian.com/australia-news/2021/aug/31/harvey-norman-repays-6m-of-the-22m-it-claimed-in-jobkeeper-after-record-profits. Accessed 23 September 2021.

213 Gareth Hutchens, ‘Here Are the 20 Companies on the ASX300 That Have Pledged to Return JobKeeper Payments—Was Public Pressure the Key?’ (ABC News, Sydney, 14 July 2021). www.abc.net.au/news/2021-07-14/jobkeeper-repaid-comes-from-public-companies/100288376. Accessed 27 September 2021.

214 E.g., see Domino’s Pizza Enterprises Ltd, ‘Submission to the Economics Legislation Committee’ (n 195); Robert Gottliebsen, ‘The Trouble with the “Return JobKeeper” Push’ (*The Australian*, Surrey Hills, 27 September 2021). www.theaustralian.com.au/business/economics/the-trouble-with-the-return-jobkeeper-push/news-story/fd4ceb2268cea1dd4c90a841504b8748. Accessed 27 September 2021.

215 Talbot ‘Why Shareholders Shouldn’t Vote’ (n 211) 806.

216 Ibid. See also Birch, *We Have Never Been Neoliberal* (n 209) 182.

