# Supplementary material for: COVID, crisis, and unordinary order: A critical analysis of Australia’s JobKeeper wage subsidy scheme as an exceptional measure
Source: Jindal Global Law Review. 2022 Jun 2;13(1):39–68. doi: 10.1007/s41020-022-00166-9 (PMC9160508; doi:10.1007/s41020-022-00166-9)
Supplement: Supplementary file 1 — Supplementary file1 (PDF 5423 kb) [file 41020_2022_166_MOESM1_ESM.pdf]

ABC NEWS

# Australia 'emerges from recession' after GDP figures show economy growing for the first time this year

By business reporters Stephanie Chalmers and Rachel Clayton

Posted Wed 2 Dec 2020 at 10:35am, updated Wed 2 Dec 2020 at 7:41pm

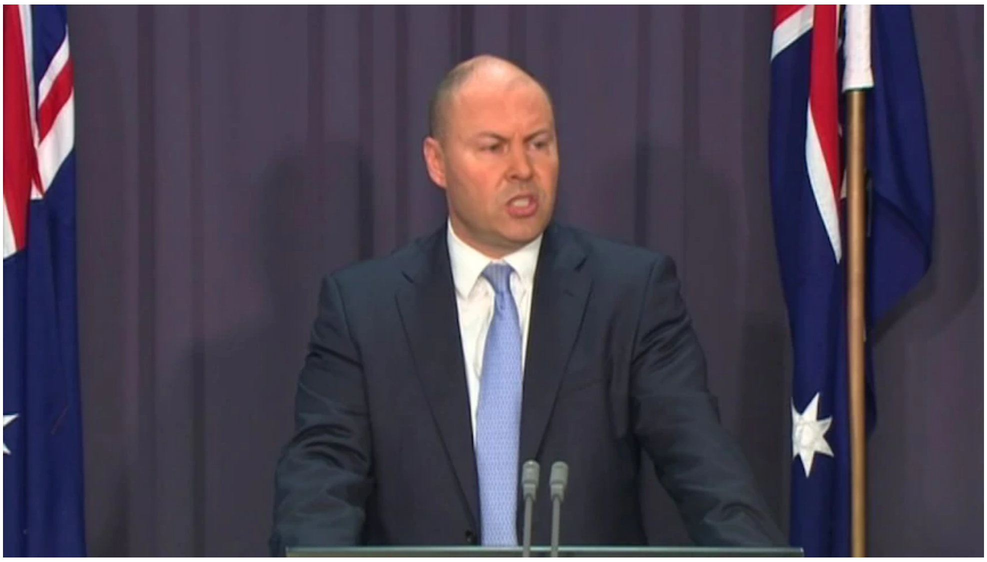

The Government has announced the GDP figures for the September quarter.

The Reserve Bank governor is warning recovery from this year's recession is likely to be "uneven and drawn out" as Australia's gross domestic product (GDP) grows for the first time in 2020.

**Key points:**

- GDP rose 3.3 per cent this quarter, as COVID-19 restrictions eased across most states and territories
- Household consumption grew almost 8 per cent after a 12.5 per cent decline last quarter
- Reserve Bank governor Philip Lowe expects the economy to continue growing this quarter but Australia's recovery will be "uneven and drawn out"

The latest figures from the Bureau of Statistics show GDP grew 3.3 per cent in the September quarter, but declined 3.8 per cent in the year to September 2020.

Alexander Hell is the director of the 35-year-old Melbourne company Westside Talent, which provides entertainment acts for venues, weddings and festivals.

He said the pandemic led to his revenue source disappearing.

"It was something you couldn't prepare for," he said.

Mr Hell said JobKeeper, other Government support and his own personal savings helped keep the business afloat, but he's worried that's all going to change when JobKeeper is reduced again.

"The work has not picked up at all in any great abundance. There's skerricks coming through and little things coming through, but nothing that I could say is going to compensate [for that].

"There's certain industries, and ours being one of those industries, that are going to struggle to get back on their feet.

"It's entertainment, it's where people mingle, it's social — that's what our business is based on."

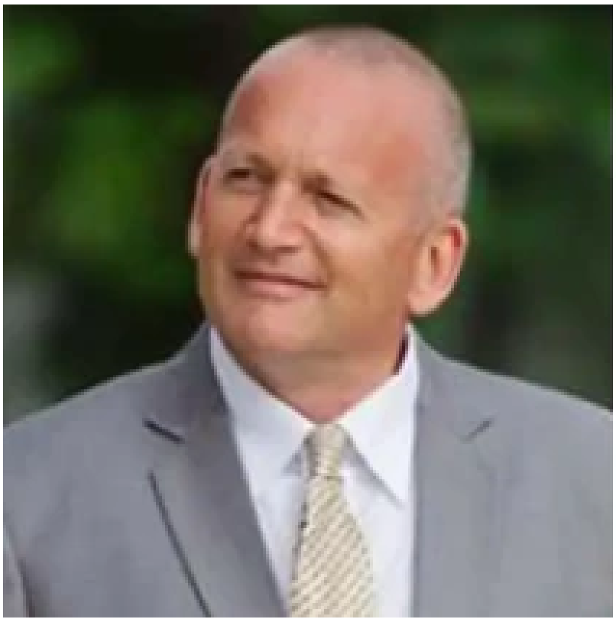

Alexander Hell says things remain tough for his talent agency. *(Supplied: Westside Talent)*

## 'Technically the recession is over'

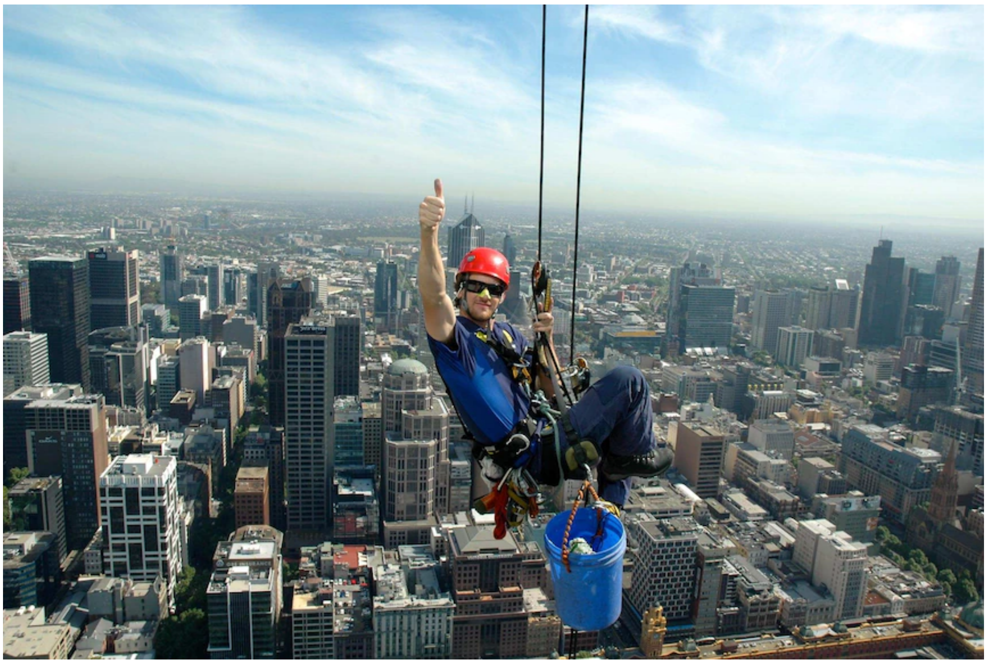

The easing of COVID-19 restrictions has allowed large parts of Australia's economy to reopen. *(Supplied: Jarrad Church)*

The September quarter growth came after a 7 per cent economic contraction in the three months through June — the worst fall on record, which confirmed Australia had entered a technical recession due to the coronavirus pandemic.

The return to growth follows a year of economic contraction, but with high unemployment forecast to persist for several years, the effects of the recession continue to be felt.

CommSec chief economist Craig James said the growth meant Australia was technically out of recession.

The textbook definition of a recession is two consecutive quarters of economic contraction, but Mr James said that definition was not perfect and failed to take into account "the societal impact of recession … especially on the labour market".

"But based on this definition, Australia has emerged from recession and the outlook for growth is encouraging," he said.

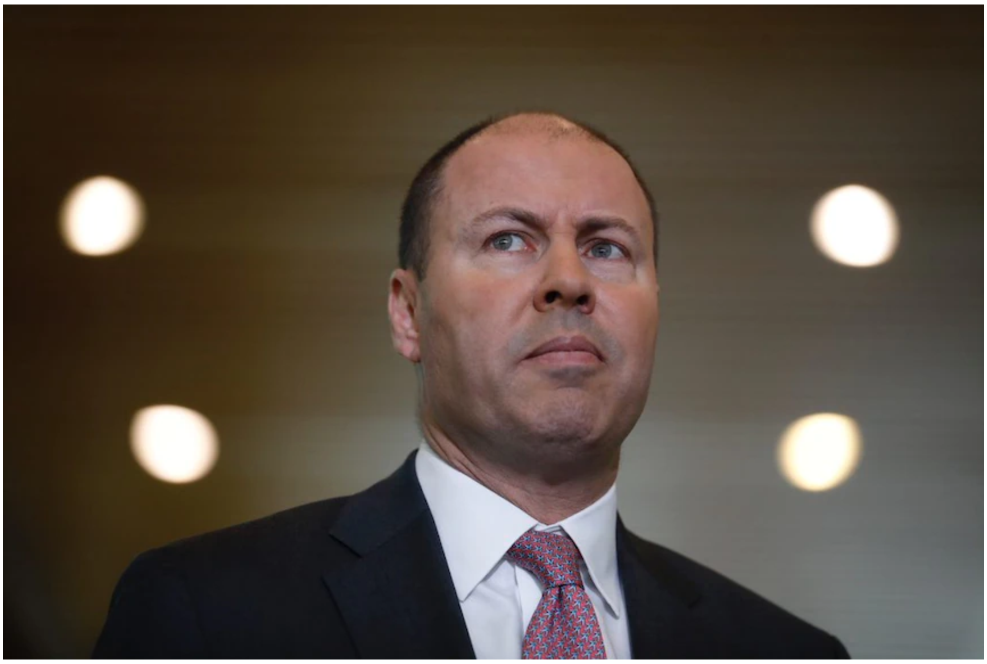

Josh Frydenberg says the recession may be over, but the recovery will take time. *(ABC News: Matt Roberts)*

Federal Treasurer Josh Frydenberg said there was reason to take hope in the latest numbers, but said Australia was "not out of this crisis yet".

"Technically the recession is over, but the recovery is not," he said.

"The economic indicators are positive. That being said, this is a very challenging time and there's a lot of ground to make up."

## 'Recovery will be uneven and it will be bumpy'

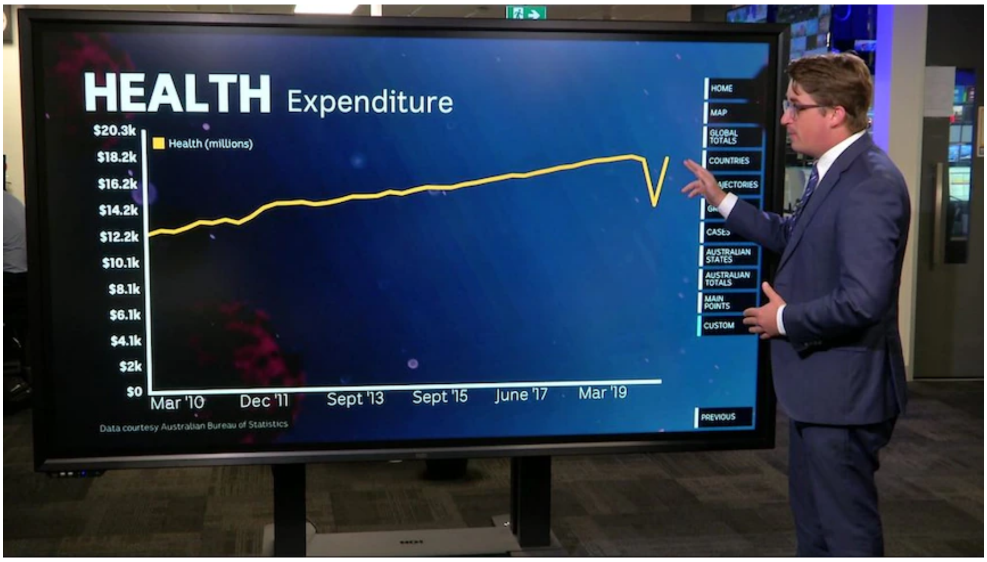


ABC analyst Casey Briggs looks at the latest spending figures.

Speaking in Canberra ahead of the figures being released, Reserve Bank governor Philip Lowe said he expected positive GDP growth in the current quarter as well.

"Given these developments, we are now expecting GDP growth to be solidly positive in both the September and December quarters. And then, next year, our central scenario is for the economy to grow by 5 per cent and then 4 per cent over 2022," Dr Lowe said.

"These positive figures, though, cannot hide the reality that the recovery will be uneven and it will be bumpy and it will be drawn out. Some parts of the economy are doing quite well, but others are in considerable difficulty.

"Australia is likely to experience a run of years of relatively high unemployment, unemployment being too high and wage increases and inflation being too low, leaving us short of the Reserve Bank's goals."

Dr Lowe added that: "Even with the overall economy now growing solidly, it will not be until the end of 2021 that we again reach the level of output recorded at the end of 2019."

Household consumption, which makes up almost 60 per cent of Australia's economy, grew by 7.9 per cent in the September quarter, the largest rise in the 60-year history of the national accounts.

The growth reflects a partial recovery from the 12.5 per cent fall in the June quarter.

The Bureau of Statistics noted the rise reflected an increase in spending as COVID-19 restrictions were eased.

However, household consumption remained weak through the year, down 6.5 per cent on the September 2019 quarter.

## Some start to spend as recovery begins

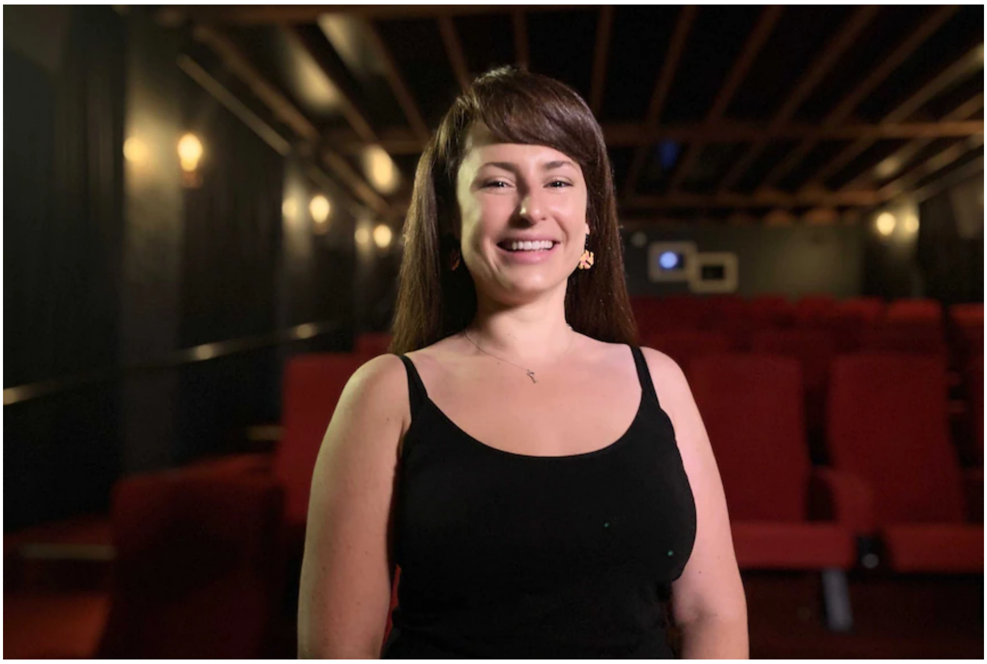

Jessica Litsas and her husband had their incomes reduced as the Victorian second wave shut their cinema. *(ABC News: Michael Barnett)*

Jessica Litsas is the co-owner and director of the Pivotonian Cinema in Geelong and has begun spending as the economy starts to recover.

Jessica and her husband normally work full-time in the business, so with the cinema shut during Victoria's second wave of COVID-19, both their incomes were reduced to the JobKeeper rate.

"JobKeeper was quite confronting because we had to re-evaluate all of our family income and spending," she said.

In late September, as the Pivotonian Cinema remained shut due to ongoing restrictions in Victoria, the JobKeeper rate was reduced.

"When it dropped from $1,500 a fortnight to $1,200 a fortnight, our family budget decreased by $600, and that's a lot of money," Jessica said.

> "That's not only groceries, bills but childcare — we had to apply for temporary financial relief to meet our childcare payments."

But with the cinema now reopen and customer limits gradually being relaxed, Jessica and her husband are drawing a wage again and feeling more optimistic.

"I'm going to live it up! I'm very excited to be going out to restaurants again and I'm going to the zoo with my daughter on Friday up in Melbourne," she said.

"I'm very happy to be on a full wage at work again and I'm going to take advantage of that."

She's not alone, hotels, pubs and restaurants saw the biggest increase in spending, up almost 50 per cent after a 56.3 per cent decline in the previous quarter.

Victoria was the only state or territory to record a decline in household spending (-1.2 pc).

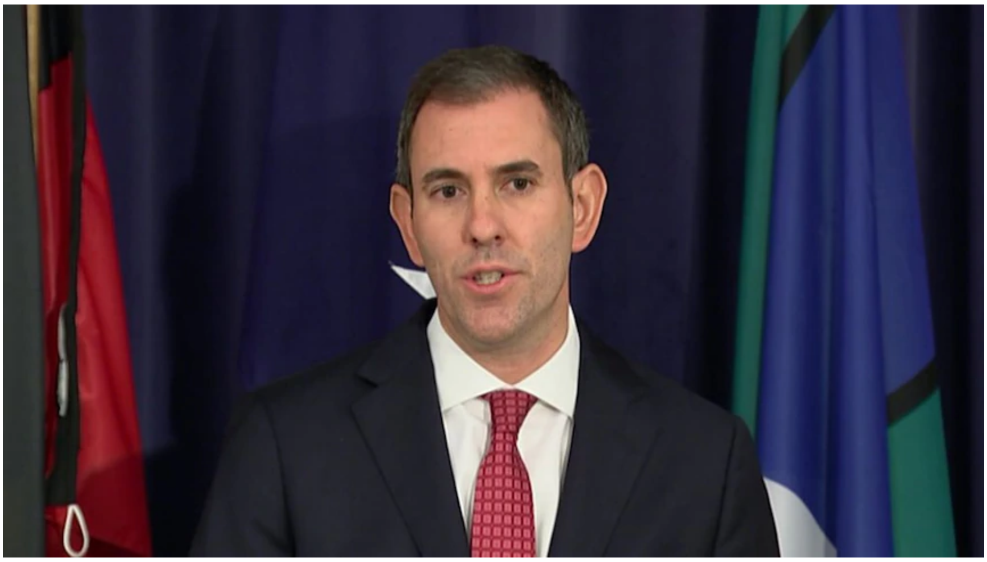

Shadow Treasurer Jim Chalmers says the Federal Government hasn't done enough to boost wages and living standards.

Sarah Hunter, chief economist for BIS Oxford Economics, said Victoria's continued decline in spending reflected the impact of the state's second lockdown.

"This confirms that Victoria's economy should rebound sharply in the December quarter given the easing of restrictions and suppression of COVID," Dr Hunter said.

"As expected business investment declined further — 3 per cent on the quarter.

"The ongoing impact of the pandemic on trade is also clear, with services exports falling back a further 8.8 per cent as a result of lower total international student enrolments."

Dr Hunter predicted "robust growth" in the December quarter as Victoria begins to catch up with the rest of the country.

She said the Federal Government's monetary support for households and residential construction was likely to boost consumer spending.

"But we remain somewhat cautious on the outlook for trade and business investment. Overall, we expect GDP to return to pre-COVID levels towards the end of 2021."

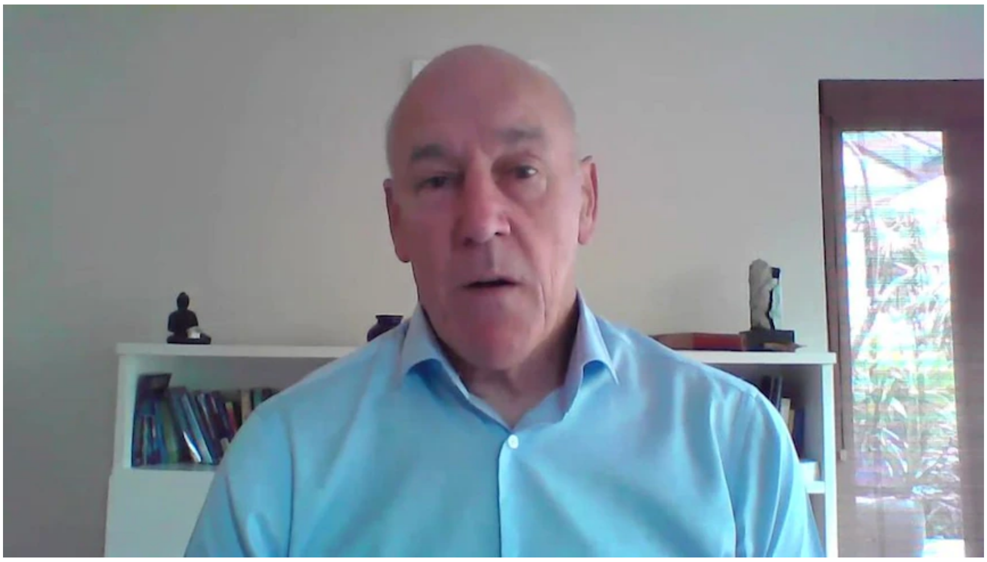

ABC Business editor Ian Verrender says the GDP figures should be kept in perspective.
*(ABC News)*

## 'Cautiously optimistic' about 2021

But as Dr Lowe predicted, the recovery has been uneven across the country.

Deb Garske is the owner and designer of a guesthouse in Byron Bay, NSW that was shut for months during the pandemic.

She said the popular tourist town changed within a couple of days when staff returned to their homes overseas and visitors stopped coming.

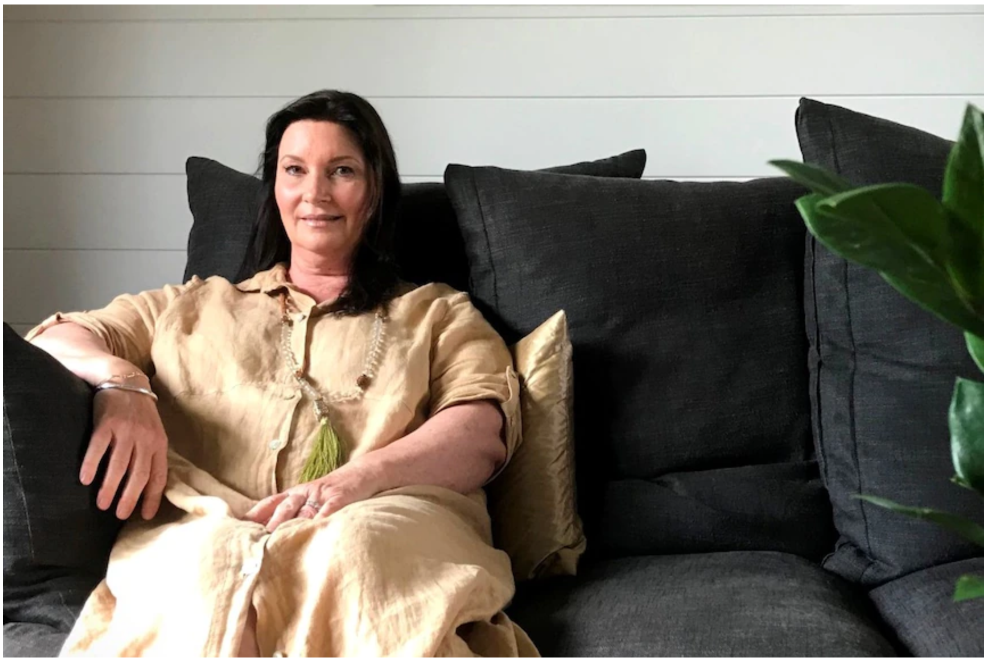

Deb Garske's 28 Degrees guesthouse in Byron Bay has seen an influx of domestic tourists.
*(ABC News: Leah White)*

Lately, her guesthouse has been attracting domestic tourists who would normally holiday overseas, and she's seen a boost from travel agents recommending Australian travel options instead.

"For us, we're doing really well and we're so happy that we're getting so many repeat guests coming back, that's the one thing that's really changed."

Ms Garske said she was "cautiously optimistic" about the future.

"We just have to go cautiously, we can't expect that everyone's going to come up here, we don't know what's going to happen in our winter. We're looking at the UK and Europe and they're struggling, and America.

> "I won't be spending any money this year and next year, I think I just want to stay tight and work on improving the business as far as accommodation goes."
