# Supplementary material for: COVID, crisis, and unordinary order: A critical analysis of Australia’s JobKeeper wage subsidy scheme as an exceptional measure
Source: Jindal Global Law Review. 2022 Jun 2;13(1):39–68. doi: 10.1007/s41020-022-00166-9 (PMC9160508; doi:10.1007/s41020-022-00166-9)
Supplement: Supplementary file 2 — Supplementary file2 (PDF 1979 kb) [file 41020_2022_166_MOESM2_ESM.pdf]

ABC NEWS

Australia's economy 1.1 per cent bigger than at the start of the COVID pandemic, GDP data shows

By business reporters Michael Janda and Stephanie Chalmers

Posted Wed 2 Jun 2021 at 11:43am, updated Wed 14 Jul 2021 at 11:31am

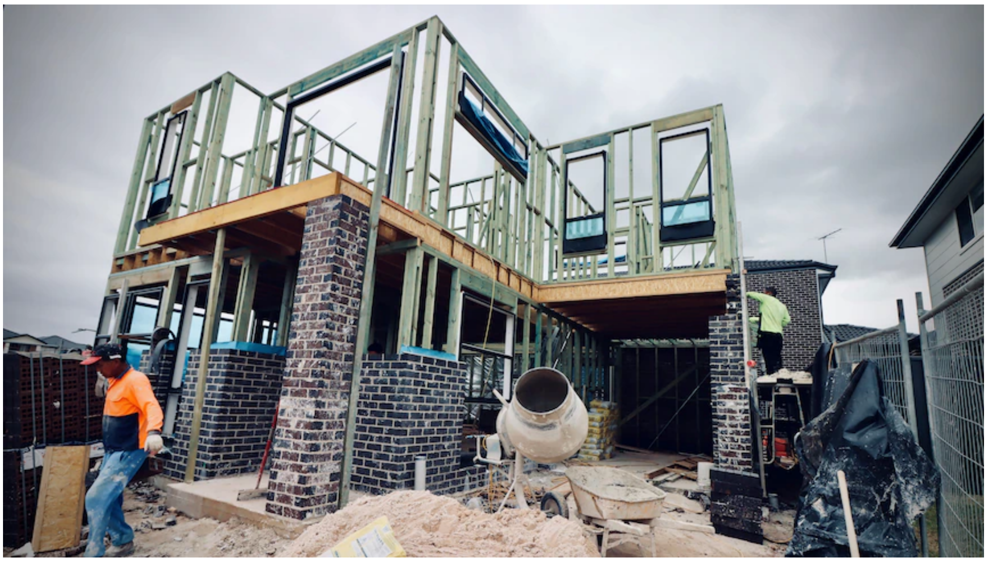

Dwelling construction jumped 6.4 per cent as HomeBuilder boosted renovations and new builds. *(ABC News: John Gunn)*

Australia's economy has bounced back from the COVID recession, growing by a much-better-than-expected 1.1 per cent over the past year.

Key points:

- GDP grew 1.8 per cent in the March quarter, above economist expectations of 1.6 per cent
- The Australian economy is now 0.8 per cent bigger than its previous record, prior to the bushfire crisis and pandemic
- Both business and household investment and spending contributed to the result

As recently as a few weeks ago, most economists were hoping that Australia's economy might just get back to where it was before nationwide pandemic lockdowns from late March to May last year.

But not only is Australia's economic output bigger than it was before the national lockdown, it also recovered from the previous quarter's bushfire and early pandemic-driven decline, and is 0.8 per cent larger than its previous peak.

The experience of Melbourne florist Shane Sipolis over the first few months of the year is typical of the rebound, as people and businesses started to regain confidence to book events.

Melbourne florist Shane Sipolis is dealing with his fourth COVID lockdown. *(ABC News: Peter Drought)*

"After weeks of normality, we kicked back off again and a lot of our weddings that had been backlogged from the year before ... started to really pick up," he said.

With the events sector playing catch-up, it was even busier than pre-pandemic.

"We even had one weekend where we had to try and get eight weddings done," he said.

> "A lot of them had actually more money to spend — they were coming back to me and asking to increase the floral budget."

Australia comes out of COVID smelling roses

Deloitte Access Economics said there were only five other reasonably comparable countries that could boast an economy bigger than it was prior to COVID-19.

Australia's economy had one of the quickest and strongest recoveries from the COVID pandemic so far. *(Supplied: Deloitte)*

The annual result was powered by a much-better-than-forecast 1.8 per cent expansion over the three months to March, when most economists were expecting 1.6 per cent.

Even those forecasts had been substantially revised higher as recent ABS data that feed directly into the GDP number came in well above expectations.

Private sector takes over from public

Analysts are also pleased with the main drivers of growth, split between private investment (which added 0.9 percentage points to the quarterly result) and household consumption (which added 0.7 percentage points).

Deloitte Access Economics partner Kristian Kolding said the GDP figures showed Australia's recovery was becoming more broad-based.

"Families are spending locally, and businesses continue to invest, making the most of record-low interest rates and tax offsets," he observed.

"Meanwhile, government stimulus is becoming a much smaller driver of growth than it was last year."

The ABS said private investment was driven by both business machinery and equipment purchases, up 11.6 per cent in the strongest result since December 2009, and housing investment, up 6.4 per cent.

In turn, both of those areas were supported by government subsidies — temporary full expensing of business investment and the HomeBuilder scheme.

Commonwealth Bank economist Kristina Clifton said a return to more normal levels of "going out" led the rise in consumer spending.

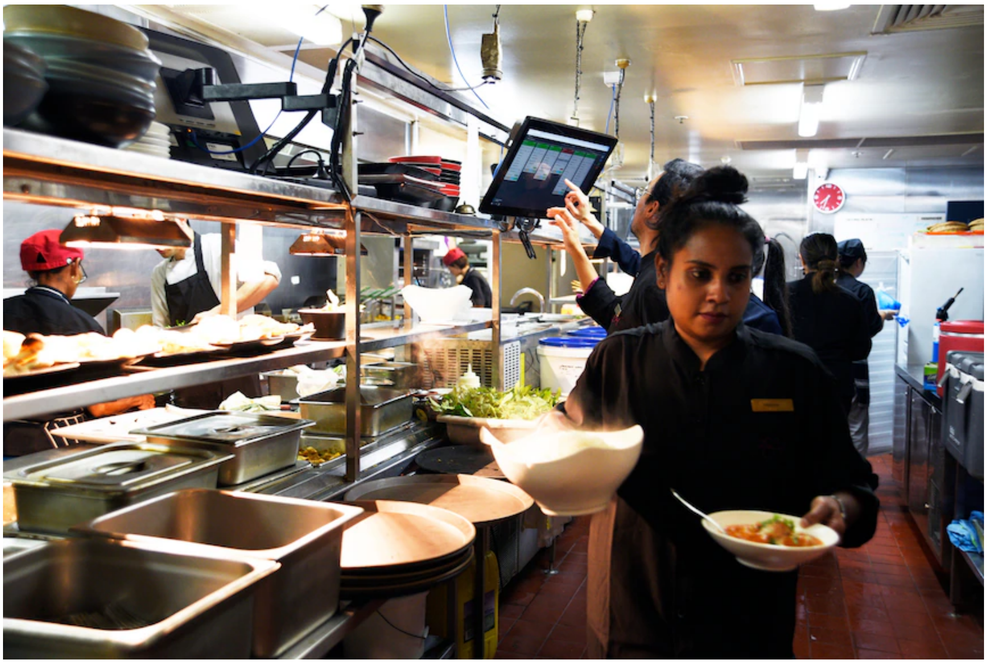

The GDP figures showed a dramatic 14.8 per cent jump in spending in hotels, cafes and restaurants. *(ABC News: Roxanne Fitzgerald)*

"The recovery in services spending continued in the quarter," she noted.

"There was a 14.8 per cent lift in spending in hotels, cafes and restaurants as well as a 3.3 per cent lift in recreation and culture.

"There has been a lift in domestic tourism as borders largely remained opened during the first quarter. Transport services also rose, lifting by 8.8 per cent."

But as people started going out more, they did trim their grocery spending.

"Spending on goods fell by 0.5 per cent in the quarter, driven by falls in food (-1.4 per cent) and alcohol goods (-3.9 per cent) as spending on eating and drinking outside the home lifted as restrictions eased."

National COVID 'second wave' threat

However, most economists also caution that Australia cannot rest on its achievements so far.

CommSec's chief economist Craig James pointed out that Victoria's current lockdown — the effects of which will not show up in the GDP data until the June quarter figures are released in September — is a warning that the economic recovery could be quickly undone.

"Stimulus must remain in place until it is clear that a sustainable recovery has been achieved," he wrote.

"Measures to suppress the virus need to be reinforced. And vaccination rates need to accelerate.

> "The primary threats to the economic recovery are a broad Australian 'second wave' of the virus and a slow, extended vaccine rollout."

In Melbourne, Shane Sipolis has just spent the weekend trying to resell flowers he had already bought for corporate events that were cancelled.

But he found there was a sense of fatigue among people who had rallied behind local businesses during previous lockdowns.

That meant thousands of dollars worth of flowers ended up in the bin.

"We were all starting to get sort of settled in and thinking like we could start to return to some sort of normal," he said.

> "This is the fourth lockdown, and it being a snap lockdown, there is a huge fear that anything can change at any moment, so that really rocks people's confidence."

The federal government again indicated on Wednesday that it had no intention to revive its JobKeeper wage subsidy scheme in response to state lockdowns.

Mr Sipolis said he was unlikely to hire more staff while COVID uncertainty persists, even to cope with the busy periods like the weekends with multiple weddings he experienced earlier this year.

"When we employ somebody, then we feel responsible for them and if everything switches at any moment, then that's also quite a difficult position to be in," he added.
