# Supplementary material for: COVID, crisis, and unordinary order: A critical analysis of Australia’s JobKeeper wage subsidy scheme as an exceptional measure
Source: Jindal Global Law Review. 2022 Jun 2;13(1):39–68. doi: 10.1007/s41020-022-00166-9 (PMC9160508; doi:10.1007/s41020-022-00166-9)
Supplement: Supplementary file 3 — Supplementary file3 (PDF 397 kb) [file 41020_2022_166_MOESM3_ESM.pdf]

COMMONWEALTH OF AUSTRALIA

# Official Committee Hansard

# SENATE

## SENATE SELECT COMMITTEE ON COVID-19

**Australian Government's response to the COVID-19 pandemic**

THURSDAY, 11 FEBRUARY 2021

CANBERRA

BY AUTHORITY OF THE SENATE

**INTERNET**

Hansard transcripts of public hearings are made available on the internet when authorised by the committee.

To search the parliamentary database, go to:
**http://parlinfo.aph.gov.au**

# SENATE

## SENATE SELECT COMMITTEE ON COVID-19

### Thursday, 11 February 2021

**Members in attendance:** Senators Davey [by video link], Gallagher, Lambie [by video link], Paterson, Siewert [by video link].

**Terms of Reference for the Inquiry:**

To inquire into and report on:

On 8 April 2020 the Senate established the Select Committee on COVID-19 and referred the following matters to it for inquiry and report on or before 30 June 2022:

a. the Australian Government's response to the COVID-19 pandemic; and

b. any related matters.

The resolution establishing the committee is available in the Journals of the Senate No. 48 - Wednesday, 8 April 2020.

## WITNESSES


**FISH, Ms Kirsten, Acting Second Commissioner, Law Design and Practice, Australian Taxation Office ... 11**

**HIRSCHHORN, Mr Jeremy, Second Commissioner, Client Engagement Group, Australian Taxation Office ... 11**

**KENNEDY, Dr Steven, PSM, Secretary, Treasury ... 1**

**O'HALLORAN, Mr James, Deputy Commissioner, Economic Stimulus Branch, Australian Taxation Office [by video link] ... 11**

**POWER, Mr Trevor, First Assistant Secretary, Macroeconomic Conditions Division, Treasury ... 11**

**PRESTON, Mr Robb, Assistant Secretary, Retirement Income Policy Division, Treasury ... 11**

**WILKINSON, Ms Jennifer, Deputy Secretary, Fiscal Group, Treasury ... 1**

**WILKINSON, Ms Jennifer, Deputy Secretary, Fiscal Group, Treasury ... 11**

**YEAMAN, Mr Luke, Deputy Secretary, Macroeconomic Group, Treasury ... 1**

**YEAMAN, Mr Luke, Deputy Secretary, Macroeconomic Group, Treasury ... 11**

**KENNEDY, Dr Steven, PSM, Secretary, Treasury**

**WILKINSON, Ms Jennifer, Deputy Secretary, Fiscal Group, Treasury**

**YEAMAN, Mr Luke, Deputy Secretary, Macroeconomic Group, Treasury**

**Committee met at 11:00**

**CHAIR (Senator Gallagher):** I declare open this hearing of the Senate Select Committee on COVID-19. Today's public hearing will focus on the Australian government's economic response to COVID-19 but may cover other matters under the terms of reference. Information on the procedural rules governing public hearings has been provided to all witnesses and is available from the secretariat. If a witness objects to answering a question, the witness should explain the basis for the objection in sufficient detail to allow the committee to determine whether to accept the objection. The committee will then decide whether to insist on an answer. Witnesses may request that answers be given confidentially.

I now welcome officers of the Department of the Treasury. Dr Kennedy, I understand you're not making a formal opening statement, but I would invite you to make some remarks before members of the committee ask questions.

**Dr Kennedy:** Thank you, Chair. First, I have some general comments about how we're seeing the economic outlook. Maybe, if I make a few remarks about the global outlook and then the domestic outlook, that might be helpful. I will also say a little bit about how we see the policy transition.

In very general terms, the economy is recovering faster than we anticipated late last year, when MYEFO was put out. The Reserve Bank were in a number of appearances last week and outlined their updated forecasts for the unemployment rate and a number of other economic aggregates. We would, in broad terms, concur with the direction, which is a positive direction domestically. Partly, perhaps, that's about the measures being more effective than we anticipated, but it's also, of course, because the health outcomes remain relatively good in Australia. The pandemic has been well managed, and that's very clear when one looks around the world.

Turning to the rest of the world, despite the fact that the pandemic is clearly having a larger effect on other countries, there is a sense in which growth prospects are being revised up for some countries, particularly for the US. China has been growing strongly. How things are unfolding in Europe is less clear, but I think people are taking confidence from the rollout of the vaccination programs. We've now seen, according to news reports, more people in the world having received vaccination shots than there have been cases—more than 100 million now around the world. There is a strong sense that the vaccination program being run out is giving people confidence, but of course there are still many people dying around the world and the disease is still spreading rapidly. While uncertainty is being resolved, there is still considerable uncertainty about the outlook.

The domestic outlook will, of course, continue to depend heavily on how well the health side of things is measured, and I would have to say that all governments across Australia have done an excellent job in the way the pandemic has been measured. There are very difficult trade-offs to make in how to change behaviours, including with the so-called shutdowns to manage the pandemic. They're difficult decisions for all governments across Australia, but clearly the outcomes are good in Australia. Those trade-offs are always hard to get right, but one would have to say, from the perspective of cases and even, sadly, deaths in Australia and of the economic impact, that Australia has done really quite well. People's minds are moving to how well we will recover. It is a year now, really, since things really took off, and peoples' minds are moving to that.

With that, on the domestic outlook, consumption is recovering very strongly, very much supported by measures that the Commonwealth government has put in place. Consumer confidence is returning to either above or around pre-COVID levels. As we expected in MYEFO, we think this is a consumption-led recovery in the sense of consumption will recover first. Balance sheets, household balance sheets, in particular, and business balance sheets, which I'll come to, are well placed. People have actually been retiring their debt through this period. Partly that may have been because of their precautionary behaviour but also because the things they may have wished to spend their money on, they couldn't, because of the restrictions—for example, international travel, all those sorts of things.

It does mean, if confidence can remain high, particularly around the management of the health side of the pandemic, then we would expect people to continue to consume at least at the levels they're at in the period ahead and run some of that buffer down. A lot of that does depend on confidence and maintaining that confidence on the health side.

On the investment side, there's been a very strong demand for detached housing, very much supported by the HomeBuilder program. The HomeBuilder program, particularly in December, has encouraged a significant

increase in approvals for houses. It's less strong in medium density. Non-residential construction is also weak. We will be looking for business investment in the next phase, as the recovery unfolds across the course of this year and into next year, as an important part of where things kick in. The government's measures around loss carry-back and immediate expenses and others will be important there. Public investment is high and expected to continue to increase. The states have made now around $120 billion worth of fiscal decisions over the course of the last year or so, which I very much welcome. Many of those measures will impact in the year ahead and beyond. Many of those are infrastructure measures. They will be important to see this transition from the immediate recovery through to the medium term and longer recovery.

On the labour market side, the labour market is recovering surprisingly well. I have to say I'm very pleased and surprised to see participation back at record levels, at what it was pre-COVID. That is good. The unemployment rate is at 6.6 per cent and falling more rapidly than we expected. At MYEFO, we expected the unemployment rate to peak at seven to 7½ in March. That looks unlikely to me now. The RBA outlined their forecast last week, which saw the unemployment rate falling from here. That does seem more likely to me, but of course the government's forecasts will be updated in budget. That is very good news.

Inside the labour market, there are some differences. Employment levels for people aged over 35 are back above pre-COVID levels, which is a very positive sign. For people aged between 15 and 34, employment levels are still two or three per cent below what they were pre-COVID. That has very much unfolded in the way we expected. People have gone back to jobs and those jobs that are being generated are going to more experienced people. The hiring credit will be an important policy in this area that targets that 15- to 34-year-old group. Always, coming out of any downturn, the young, who won't be competing for jobs with experience and coming into a softer labour market, may need some support and hence the training programs and other things. Despite this general widespread strength, there are some particular variations by sector and region.

Clearly sectors like aviation are badly affected by the restrictions and the reimposing of restrictions on the internal travel arrangements. On international travel arrangements it's hard for us to predict, even when international returns, whether it will return strongly. What will be very important, I think—and this is what I'm told from the aviation sector—is maintaining as much domestic travel as possible. The profitability of airlines in Australia very much depends on their capacity to fly to Brisbane, Melbourne, Sydney and those types of routes. The capacity to keep the health situation well under control and predictable in the way any changes are made to those situations is crucial for that sector and for arts and recreation and any service sector that's affected by restrictions around how many people can gather and those types of things. In education and training, there's that aspect of international education in particular.

For things like tourism in the broad sense and cafes and restaurants, it's very much a mixed picture. Some areas are telling us they've had very strong growth and enjoyed a very strong period over Christmas. Others were badly affected by immediate shutdowns and had to move. There's quite a mixed picture there, but there's no doubt that, in aggregate, for example, cafes and restaurants are affected. That's not surprising given the restrictions around the capacity for people to gather.

There will be a very important period in the year ahead with the vaccination rollout not only for its obvious health benefits and broader benefits to the community but for the importance of it being rolled out in a way through which the community retains its confidence around how the government is managing the current health situation. That is very important for confidence around the economic situation.

Some of the measures, which no doubt you would like to talk about today, are scheduled to come off. Some will come off and leave money behind in balance sheets, such as JobKeeper. The government obviously still has decisions in front of it around COVID supplements and other things that it's foreshadowed. HomeBuilder will taper off. But then most of the activity that's been generated by HomeBuilder will actually occur in the latter half of this year and into the following year.

New measures will take effect this year. The LMTO payment will happen around July when people are putting in their tax returns. They will also get additional money in their tax return from having had LMTO apply this year. So consumption will get a boost then. The business investment supports always take a lag to take their full effect for lost carry-back and a range of other things. There are been some other important measures. There's the job training and hiring credit, as I mentioned. Perhaps I'll leave it there. Chair, is that enough for you?

**CHAIR:** It is. I can see why you're the head of Treasury. That was a very comprehensive briefing. Thank you. Can we just talk a bit about what's going to happen at the end of March. You touched on it in your opening comments. We've got JobKeeper ending on the 26th and then JobSeeker is ending on the 31st. What needs to happen in April to smooth the transition away from these payments, if they are going to end? What needs to fill that gap?

**Dr Kennedy:** On JobKeeper, I think the payments are made in April, so there's not much difference to what you're outlining. It will end, and people will be anticipating its scheduled end. What we saw between JobKeeper 1 and JobKeeper 2 was a move from around 3.6 million people down to about 1.6 million people covered by JobKeeper 2. The next move will be different. We expect to see fewer in March, but the next move will be different because we're not moving to a lower level; it's ending. I'd expect it will mean there's some peoples' employment that won't be present and job losses that would come of that, as was the case between the move from JobKeeper 1 to JobKeeper 2. In the move from JobKeeper 1 to JobKeeper 2, in a sense those job losses in aggregate were overwhelmed by the amount of jobs being created on the other side. For this next transition, from March through to June, rather than an overwhelming offset we will more likely see roughly what looks like a bit of a pause in the labour market. I note that the RBA have the unemployment rate flat as it hits that period through the first half of this year. So that would be my sense. In terms of how it would turn up in the labour market itself, we have seen so much employment growth that it would be hard to see employment growing at the high levels it has, because a lot of that is bringing back—

**CHAIR:** What was lost, yes.

**Dr Kennedy:** So you'll see more of the move than you would otherwise have seen in the first move, because it was getting overwhelmed by other changes in the background. I don't expect it to disturb the trajectory of the unemployment rate coming down and more employment being generated across the course of the year, for the reasons the other measures are in place. The fact is that many businesses are—to use the language that is often used with me—well cashed up and able to invest, and if they have the confidence they will invest. We haven't seen business credit move yet, but that's partly because there's so much liquidity in the system. That's not everywhere; as I said, there are specific areas that will be more difficult—particularly aviation, because they're affected directly by restrictions with people not travelling. It's much more complicated in other areas because people have changed their businesses where they can and have adapted across the course of the year.

In terms of sectors the effects become narrower. Unlike any other downturn in Australia's recent history, the areas that are most affected by sheer dint of numbers in Australia are probably the Melbourne and Sydney CBDs, with people not travelling into the cities or going to cafes and restaurants. Once again, a lot depends on how all governments manage the health side of it, the transition back to work and people travelling in the cities again and using those restaurants.

In our forecast we had an expectation there would be outbreaks—so we put our MYEFO forecast together on the basis of there being outbreaks. As I said, things have been stronger since then. My sense is that the current fiscal setting, the very substantial Commonwealth money supported by the state moneys, and the way it switches in its emphasis, is about right. But we will have to watch it very closely. I should add that Australia would have a fiscal response in the order of 18 per cent of GDP. That's a very substantial physical response, with 12 per cent on the Commonwealth side and about six per cent on the state side.

**Senator PATERSON:** How does that compare internationally?

**Dr Kennedy:** In terms of legislated programs, it would be close to the highest in the developed world. New Zealand would be around 14 or 15 per cent of GDP. The package being proposed in the US by the Biden administration is very substantial and would go beyond what I'm talking about, but it has to be legislated. Canada is extending its package; it's higher than the UK package. So the governments have come together here in a very substantial way. I think the quantum is right. Of course all governments should watch very carefully both the delivery of that fiscal package and its composition. It's a substantial package.

**CHAIR:** Just following on from that: almost all the economic commentators have warned against withdrawing fiscal support too soon, and I think the government has made some sounds about ongoing targeted support. You've got 1.5 million people still on JobKeeper and 1.3 million people on the elevated JobSeeker. That's a lot of Australians—nearly three million people. You said some jobs will be lost, and the RBA governor has said the same thing. How many will be lost, and what's going to happen to those three million people when these payments are wound back?

**Dr Kennedy:** I don't think there will be anything like millions of jobs being lost.

**CHAIR:** Do you have an idea? Surely the government has wanted to know, before they take the decision to end it, what's going to happen to the people on it.

**Dr Kennedy:** I might pass to Ms Wilkinson in a moment. It's very hard to predict exactly how many will go. We do look at people on zero hours or very low hours in firms, because they're obviously people who have been furloughed, in a sense; held with no activity. That would be a worker that, if firms were no longer being subsidised to pay for that worker, they would possibly be looking to put off or come to some other arrangement.

Of course, in terms of their own financial support, it's not a good thing if that happens, but they would go on to JobSeeker, of course, because the net exists there.

Just in terms of this language of fiscal support being withdrawn, the current package was designed to come in and go out in the way it does. Its impact on economic growth lingers, we think, for at least two to three years because of the sheer size of it and the way that it helps the balance sheet and helps people draw down on a balance sheet, and also because of what we had done in the crisis phase. Effectively the government had come along and closed activities through health measures. The government had then also borrowed from the future and replaced that loss of income. That's partly what's happened with some of these measures, and JobKeeper is a big part of that. That was entirely appropriate. That part of it—if the broader economy is not shut down that way—should, I think, come out. Then it's a question of trying to calibrate the broader fiscal incentive to ensure that you've got your growth. Most countries' wage subsidy programs are ending around the time our is, or have ended earlier. New Zealand's ended in September. The only one I know of that's continuing across the course of the next year is the German one, but it has been dramatically changed in its shape. So the time for those measures, I think, is passing.

Your completely reasonable question about how much fiscal support remains in the system to drive growth beyond that, is a good question. We think there's certainly enough to drive the recovery. As I said, it's going better than what we'd thought—but we'll continue to advise the government. This is a really important question actually, Senator. Just on a structural point, which the RBA governor would have made, monetary policy continues to be accommodative, but the capacity to support any shorter-term shocks really now leans much more heavily on fiscal policy than ever before. That is partly why your committee is so interested in how the recovery proceeds month-to-month. That's the approach we are taking, keeping the government fully abreast. We're quietly confident now that the recovery is locked in. But the health situation—things can turn so dramatically that you've got to watch it incredibly closely.

**CHAIR:** Do we need to keep a high level of JobSeeker going, in your opinion?

**Dr Kennedy:** There are decisions around JobSeeker in front of the government now. So I'll let the government make those decisions. The decisions around JobKeeper predominately—

**CHAIR:** The governor of the bank had a view on it.

**Dr Kennedy:** Yes. I have a view too and the government hears what that view is. Once they've made their decision, then I'd be in a position to talk about it and describe it. I think it's a reasonable courtesy for me to allow the government to make its view on what that is and for me to advise them through the appropriate cabinet processes. The point I would make about JobSeeker in this next phase—it's somewhat related to JobKeeper—is that it's much less about its macroeconomic circumstances now, and it's much more about the decision that the government needs to make in light of the adequacy of the payment, how it intersects with incentives to work. And I think that's all I'd say on that at this stage.

**CHAIR:** There'll be an announcement soon on that, I presume? It's only 45 days away from being reduced back to $40 a day. So I hope for the—

**Dr Kennedy:** Unfortunately in these committees I don't get too lean to the minister and say, 'Do you want to take that, Minister'? But I expect the government will—

**CHAIR:** No. I had to ask. You choose how you respond. You referenced LMITO and that fact that that's coming in the second half of this year. Was there any lift in consumer spending after the previous LMITO? Were you able to measure that?

**Dr Kennedy:** We talked about that not long after I took this position. The trick in measuring the lift was always that you never knew what you had before you put it in there. What people talked about when the last LMITO came in was that consumption didn't strengthen by as much as they anticipated in the second half of that year, which was a reasonable comment to make. The complication was we never knew what consumption. And we never will know what consumption was without it. I guess the question is: do you think a LMITO has an impact on consumption? There's no doubt in my mind that it does. People were commentating that it didn't look as large as what we thought it might, but, frankly, underlying consumption might've been weaker. I'd expect it to have a positive impact on consumption when it comes in, particularly because of the groups that would get LMITO.

**CHAIR:** So you have no way really of measuring it?

**Dr Kennedy:** We draw on the economic literature to think about what fiscal multipliers look like. In advising the government about where a fiscal multiplier is likely to be larger we would say that money to support spending

will always be larger when given to low-income groups—hence the COVID supplement and hence future support, effectively through the LMITO double payment.

**CHAIR:** When consumer spending is getting measured in the models that you use as you are putting together packages, you must attribute a component of that to LMITO. Is that right?

**Dr Kennedy:** We do, yes.

**Mr Yeaman:** As we discussed previously with the committee, we do have a broad view in our mind, and our models support this, of the key factors that drive consumption but those are at an aggregate level. We know that income drives consumption. We know that wealth drives consumption, whether it's house prices or stock prices, so we try and look through that. There's enough movement in those variables that it's very hard to pick out the individual impact of discrete policy measures for us. I think at the time that that measure was implemented in the past, there were other things happening in terms of house prices. There were other issues playing into consumer confidence at that time. They are very hard for us to look through and get a detailed point estimate of how that would impact consumption.

**CHAIR:** We might get back to that when the secretary has left and you guys all get to stay. I'm stretching the friendship of the committee here, but in the Statement on Monetary Policy that was released on Friday there's a graph which goes consolidated government finances. It's really a question around the fiscal consolidation. When you look at that graph, what it looks like to me is—and I understand it is intense fiscal support and it was targeted, temporary and all the rest of it; I get that background—that there is a very sharp contraction or consolidation over a two-year period. Basically you are trying to wind it back by five per cent of GDP over three years—about three and 3½ per cent. You are looking at a fiscal consolidation in the order of 10 per cent of GDP over three years. That's not something that has been achieved past previous recessions, where the pain out has taken a lot longer. What makes you think that this sort of fiscal consolidation can occur without having consequential negative impacts on the economic recovery?

**Dr Kennedy:** Partly it's the nature of this shock. Normally coming out of a recovery some form of the economy has been disrupted in the shock in the first instance, like the credit channel was broken because banks got ahead of themselves or capital was misallocated. In the nineties that may have been complicated by policy mistakes, and in that case there was a very slow response on fiscal policy. We've seen this in the early stages—all the productive capital that was in place, the banking system and all those sorts of things are all there on the other side of this. The shock has not so much disrupted the economic channels. If we've been effective—and of course we'll have to watch; you've got to approach unprecedented events with some humility in terms of understanding how they will unfold, because they're unprecedented and I have no previous experience to draw on in terms of recovering from a pandemic—our expectation is that, once restrictions have come off, economic activity can resume quite rapidly, particularly if you've been able to maintain whole many of the businesses through that period, which I think we have from the very large restrictions. And people are keen, in a sense, and confident to get back to work. That's partly where the vaccination strategy comes in. So I am hopeful, but we will watch very closely, that we'll have a faster private economic impulse than what a normal economic recovery would have because of the nature of the shock that we got in the first instance. A crucial key to that to watch over the next year or two is business investment.

**CHAIR:** I was going to go to that, but I was running out of time.

**Dr Kennedy:** Sorry I've taken up too much of it.

**CHAIR:** No.

**Dr Kennedy:** That's where the immediate expensing and loss carry back is important. We're really trying to set an incentive for business to crystallise business investment in this next year and a half and then to support that subsequent recovery. The bank's forecast in the statement you just spoke about and our forecasts all incorporate the nature of the fiscal response, and, in both our views, the recovery takes hold—and relatively rapidly compared to past recoveries. But, as I said, we all want to approach these circumstances with a bit of humility. These are unprecedented times.

**CHAIR:** I've taken up enough time. Senator Paterson.

**Senator PATERSON:** Thank you Dr Kennedy for your time today. There are lots of things we could revisit from your opening remarks; I won't have time to do it all. But one comment you made that I was particularly struck by was the end of New Zealand's wage subsidy scheme back in September. I think I saw the other day that their unemployment rate is at 4.9 per cent or thereabouts. Clearly the withdrawal of a wage subsidy scheme for them has not caused millions of people to be thrown into the unemployment scrap heap. Does that give you some confidence that the sky won't fall when JobKeeper comes to an end in Australia?

**Dr Kennedy:** It does. It's partly related to the capacity for—because we've got variations in our own country—states, like New Zealand, to have health under control and to be able to release restrictions. That really gives you confidence that that activity will unfold and that even where there are small outbreaks, they're well managed in the first instance and, secondly, they're predictably managed, because that allows business to plan around them. They can have an idea about what will happen if an outbreak comes; they won't be caught out. I think we've all learnt a lot through this exercise. To see what Victoria was able to achieve in getting back on top of its outbreak was really a remarkable outcome. Another remarkable outcome was to see a city of more than four million people, Sydney, get on top of an outbreak in such a targeted way. That must be, I hope, giving the community confidence about the capacity of the states to respond and then, more and more, hopefully tailor that response. The whole country can benefit from that. Some states, such as WA, are ahead of where they were pre-COVID.

**Senator PATERSON:** Obviously Australia's and New Zealand's health performance has not been exactly the same, but they're broadly comparable.

**Dr Kennedy:** They are.

**Senator PATERSON:** They've had the occasional hotel quarantine outbreak too. They seem to have a good contact-tracing system, as we now largely do.

**Dr Kennedy:** They do.

**Senator PATERSON:** So there is something to be learned from that. I remember in one of your earlier appearances before the committee, I think in the early stages of the JobKeeper program, we talked about why it would be necessary for JobKeeper to come to an end at some point. There are the obvious fiscal reasons behind that—if want to run a balanced budget again, we can't continue to subsidise a big chunk of the private-sector workforce forever. But beyond that I remember you talking about things like labour-market dynamism and making sure that workers were going to industries that had good prospects and that were growing. Is there any evidence of why turning that dynamism back on again remains important?

**Dr Kennedy:** That would be our view, and it sits behind our assessments around advice, if you like, on the ending of JobKeeper. JobKeeper was performing more roles than simply the normal wage subsidy role—encouraging employment. It was really a unique type of wage subsidy arrangement. It was trying to keep businesses whole when they were not able to operate, and it was doing that by linking employees of the firm to that firm. That was good through that period. But subsequent to that, that link and that capacity for people to move—and we've seen some of that move anyway, I think it's fair to say—we've seen a lot of JobKeeper firms employing new employees and coming back on. As an aggregate measure, these unintended consequences steadily become more pronounced over time. I particularly think that's the case a year out.

Very badly affected sectors—such as aviation, which I spoke about earlier, and maybe a couple of others, narrowly defined—have a persistent problem, which the government's giving some consideration to. But as a macroeconomic tool, it's losing its—from my perspective, frankly, it's time to think about other types of tools to encourage activity. The hiring credit is a good example of a different tool that's saying to firms: 'Here's a person who's been on JobSeeker, a young person. Very fortunately there are fewer people going on JobSeeker than we thought, so that's fantastic. I'm going to give you an incentive to employ them and retain them, as long as they're additional to your existing workforce.' It's about, 'Here's an incentive for you to invest.' I think it has to move to a growth mindset rather than a hold-the-firm-whole mindset.

**Senator PATERSON:** Yes, there are obviously good theoretical reasons to believe this might be a problem. I'm also aware of lots of anecdotal evidence of some employers saying that they are finding it difficult to get workers. There are some industries, such as agriculture, in particular, that have very demonstrable problems. Beyond that, is there any good data yet that fleshes this out? Or are we just in a bit of an uncertain space here and we just have to make assumptions?

**Dr Kennedy:** On—

**Senator PATERSON:** On labour availability—the cafe owner who tells me, 'I advertise and I can't get someone to come and work.' There'd be other things that contribute to that, like the fact that there are no international students and no temporary visa holders.

**Dr Kennedy:** At the aggregate level, if we think back through the data, we're back at record participation. That's a lot of people who are back. And the unemployment rate is higher than it was, at 6.6 per cent. But that's a lot of people back in the workforce and wanting to contribute to that labour. I think what we're observing around the anecdotes and concerns that are arising about people being able to find labour is at this stage much less an aggregate issue and much more a matching issue: are the people in the right place? How do they get there? Have

they got the right skills? This will sound strange, but I hope this problem persists. It's a good sign of a strong labour market. Wages are at record lows at the moment. We know we've got a really strong labour market when we see not just the anecdotes but people being prepared to pay people more and wages beginning to rise. We're on our way back, and we've got our first signs of the frictions we observe in a labour market. The government's policies should be aimed at facilitating to keep those frictions as low as possible. But I would say there is still substantial capacity on the labour side to be absorbed into the economy. Hence the importance of the recovery across the course of the next two years.

**Senator PATERSON:** More broadly on the recovery you've said, as have others, such as the RBA governor, that we are recovering much faster than expected, compared with budget and even MYEFO forecasts. What assumptions were made about the vaccine rollout in those forecasts? And how does that compare with what we now expect about the vaccine rollout?

**Dr Kennedy:** I might pass to Mr Yeaman on that. I believe they're largely consistent, but they may be just a little quicker, but I'll pass to Luke.

**Mr Yeaman:** Thanks, Secretary. That's correct. There was a document in the MYEFO—page 13 of part 2—which talked about our assumptions. It talked about it being assumed that there would be a COVID-19 vaccine available in Australia by March 2021, with a population-wide vaccination program fully in place by late 2021. That was to inform our broad forecast, obviously. It wasn't a precise estimate, but, as the secretary said, at this stage we would say that the progress on the health front is in line with our assumptions.

**Senator PATERSON:** Finally, on the issue of household balance sheets, there is this issue of what has previously been called a cliff when some measures come to an end. You've already pointed out that that's going to be naturally smoothed by the fact that payroll lags JobKeeper payments, so it's going to extend into April for some employers. Other things that will smooth that are the accumulation of savings on household balance sheets. Does that give you confidence that turning off JobKeeper as scheduled at the end of March is not going to open up some big hole in the economy that can't be filled by any other means?

**Dr Kennedy:** It does give me confidence. I will acknowledge that it will be a period of transition that we rarely see where we've increased a balance sheet very dramatically. I think household savings for this year must be up about 18-odd per cent. So, in the period ahead, household income will actually fall and we expect consumption to rise. It's not often we forecast that, but—

**Senator PATERSON:** Because household income has actually risen in this—

**Dr Kennedy:** It has risen very dramatically through this period because of the—

**Senator PATERSON:** Particularly for the lowest-income households.

**Dr Kennedy:** Yes. That's where it was targeted to have this impact on consumption. The other aspect that makes us a little more confident, and Luke mentioned this earlier when he was talking about consumption in the previous year, is that the housing market has held up very well. House prices are rising in some locations, particularly in regional Australia, so there is the wealth effect of people's confidence and, lastly, the confidence indicators are there. A good, clean read would suggest consumption will hold up strongly but, of course, we'll watch it closely because, as I mentioned to the chair, we don't get to forecast recovery from pandemics very often.

**Senator PATERSON:** Finally, because I know that time is limited, how unusual is it in a recession for household income to increase, even with all the assistance measures that government has put in place, and to do so to the magnitude that it has in this crisis?

**Dr Kennedy:** It's very unusual for it to increase in the magnitude that it has. It certainly didn't in the eighties and the nineties. Frankly, I would have to go back and have a look at the GFC for you, because there were—

**CHAIR:** It did go up, but not to the degree—

**Dr Kennedy:** There were packages, one in the order of $8 billion and a second in the order of $12 billion, in that response, in October and February through that period. As the chair has said, it was not to this degree.

**Senator PATERSON:** That really goes to the magnitude of the package.

**Dr Kennedy:** It does, yes.

**CHAIR:** On wages, are you in broad agreement with the RBA on that—that wages will remain flat until 2024?

**Dr Kennedy:** You've got the document there, but I think they increase back towards—

**CHAIR:** Yes.

**Dr Kennedy:** But it takes some time and, yes, our MYEFO forecasts have wages—Luke, what were they in the final year?

**Mr Yeaman:** We have the wage price index moving back to two per cent in 2022-23 and 2¼ per cent in 2023-24. But, broadly speaking, yes, we think that it's going to be some time before we see broad-based wage pressures in the economy.

**CHAIR:** Do you think that one per cent wage growth is a possibility in the interim? I think that's what the RBA is saying?

**Mr Yeaman:** Our MYEFO forecasts were around 1¼ per cent in the average sense. We're at those low levels now; that has been observed. We'll update our numbers for budget.

**CHAIR:** You don't have any updated numbers for me today?

**Mr Yeaman:** No.

**Senator SIEWERT:** Dr Kennedy, I want to go back to the issue of how many people you are now projecting will move from JobKeeper to JobSeeker once we get to 26 March and JobKeeper finishes.

**Dr Kennedy:** We think that, in the March quarter—and this underpinned the MYEFO estimates—JobKeeper would cover about 1.3 million people. That's what underpins the costing. Just to be clear at the outset, we don't think anything like that number of people would come off JobKeeper and go to JobSeeker. We actually don't know yet exactly how many people will be on JobKeeper in the March quarter because those numbers are just coming through, so it would be pretty hard for me to put a fine estimate on it, and it will depend a lot on whether there's no more widespread shutdowns in activity—effectively, that in the time ahead there are controlled outbreaks, not uncontrolled outbreaks. So I don't have a final number for you now, but I would expect the unemployment rate, rather than to be falling through that period, possibly to remain around stable—so a netting off of employment, with people perhaps coming in and perhaps getting jobs. As to what the actual flows will be—will that be 200,000 off and 200,000 on?—I'll have a better crack for you in our March meeting, because I'll have a much better idea about what the March quarter looks like. But that would be my broad guidance at this stage. I don't expect it to be many hundreds of thousands, by any stretch of the imagination.

**Senator SIEWERT:** So do you think that employers will keep the majority of them on? Is that what you're considering will happen at the moment?

**Dr Kennedy:** Yes.

**Senator SIEWERT:** Including those who are on the lower tier? In terms of those on JobKeeper now, how many are on the lower tier of payments?

**Ms Wilkinson:** A little over 10 per cent are on the lower tier.

**Senator SIEWERT:** Previously you've said that you expect that some of those people on the lower tier will also be receiving JobSeeker. Do you know if that's correct? Is that the case?

**Ms Wilkinson:** Certainly, if they meet the income test associated with JobSeeker then, yes, you can be on the lower tier and get JobSeeker. It depends on whether you meet all of the various conditions, including things like the partner income test, obviously.

**Senator SIEWERT:** Yes, obviously. Do you know how many that is?

**Ms Wilkinson:** I don't have that figure for you. I'm happy to take that on notice and see if we can get it for you.

**Senator SIEWERT:** Could you take that on notice. That would be appreciated. We were talking about those industries that haven't been doing well during COVID—aviation, for example, and service based industries. How many of the people that are currently on JobKeeper are in those industries that have not bounced back yet and are still more impacted by COVID and haven't been able to recover, for obvious reasons?

**Dr Kennedy:** There are two aspects to that. I'll ask Jenny to talk to the industry composition. One thing to also just hold in your mind is: Victoria is overrepresented in the December JobKeeper numbers, and that will flow through to March because of the late effect of that shutdown. Just to condition the industry effect: assuming things play out as we expect, that Victorian effect should taper out in the same way it did for the other states. What would Victoria make up—40-odd per cent?

**Ms Wilkinson:** In the second phase of JobKeeper, yes, Victoria has made up just under 40 per cent of total JobKeeper payments, up from under 30 per cent in the first phase.

**Dr Kennedy:** So inside JobKeeper at the moment is a particular kind of outbreak effect, and then there's the industry effect. I'll just pass to Ms Wilkinson on that.

**Ms Wilkinson:** The data that we have thus far is the industry data associated with the December quarter JobKeeper payments, and I guess it's worth recalling that eligibility for JobKeeper in the December quarter depended on your turnover test for the September quarter. That is why Dr Kennedy is absolutely right—when you look at these data, you see the impact of the Victorian shutdown and you see the impact of some of the ongoing restrictions, across all states and territories, including WA, that were still in place at that time. So, for example, the highest representation business in JobKeeper by an industry was actually construction at that point and the second-highest was professional scientific and technical services, and then there's a distribution across a range of different industries. You're absolutely right as to some of the industries that Dr Kennedy has talked about, and those like transport, postal and warehousing, which is where you would find the aviation sector. That's well represented. That's about the third-highest category. Accommodation and food services is about the 10th-highest industry. But perhaps, if it's useful, we could give you a table by industry on notice.

**CHAIR:** I think that would be useful.

**Senator SIEWERT:** That would be useful, because people on JobKeeper in those industries that have been hardest hit are potentially less likely to be finding work when JobKeeper finishes.

**Ms Wilkinson:** That's right. It's just that these are backward-looking data, so these were the industries that were hardest hit at that time, and of course the industry breakdown doesn't correlate directly with the employee breakdown, because there are some industries that have a higher proportion of smaller businesses and other industries that have a higher proportion of larger businesses. So it's a complicated picture, but we can certainly provide that industry breakdown.

**Dr Kennedy:** We are looking at that intersection with young people particularly, because, as I said, young people's employment hasn't recovered to the same extent that older people's employment has. We're drilling into it, but our expectation is that it's related to the impact, for example, on accommodation and cafes and those types of things, where many young people get first jobs or those types of jobs. Retail as a sector more broadly has actually increased its employment, and it also often employs people in their first jobs. So the story is quite mixed, but I guess the age employment data would suggest to you that there's probably an economy-wide age effect and then—and this is what you're getting at—an industry composition effect that is turning up in the industries where young people have jobs—service sector jobs and other 'first job on the job ladder' type jobs.

**CHAIR:** And the regional effect too?

**Dr Kennedy:** The regional one is really interesting. People talk a lot, for example, about the Cairns region or other regions that have high international tourism. We observe not as much regional variation as you might think. In fact, some people—and this perhaps goes to Senator Paterson's point—from an anecdotal point are reporting incredibly strong growth in some regions and difficulty in getting people to fill jobs. My mind continually turns back to the CBDs, frankly, in the cities, because they're quite different to outer parts of the cities. The regional picture is not at all like a standard recession. It's quite dispersed, and my sense, Senator Siewert, is that, in the recovery this time, it's probably best to focus less on region and more on looking very closely at affected sectors and, as you're arguing, the likely flow-on to those affected sectors. But my sense is that over time that's becoming narrower and narrower.

**Senator SIEWERT:** Can I go to the issue around older workers? I've heard what you said about overseas buyers. Have you broken that down by age cohort? For over-50s, there was an increase in the past in long-term unemployment. So what's it been like for the over-50s?

**Dr Kennedy:** I think 55-plus has had the largest increase.

**Ms Wilkinson:** They have, compared with pre-COVID levels.

**Dr Kennedy:** Compared to pre-COVID levels. But we're happy to provide that data on notice as well. It's obviously in the labour market data as it stands. I can pass to Mr Yeaman.

**Mr Yeaman:** Just to confirm that point, according to the ABS data that we monitor, the 55-plus age category now actually has a higher level of employment than before the crisis, by about two to 2½ per cent.

**Dr Kennedy:** What's making it so different to past recessions, where we worry about scarring on the old and the young—the young not getting into the labour market and the old being tipped out of jobs in industries that are rapidly transforming—is that we're not seeing the latter effect in the data at this stage.

**CHAIR:** Senator Siewert, I might just check in. We only have a couple of minutes left with Dr Kennedy. I might check in with Senator Lambie. I know she was multitasking. Senator Lambie, do you have any questions you'd like to put directly to Dr Kennedy before he leaves? If so, now is your chance. I don't think she's there. Sorry, Senator Siewert. You go.

**Senator SIEWERT:** Thank you. Dr Kennedy, I have a bollock-load of questions but I particularly want to ask you this question: has Treasury been doing any modelling around a permanent increase to the JobSeeker payment?

**Dr Kennedy:** Not for macroeconomic impulse effect, but certainly, along with the finance department and the relevant department, we're involved in all the conversations which look at the fiscal impact and otherwise of any changes to JobSeeker.

**Senator SIEWERT:** When did you do that modelling?

**Dr Kennedy:** As the government has foreshadowed, it's considering the end of the COVID supplement now and so we're advising them, along with other departments, on those arrangements.

**Senator SIEWERT:** Can I ask if you've been modelling for an increase in the JobSeeker payment?

**Dr Kennedy:** You can certainly ask!

**Senator SIEWERT:** Well, I'm asking then!

**Dr Kennedy:** It's a process which is right in front of government in their current considerations. So in general terms we're looking at a range of options—obviously.

**Senator SIEWERT:** Have you provided advice on a permanent increase?

**Dr Kennedy:** We're certainly involved in their considerations around advice on ongoing increases, but the government, as I said, will make their decision on the end of the COVID supplement and any subsequent changes. But we certainly advise them on all those matters, not only in the most recent period but in the past as well.

**Senator SIEWERT:** Okay. In the most recent period have you provided advice on the further rollout of a supplement to that payment, as opposed to a permanent increase?

**Dr Kennedy:** Senator, all I would say is that we're providing them with advice around all the options that go around the COVID supplement and then any further considerations. So all those issues are under consideration by the government. We're involved in ERC—the Expenditure Review Committee of cabinet—and we provide advice on all those issues.

**CHAIR:** Senator Siewert, we're almost out of time and I think you've gone as far as you could on that. Stay tuned is my recommendation. Just before I let you go, Dr Kennedy: business investment had been tracking downwards for many quarters before coronavirus hit our shores. What do you need to see in terms of an uptick in business investment to smooth or remove all the fiscal support as the private sector takes over?

**Dr Kennedy:** The form of business investment that we've been watching is non-mining business investment.

**CHAIR:** Yes.

**Dr Kennedy:** I will just ask Mr Yeaman to talk about what we have in our forecasts. In broad terms—

**CHAIR:** Yes, I have the forecasts.

**Dr Kennedy:** Well, if you see what we have in our forecasts, I'd be pretty confident that the investment recovery has begun. It is the case that around 2008-09, I think—or 2010—the level of non-mining business investment as a proportion of overall activity fell and hasn't really recovered.

**CHAIR:** That's right.

**Dr Kennedy:** It's actually a global phenomenon. As to what structural issues are re-emerging in the economy post COVID, that's a lot harder to predict. But in terms of what I was talking about before—feeling confident about the recovery locking in—I would be comfortable in seeing those forecasts being realised. That would give me confidence that business investment is tracking back up to a level that we'd hope to see and, hopefully, will go beyond that.

**CHAIR:** Okay. Again, we'll wait for any updated forecasts as part of the budget—

**Dr Kennedy:** You'll get them at budget in May, yes.

**CHAIR:** Yes, no worries. Dr Kennedy, your time is up. Thank you for your attendance today; we do appreciate it. It really greatly assists the committee with its work. I also thank other Treasury officials for remaining for another session with us at 12:15.

**Dr Kennedy:** Yes, thank you.

**CHAIR:** There's nothing else that you want to add, Dr Kennedy?

**Dr Kennedy:** No thank you, Chair.

**Proceedings suspended from 11:59 to 12:16**

**FISH, Ms Kirsten, Acting Second Commissioner, Law Design and Practice, Australian Taxation Office**

**HIRSCHHORN, Mr Jeremy, Second Commissioner, Client Engagement Group, Australian Taxation Office**

**O'HALLORAN, Mr James, Deputy Commissioner, Economic Stimulus Branch, Australian Taxation Office [by video link]**

**POWER, Mr Trevor, First Assistant Secretary, Macroeconomic Conditions Division, Treasury**

**PRESTON, Mr Robb, Assistant Secretary, Retirement Income Policy Division, Treasury**

**WILKINSON, Ms Jennifer, Deputy Secretary, Fiscal Group, Treasury**

**YEAMAN, Mr Luke, Deputy Secretary, Macroeconomic Group, Treasury**

**CHAIR:** Welcome. Would you like to make any opening comments or remarks?

**Mr Hirschhorn:** No; we have no opening statement today.

**CHAIR:** Can I ask some questions around JobKeeper and some of the data you're collecting on that program. Can you tell me what data you are collecting that you can share with the committee. Are you collecting information around firm size, numbers of employees and things like that—I presume you are, because you're reporting to Treasury—and are you looking at things like profits or increasing revenue?

**Mr Hirschhorn:** JobKeeper 1 was based on estimates of turnover effects. JobKeeper 2 is much more closely based on actual turnover, as per people's lodged activity statements. So we do monitor that.

**CHAIR:** When is that—for the December quarter?

**Mr Hirschhorn:** Yes. It is JobKeeper phase 2, which is from October, November, December. Even in JobKeeper 1 we monitored what people lodged with us as turnover, and we pay attention as well in situations where the disclosed turnover is very different from the estimated effect on turnover. Profits are trickier for us to monitor. We have the ability to monitor taxable income, which is almost like a sense of a tax law version of profit.

**CHAIR:** So you are monitoring that?

**Mr Hirschhorn:** We monitor that, but that is a very lag indicator because in the corporate sphere people lodge their tax returns six or seven months after the end of the year. So we will be expecting 30 June 2020 tax returns to come in now, and 30 June 2021 tax returns to come in a year after that.

**CHAIR:** Can you tell me anything, in what you're seeing from JobKeeper 2, in terms of the number of firms who are claiming JobKeeper but whose turnover has increased? Is there any information on that?

**Mr Hirschhorn:** For JobKeeper 2, you still have the condition in the JobKeeper scheme of your turnover having reduced by a percentage from the comparable period a year before. So, to get in, you have to have your turnover down. Of course, we do monitor to see if actual turnover—

**CHAIR:** Yes, I guess that's my question. So, they're in the scheme, and then how do you—

**Mr Hirschhorn:** By definition, if they're in the scheme, their turnover is down, and we check that. Maybe another way of looking at it is that in phase 1 of JobKeeper we had about a million businesses claiming JobKeeper, with about 3.8 million supported jobs. In phase 2 of JobKeeper we're down to about 500,000 supported businesses, with 1.6 million jobs. So, you can see that a lot of businesses that were eligible in JobKeeper 1 no longer meet the tests and are not claiming in JobKeeper 2.

**Ms Wilkinson:** Perhaps I could just add that in JobKeeper 2 the turnover test has to be applied for each quarter, so to get it in the December quarter—

**CHAIR:** Yes, so you could get booted off.

**Ms Wilkinson:** Yes, exactly. So, if you were eligible in the December quarter but your turnover in the December quarter didn't fall by 30 per cent, then you won't be eligible in the March quarter. Your eligibility depends on your turnover in the previous quarter.

**CHAIR:** Yes. So, you may get it for that quarter where your turnover didn't actually decrease—

**Ms Wilkinson:** You get it in that quarter because your turnover decreased by more than 30 per cent in the previous quarter.

**CHAIR:** Okay. So, in JobKeeper 1—do you have any information on that? Of those one million businesses, how many got JobKeeper but increased turnover at the same time?

**Mr Hirschhorn:** I don't have those figures with me. I'd have to take on notice what we could produce. I know that we do try to monitor—again, in JobKeeper 1, it was based on a reasonable estimate of what you expected to happen to your turnover—

**CHAIR:** But then did you have a look at what actually happened?

**Mr Hirschhorn:** I don't think we've done a population-wide analysis of that. We have done more of a compliance thing where it's one of our risk factors, where people's turnover differs greatly from their estimates. But I might pass to my colleague, Mr O'Halloran, who might have more detail.

**Mr O'Halloran:** Just to follow on from Mr Hirschhorn's comments, we have not done a population-wide analysis based on JobKeeper 1, if I could use that term. But certainly in the early stages and most of the way through JK1, where we did identify through various parameters, we did check, but probably more on a sampling basis, particularly larger corporates that had made their best endeavours or their estimates. I just don't have the number of instances that we've done that. But in checking the projection of the turnover that was made under the JobKeeper 1 rules, by and large we did find that they had a reasonable premise for the estimate they made. There were a small number of cases—I apologise; I don't have the numbers with me—where we did find that the estimate wasn't carried out. There might have been some other information that we felt would have influenced that judgement. But by and large we found that in the early stages of JK1 and throughout JK1 the estimates, particularly by the larger firms, were fair and reasonable and appropriate, given the conditions at the time, but when they had to make that judgement as to whether they had a reduction in turnover—

**CHAIR:** Perhaps you could take that on notice, and provide the committee with the information you have about that sampling.

**Mr O'Halloran:** Sure.

**Ms Wilkinson:** And one other thing I would add, just in thinking about this, is that of course because in JobKeeper 1 your eligibility was determined by your estimate that your turnover would fall, there are some businesses who legitimately may well have estimated that their turnover would fall by the requisite amount, and then, because they were eligible for JobKeeper, it enabled them to keep their business open, and then their turnover didn't fall by that amount. So there are quite a lot of things that are complicated, and that's exactly why the ATO took a very bespoke approach to trying to use those data to identify where there were some anomalies. But that's the other thing you have to keep in mind in this sort of analysis.

**CHAIR:** Is it the view under JobKeeper 1 that, because it was, by best endeavour, the best estimate of turnover and impact from COVID for those businesses that took JobKeeper during that time, there wouldn't be any recovery of funds? If their business was going really well and their turnover and profits increased, they still received this money; there was no legal requirement or other process to recover the money.

**Ms Wilkinson:** That's right, because, from a legal perspective, your eligibility for that payment was based on a reasonable estimate that you thought you would meet the turnover test. That's what provided you with the eligibility for the payment. There was nothing in the legislation that said ex post there'd be an assessment as to whether your turnover did or didn't fall or anything like that. Of course, that was partly because we were trying to deal with such an incredibly uncertain situation.

**CHAIR:** There isn't any work being done to look at whether it is worth going back and auditing and recovering some of that money, particularly for companies that have done extraordinarily well?

**Ms Wilkinson:** The ATO should speak about their compliance program. The compliance program reasonably focuses on where there was behaviour by businesses that was trying to manipulate the system. That's a separate issue. There's no suggestion that the scheme will be redesigned such that ex post, if outcomes turn out better, businesses will have to repay. But the compliance program absolutely focused on where there did appear to be anomalies in those early estimates.

**Mr Hirschhorn:** I would confirm that. Our interest is whether people complied with the rules that were in place. For that, our compliance task is: was it reasonable?—particularly in JobKeeper 1, which was based on an estimate and once you were in you remained in for the rest of JobKeeper 1. Our concern was that people inappropriately got in, not whether, once they got in, their business rebounded. That, in a sense, is irrelevant to their entitlements in JobKeeper 1.

**CHAIR:** In terms of your compliance work, putting aside the issue of businesses doing very well, do you have more information for the committee about those that got in that probably shouldn't have gotten in and what you've done about them?

**Mr Hirschhorn:** I can give you a few high-level figures. I would clarify that, of course, these figures move every day. To give some context, JobKeeper phase 1 was almost exactly $70 billion.

**CHAIR:** That's up to the end of—

**Mr Hirschhorn:** That's up to the end of JobKeeper 1. That has finished. The first phase of the JobKeeper extension is around $12 billion to $13 billion, so we're a bit over $80 billion overall.

**CHAIR:** As at the end of—

**Mr Hirschhorn:** As of 1 February.

**CHAIR:** I think our latest question on notice had it at $80 billion. So it's about $83 billion.

**Mr Hirschhorn:** I think it's around $83 billion. Of that, in terms of completed reviews, we reviewed claims worth about $7.5 billion. They hit some of our risk flags. Of that—

**CHAIR:** Can you give me a couple of examples of those risk flags?

**Mr Hirschhorn:** It could be both employer-based risk flags and employee-based risk flags. It could be that the employees nominated didn't make sense, for whatever reason. On the employer side, it might be that they didn't have an ABN in place or did not appear to have an ABN in place; they did not appear to have lodged previously.

**CHAIR:** You didn't know them.

**Mr Hirschhorn:** We knew them, but they had not previously disclosed employees. So there's a whole range. I won't do it justice, but that's both sides. The beauty of the design of the program was that we designed it around those existing systems. The vast bulk of the reviews were pre-issuance reviews: $6.9 billion of the $7.5 billion were pre-issuance reviews, and $600 million were post-issuance reviews. We have currently around $900 million of reviews still in progress.

**CHAIR:** When you say 'pre-issuance reviews', that's before the money goes out the door?

**Mr Hirschhorn:** That's before the money goes out.

**CHAIR:** So how much of that didn't go out the door?

**Mr Hirschhorn:** Now maybe I can talk about the things we rejected.

**CHAIR:** Yes, I suppose.

**Mr Hirschhorn:** We rejected about $180 million of claims pre issuance. That also cascaded. Indeed, we stopped people claiming in future, so once people were off we stopped their future claims. We estimate that to be about another $650 million of claims that we stopped going forward.

**CHAIR:** That's under JobKeeper 1 or 2?

**Mr Hirschhorn:** This is combined.

**CHAIR:** It's both?

**Mr Hirschhorn:** This is both.

**CHAIR:** JK 1 and JK 2—is that how you refer to it?

**Mr Hirschhorn:** Yes, but I should refer to it as JobKeeper extension. Then, in terms of overpayments, we've identified about 0.4 per cent, so $340-odd million, of which we've clawed back $135 million already. We're still pursuing about another $150 million, and the balance—

**CHAIR:** On top of that $340 million or as part of—

**Mr Hirschhorn:** No, these are components of the $340 million. You'll recall that right at the start there was a lot of concern about honest mistakes, where people applied but they'd got the rules wrong, and we said we were not going to pursue repayment if they had passed that on to the employees. That comes to about $50 million of the $340 million where we've determined somebody made an honest mistake and it was not appropriate to claw back the JobKeeper.

**CHAIR:** Okay. There are a lot of numbers there. In terms of money that you've got back, have you got a total?

**Mr Hirschhorn:** The money that we've paid out and clawed back is about $135 million.

**CHAIR:** Of that $340 million?

**Mr Hirschhorn:** That's of the $340 million.

**CHAIR:** In overpayments?

**Mr Hirschhorn:** Yes.

**CHAIR:** Then the ones before, the pre-issuance, is that money that never left.

**Mr Hirschhorn:** It never went.

**CHAIR:** So you don't need to recover it—okay.

**Mr Hirschhorn:** Maybe I can also give an update on, let's say, our cases. Five matters have been accepted by the Serious Financial Crime Taskforce, with another two under consideration. There are two entities where there is an active court case in relation to false or misleading statements, with another 16 under consideration, and 43 cases where we have levied penalties between 25 and 75 per cent, with 14 under consideration.

To give a bit of context on that, what this proves is that the design features and how we designed it around the existing systems, in conjunction with our Treasury colleagues, have really held up very well. In the context of an $80 billion scheme, this is very positive. I will put the flip side: the level of compliance has been extraordinarily high. As for the level of noncompliance, obviously we keep an eye out for it—we are the tax office—but it is a very encouraging result.

**CHAIR:** How confident are you that you're capturing everything you need to capture in the way you're approaching it? Do you think people could still slip through?

**Mr Hirschhorn:** Under the first design, it was actually very hard for the classic fraudster type to even get in the front door, because you needed to have an ABN of an existing, active business and you needed to have tax file numbers for employees. So it was very, very hard for the fraudster to get through the front door. In terms of the noncompliance we're picking up—we'll never capture all noncompliance. I suspect there is some sloppiness that we don't pick up, and we can't pursue every case. But I'm pretty confident that the figures I've given you are a very good indication of the compliance of the scheme.

**CHAIR:** The $83 billion that you've paid out and the $6.9 billion that you quarantined pre issuance—that's not part of that?

**Mr Hirschhorn:** We quarantined the $6.9 billion pre issuance—

**CHAIR:** So it wouldn't form part of the $83 billion?

**Mr Hirschhorn:** Of that $6.9 billion, we held back $180 million. Roughly $6.7 billion of that was paid out.

**CHAIR:** So when you held it back, the vast majority were good to go?

**Mr Hirschhorn:** Right. In response to your previous question, that also gave us confidence. We held back the ones which looked riskiest and, on review, the vast bulk of those were eligible in full, some were eligible in part and very few were completely ineligible.

**CHAIR:** Going back to the issue of those businesses that have done very well through COVID and received JobKeeper, has any analysis been done of how many of these companies—particularly those with turnover over $100 million—have increased their profit or paid out good dividends or executive bonuses whilst they've been in receipt of JobKeeper?

**Mr Hirschhorn:** From the ATO side, the short answer is no. Profit is an accounting concept reported through the ASIC financial statements. We are concerned with the turnover effects. In a sense, we find out accounting profit and dividends paid when people lodge their income tax returns. I can understand your interest for us, in a sense, focusing on whether the people appropriately got money under the rules as they are. It would be a very complex task but not relevant to our task. We don't have the data yet.

**CHAIR:** Maybe it's one for Treasury, then? Has the government asked that you look at this?

**Ms Wilkinson:** As Mr Hirschhorn said, because we don't have the tax returns—I'm not the Revenue Group expert, but I don't think we yet have the tax returns for the 2019-20 year. That would be the point at which you could do an analysis which looked at some measure of profits relative to the businesses that receive JobKeeper. We haven't done that analysis. We've obviously been monitoring the reporting, particularly through the newspapers, of different businesses, and there's different information coming up through the reporting season, but we haven't done a formal analysis of looking through the million or so JobKeeper businesses to analyse that yet. No, we haven't.

**CHAIR:** Has the government asked you to?

**Ms Wilkinson:** I don't think so, but I will take that on notice.

**CHAIR:** It is the largest program, at $83 billion. You can understand why people, when they're faced with having to pay this back in a budget repair sense, see businesses doing well, executives getting bonuses, dividends being paid and the public providing them JobKeeper, have a bit of, I think, legitimate concern about whether it's not a legal responsibility to return it but more of a moral responsibility now we're through the crisis.

**Ms Wilkinson:** At the end of January, the Treasurer certainly said, when he was asked, that, if businesses didn't need the JobKeeper money and were in a position to repay it, then he'd welcome that. But there's certainly no legal requirement for them to do so.

**CHAIR:** And no work is being undertaken to look at more formally responding to what the Treasurer said?

**Ms Wilkinson:** Not in terms of retrospectively changing the design of the program, no.

**CHAIR:** Do either Treasury or the ATO know how many companies have returned JobKeeper?

**Mr Hirschhorn:** What I can say is that there are a large number of companies who claimed JobKeeper for particular periods and then didn't claim it in subsequent periods. We think a component of those are companies who decided, perhaps for the reasons that you've suggested, that they no longer needed it, so they wouldn't claim it anymore. We of course don't have great data on that because they don't lodge a form saying, 'We stopped claiming because we don't need it anymore,' but we've done some research, and it appears that there are a significant number of companies who just stopped claiming.

**CHAIR:** Do you know how many that is?

**Mr Hirschhorn:** I'd have to take that on notice, but we don't have good data on that.

**CHAIR:** But it reduced from one million to 500,000?

**Mr Hirschhorn:** I'm putting aside the one million to 500,000, which is really a law requirement. I'm saying, even within JobKeeper 1, because it's a 'once in, you're always in' rule, some firms stopped claiming—for example—

**CHAIR:** So they're within that one million?

**Mr Hirschhorn:** Within that one million, some stopped claiming.

**CHAIR:** You must be able to run a report on that, aren't you? You would have seen the kind of decline within JobKeeper 1.

**Mr Hirschhorn:** We should be able to get you some numbers on that.

**CHAIR:** Okay, thank you.

**Mr Hirschhorn:** Of course, that will include some who stopped claiming because they were worried. Maybe we nudged them, and they stopped claiming. But we can get you an indicative number there. In terms of companies who have actively returned it, it is not a large number.

**CHAIR:** Do you have the number?

**Mr Hirschhorn:** I can say that it's in the order of 10 companies who are in discussions with us, not all of whom have repaid.

**CHAIR:** Do you have a figure for the total value of payments returned?

**Mr Hirschhorn:** Let's say that, from the around 10 who are in discussions with us about returning, it's about $50 million. Again, not all of it has been returned as yet.

**CHAIR:** Can you tell me how much has been returned now?

**Mr Hirschhorn:** I'll see if I've got that number in my briefing materials.

**CHAIR:** Okay. If you could come back to me on that, that would be excellent. Senator Davey, thank you for being patient.

**Senator DAVEY:** Thank you very much to all of you for attending today. I also have some questions on the compliance issues, but I might not beat around the bush quite as much as Senator Gallagher! Let's just cut to the chase. We read at the end of January—it got quite a bit of media coverage—claims by shadow Treasurer Jim Chalmers that JobKeeper money was going to 'dead people' or to 'deceased, fictitious employees.' Firstly, is that true, or did your compliance regime of red flags manage to prevent that occurring?

**Mr Hirschhorn:** I might go back to that article.

**CHAIR:** It was reported, I think, in—

**Mr Hirschhorn:** It was an ABC article.

**CHAIR:** the ABC, yes.

**Mr Hirschhorn:** So that was off the back of an FOI request around our risk approach to JobKeeper. I think, unfortunately, the journalist misunderstood the document and confused the fact that we had risk controls around high-risk categories of employee, like dead people, with the fact that we actually had a problem around fictitious employees. The journalist, I think, misunderstood, and so, unfortunately, that started this discussion.

I can confirm to the committee that our systems are very—because, again, this whole scheme is based around tax file numbers of individuals, the tax office is very good at tracking who has died. So we get monthly feeds from births, marriages and deaths, and that progressively happens. We are pretty good at that. We did identify a small number. Again, you've got to consider 3.8 million jobs were supported so 3.8 million tax file numbers, we did identify in those applications some people who our systems flagged as dead or likely dead. We reviewed those. There are no fictitious employees, so people making up people, or somehow harvesting dead people and claiming JobKeeper. There are none of those in the scheme.

In the scheme, again, with 3.8 million people over the course of now, I think, nine months, some employees die. So some people die. I think over the course of the scheme, our numbers are about 1,200 people died during the course of the scheme, but they were employees, they were real employees and appropriately reimbursed for the periods when they were working. But I can just confirm that there were no fictitious employees and a very small number, on review, of people whose employee had died and they maybe didn't immediately update their payroll system. So maybe they claimed an extra month, which we have clawed back. But there were no fictitious employees.

**Senator DAVEY:** That is reassuring. There was also the claim that some may have been in jail—real people with a real tax file number but, while they were sitting in jail, a former employer might have been claiming for them. Have you got a comment on that claim?

**Mr Hirschhorn:** Yes, I do. We again got data. We don't routinely get this data from the states, but we did get this data. We identified that there were 80 people who had been at prison at some stage during the period who JobKeeper was claimed for, for some stage during the period. We are reviewing all of those. Currently, we have identified no bad JobKeeper claims and we still have nine reviews in progress.

**Senator DAVEY:** The actual FOI report that Dr Chalmers was referring to confirmed there were no rorts? We just think that maybe this was a misinterpretation of the FOI? That FOI confirmed that there were no rorts?

**Mr Hirschhorn:** I think the FOI did not have these sorts of numbers in it. It was an FOI of a document about our risk approach to JobKeeper, which clarified that we have risk filters which look at things like backdated employees, so people who had not previously been an employee before 12 March but were backdated. It has a category for dead people. It has a category for people in jail and also unmatched tax file numbers—so a range of factors. That report said yes, we have risk filters for that range of factors. In the context of JobKeeper, the aim was to get money out quickly. In the context of a JobKeeper claim, if there are over a certain threshold of those in a particular claim, we will hold back the money and do a review pre-issuance. If there is only one or two, our risk approach was to pay out the money and then chase up. So the FOI report was really saying we are conscious of these risks, we are managing our program around these risks. I think where the journalist may have misunderstood that document was to hypothesise that it was actually a problem rather than a managed problem.

**Senator DAVEY:** My understanding is your compliance program has been examined by the ANAO. What did they find?

**Mr Hirschhorn:** The ANAO has a program across the entire COVID response of government, over multiple phases. They did a quick phase 1 review of the six major stimulus measures under the administration of the tax office. They had a very positive finding. I might even have it here. They had no recommendations for us. They had observations for whole of government, but they had no recommendations for us. They said 'the ATO has been effective in managing risks related to the rapid implementation of response measures' and that we 'undertook appropriate planning to support the rapid implementation of the six economic response measures—predominantly using existing systems and processes to support governance, resourcing and consultation.' They also said:

> In implementing the six economic response measures, the ATO assessed, documented and communicated changes in its risk environment in an appropriate manner. The ATO's risk documentation evidences its priority of implementing the measures in a timely manner, while also managing fraud and other integrity risks on a progressive basis.

And:

> The ATO has established effective arrangements to monitor identified risks and associated risk mitigation strategies through the project management and governance structures established for the six economic response measures.

Can I say, I was very chuffed on behalf of the thousands of people within the tax office responsible for the real hard work and nitty-gritty of developing these measures. I was very chuffed on their behalf. They put in a great effort, and I think the Australian community should get great confidence out of those findings.

**Senator DAVEY:** Well, certainly I'm very pleased to hear that we do have robust systems in place and proactive measures to identify the red flags and to investigate them. I just want to quickly move to the issue of businesses that qualify and the fact that JobKeeper is based on the preceding quarter. Does that mean that if a

business qualifies but then in the next quarter their turnover recovers they no longer qualify? But, as we've just seen, since New Year, in border communities in particular, some of those businesses who we could say had graduated out of JobKeeper then got impacted by border closures. They've seen their turnover go through the floor again. Can they reapply? Can they come back into the system?

**Mr Hirschhorn:** Yes. Each part of JobKeeper—JobKeeper 1, the second phase, the second quarter and now this third quarter, which is just starting—you are able to participate quarter by quarter. Say you were eligible for JobKeeper 1 and you weren't eligible for JobKeeper 2. You can become re-eligible for the last phase of JobKeeper.

**Senator DAVEY:** We are still in JobKeeper; JobKeeper ceases at the end of March. However, people can apply for this quarter, from January to March. So, while the program ceases, payments will be ongoing, because people can apply for this quarter. Is that right?

**Mr Hirschhorn:** That is right, although I might pass to my colleague, because there are in a sense deadlines that you have to apply for. I probably don't have at my fingertips whether it's still possible to apply. I would say that if you have constituent businesses that are in that position then it is probably worth their contacting us, rather than just lodging a form. But I might pass to my colleague.

**Ms Fish:** I would make just one clarification. It is correct that it's quarter by quarter under JobKeeper at the moment. So, we're in the last quarter for claims. Eligibility for claims in the current quarter, as Ms Wilkinson said earlier, is based on turnover having fallen in the three months to the end of December. So if you compare October, November, December 2020 with that same period in 2019, if you've had a fall of 30 per cent then you can claim for January, February and March this year. That's just one clarification. Entities that are enrolled in the scheme, enrolled in the program, can make claims. Generally you come in and make a claim in the first week after the month end. It is possible to come, as long as you're enrolled in the scheme, at a later point in time and claim for previous months. But, as Mr Hirschhorn said, it is preferable for those entities to come and talk to us because we can streamline that system; it might not be as easy to do it very much later online.

**Senator DAVEY:** I will process that information. I have some businesses along the border that are very interested in finding out whether they qualify. The key message is, though, that the ATO is there to help. If you've got questions, contact the ATO. You may be eligible, depending on your circumstances, so that is the key message.

**Senator PATERSON:** Just to be really clear, Mr Hirschhorn, sufficient information was clear from the original FOI that that was an erroneous report; it was a misinterpretation of the FOI. The information was adequate and on the public record at that time.

**Mr Hirschhorn:** I'm not sure how easy it was to access the FOI at that stage but it was clear. If I read some of it, in a sense, it said a risk factor for fictitious employees, 'Rule 6: AI identifies candidates where more than half of their employees have the following characteristics, which are not in themselves indicative of ineligibility; however, a cluster of them may indicate fraudulent or fictitious employees.' It talks about the characteristics. It talks about the relevant dates. The interpretation that there was a significant problem with fictitious employees—I'm not sure how that conclusion was reached.

**Senator PATERSON:** Given the material that was available?

**Mr Hirschhorn:** Given the material that was available.

**CHAIR:** In the media report?

**Mr Hirschhorn:** Given the material that was available in the FOI, the media report, we thought, had misrepresented what was in the FOI.

**Senator SIEWERT:** I go back to the JobSeeker payment, so I think this is for Treasury than the ATO. In MYEFO the government projected the impact on going back to $40 a day for the JobSeeker payment. Given the latest consumption figures and the impact the JobSeeker payment and now the stimulus measures, when you look over the whole of 2020, consumption still seems to be quite fragile. Have you got any new projections about the impact that reduction in the JobSeeker payment, if there's not another announcement made by government, will have on the projections that were contained in MYEFO?

**Mr Yeaman:** What I would say to the secretary's earlier comments is that the current profiles—the reduction that's currently planned and announced—as the government said, they are looking at this now. The reduction that is currently built into the forecast matches that. Our forecast will be presented at MYEFO, taking account of that existing policy step down. We still have built into our forecast, we think, a solid recovery in economic activity. I agree that since the release of the MYEFO consumption generally, along with other parts of the economy, are

subject to a lot of uncertainty currently. But, overall, since MYEFO, as the secretary said, we've been pleasantly surprised by the outcomes that we've seen particularly around retail trade, consumption and employment. We'll review it in the upcoming budget round of forecasts obviously but, at this stage, if anything, the economic recovery has, as we said, surprised us on the upside. At this stage, I wouldn't see an immediate reason to revisit those consumption judgements that were made in the MYEFO, but we'll keep it under review, clearly.

**Senator SIEWERT:** The fact is that JobKeeper will stop at the same time that JobSeeker goes back to the $40-a-day payment. Does it take into account the mortgage holidays that will be starting to come off at that time? Also, a lot of rent-in-arrears debt from all the rent deferments is now falling due, and that will particularly affect jobseekers.

**Mr Yeaman:** I agree there are a number of competing cross-currents, Senator. I agree with that completely. We've done our best to try and take account of those cross-currents in putting together our set of forecasts. I think the key point to remember from our perspective when we look at this issue, is that the underlying strength in the economy has dramatically improved with the easing of restrictions. As Dr Kennedy said earlier this morning, compared to a normal crisis, there is actually quite a significant step back in economic activity that flows automatically from the easing of restrictions. Then, on top of that, we're looking to see how we can stimulate those other areas of the economy that may still be subject to heightened uncertainty or ongoing restrictions as well. So we're seeing a step up in the economic activity underlying our forecasts.

The other point that Dr Kennedy touched on this morning that I'd come back to is while the direct effect of some of these support measures, as they're measured in the budget, is starting to step down, we know that a number of households have saved a large share of that income that has flowed to them. They've used the mortgage holidays that you've described, in some cases, to pay down their mortgage faster or to build up savings in an offset account. They weren't able to spend that money at the time, partly because of forced savings: they weren't able to go to the shops or they weren't able to go on the holiday that they wanted. There was uncertainty, so they were obviously being naturally cautious. Those factors are moving away now so we do think that presents a buffer—that even as the formal measures you describe start to come off, we still expect to see that income flowing back through the economy. I agree there are cross-currents here that we're trying to juggle but our forecasts try and take account of all of those things you've described.

**Senator SIEWERT:** First off, do you have an estimate or forecast on how many people, in particular jobseekers, are going to have rent debt given the rent deferrals?

**Mr Yeaman:** I haven't seen that, Senator. I don't know. I'll check with my colleagues behind me or if anyone else has that information. I haven't seen that combination of data put together. If it's available, we'll certainly look and we're happy to take it on notice.

**Senator SIEWERT:** Thank you. In terms of your projections, how can you be certain about the impact of the lifting of rent deferments and the requirement for rent debts to now be paid? How can you be certain that is not going to have a significant impact on individuals and on the broader issue of consumption?

**Mr Yeaman:** We're looking at the aggregate level, from the macro perspective, at what has been the overall macro fiscal impulse that has flowed from rent deferrals. So that's the amount of deferrals in aggregate across the economy that the banks and other lenders have provided, and what that has meant in terms of a fiscal impulse into people's pockets or in terms of relief. We can look at it from that perspective and there's no doubt that has been another material factor that's supported the recovery throughout. As that starts to wind down—it already has been winding down—that will have an impact. As I've said, we've tried to take account of that. That's the focus we have. I haven't looked at—and I'm not aware of work that's looked at—the specific vulnerabilities of those people in those cohorts. There may be others here or other departments that have, but we haven't looked at the specific cohorts. From a macro perspective, we're more interested in the aggregate impulse at the fiscal level.

**Senator SIEWERT:** Regarding the impact on job seekers specifically, we have an uncertain future at the moment, because we don't know what's happening to JobKeeper. It's supposed to end at the end of March. Job seekers are just about to get hit with debts—rent debts in particular. Has that been taken into consideration in the modelling and advice that you've given to government, in terms of the recommendations around the level of the JobSeeker payment?

**Ms Wilkinson:** I'm just not sure that we can go much further than where the secretary went this morning around the advice that we've provided to government as part of these deliberations. There's been a lot of advice that's been provided around a range of different aspects that go to this question about JobSeeker and any transition and any changes to the COVID supplement. But I don't think it's appropriate to speak about individual

components of that advice. As the secretary said, once the government's made their decision, then we can perhaps have a broader discussion around it.

**Senator SIEWERT:** How can you provide advice to government on the level of payment and the impact of the removal of the supplement—both at the macro-economic level and at the level of individual job seekers—if you don't have an idea of the quantum of the rent debt that is owed across the country? From what I can gather from your previous answers, we don't have an idea about what that level of debt is from the rent deferments that were put in place last year.

**Mr Yeaman:** To clarify, at the aggregate level—and I don't have the numbers in front of me on the spot—we have looked at what has been the overall impact of mortgage deferrals and rent deferrals and how much extra support has that provided into the economy, and, as that comes out of the economy, what do we think that's going to do to the macro outlook. So we are looking at those figures. If your question then goes to the individual level or the cohort level amongst job seekers—what pressure are they under and therefore what's a reasonable level of the JobSeeker supplement to come back to to manage those pressures?—as Ms Wilkinson said, a range of factors are being taken into account in the policy decisions that have been discussed, but those are still in train as we speak.

**Senator SIEWERT:** In terms of the availability of housing, one of the issues that's coming up in a number of places is the fact that there's very little availability and rents are going up. Has that also been taken into account in the projections for, in particular, housing affordability for job seekers?

**Ms Wilkinson:** Again, in any discussions around JobSeeker, there's a discussion around adequacy, and that takes into account a range of different factors that people on JobSeeker face. That includes consideration of housing costs and it includes consideration of things like Commonwealth rent assistance or the other assistance that some of those people may get through state government social housing programs. I'm not sure whether Mr Yeaman can speak in general terms on what we see happening with rents. Is there anything you can add there?

**Mr Yeaman:** In broad terms, as has been discussed in the press recently, we have more recently started to see some stronger house price growth, particularly in regional capitals—not so much in the major centres. I don't dispute that, certainly, there are people out there who would be struggling under rent stress. But, at the aggregate level that we monitor, we've seen that remaining relatively subdued, still, particularly in the apartments market.

We've seen relatively high vacancy rates and relatively weak rental price growth across the board at the macro level in the rental market, particularly in those inner-city suburb and apartment markets but that's not to dismiss the point that you made, Senator.

**Senator SIEWERT:** Good. You've just said that's an aggregate. Unfortunately, jobseekers can't afford those prices. Have you been looking at the housing which is actually affordable?

**Mr Yeaman:** As Ms Wilkinson said, there are a range of factors. We monitor from an aggregate level. In the policy discussion that's taking place, I think that those kinds of questions are being considered and addressed. But that's a process that's currently underway.

**Senator SIEWERT:** I want to go to the points that were made earlier by Dr Kennedy about the forecasts that have been done on the basis that the vaccines are being rolled out. If we see a problem with either the vaccine rollout or if we have a new strain that we don't have a vaccine for, have you taken that into account in your forecasts?

**Mr Yeaman:** As you said, Senator, forecasts assume that the vaccine rollout does proceed from around March through to the end of this year and that we have a nationwide vaccination program in place broadly by that time. If that weren't to occur—and there are obviously risks around that, although at the moment we think it's tracking largely according to our assumptions but it's obviously a fast-moving area—and there were to be changes to that rollout schedule, if there were to be concerns or problems with the vaccine rollout, that would definitely have an impact on our forecasts. But it's very difficult now to have a clear sense of how significant that would be, because it would depend very heavily on what the implications of that were.

For us, if we didn't have the vaccine rolling out quite as quickly as we had assumed but, in broad terms, borders remained open, we had activity remaining open and the current social-distancing measures were in place, then I think the economic recovery that we have forecast could still proceed largely as expected—potentially. That's obviously with some cost, but that would be mitigated to some extent.

If the vaccine rollout were linked to new variants, or if there were concerns were linked to new variants or other breakouts in Australia, that would have a bigger impact. But it's quite hard now to judge what the

implications of that would be going forward. It would definitely have an impact if the vaccine does not rollout as expected.

**Senator SIEWERT:** Thank you.

**Ms Wilkinson:** It's also the case that even once the vaccine is rolled out there is not an assumption that international borders would open overnight, for example. It's still the case that over the course of the next couple of years it will take some time for the full benefits of having a vaccinated population to flow through into the social-distancing measures, the decisions around borders and the decisions around migration.

**Mr Yeaman:** That's correct—particularly for international travel. We have not assumed that we have a fast return to international travel—tourism and business travellers—at the end of this year. It will take some time for that to build up over time.

**Senator SIEWERT:** Okay, thank you. I want to turn to the issue of consumption and retail spending. Have you looked at the impact across different regions and, if so, how much is the variance that you've identified for the potential impact on consumption across different regions in the country?

**Mr Yeaman:** In general?

**Senator SIEWERT:** Yes, in general.

**Mr Yeaman:** I might ask one of my colleagues, Mr Power, to come up and speak about that.

**Mr Power:** As Mr Yeaman said, at the high level we're really focusing on consumption at the total macro levels—so at the national level. We do look at how that is spread regionally in some of the cities. We look at where some of that spending is going and how industries are performing. We've talked about some sectors which are lagging. We talked a little bit, earlier this morning, about the regional composition of that. So we're aware of that and we're looking at some of those differences. But really, when we bring it up to the macro level, we're looking at that total level and how that will play out over the year. So I would say it's something that we are aware of and we do focus on. But, from a total Australian point of view, then it's something that we add up and look at what we think the trajectory will be over the year and going forward.

**Mr Yeaman:** If I can just add one point—I think the secretary alluded to this this morning. We have probably seen less regional variation in our economic statistics than we might have otherwise expected. So the consumption rebound in particular has been, I think, quite broad based across most areas. We haven't seen a large divergence across most of the sectors. We know there are sectors who are being affected mostly by the restrictions in other forms of activity. But, on consumption, it's been a fairly broad based pick-up overall.

**Senator SIEWERT:** That's in the last part of the year. What happens to those regional variations when JobSeeker payment is cut? Have you looked at that? I mean across the country. When I say 'regional', I mean not just in the regions; do you notice a difference across the states and in particular areas?

**Mr Yeaman:** Because of the cross-currents that I mentioned earlier, there are a lot of ons and offs in this system. JobSeeker is one element that is coming off. JobKeeper is there. We have the hiring credit coming on. We have business investment incentives coming on. We have those accumulated savings that I referred to earlier spread widely across the economy and also rolling out. So at this stage we're not anticipating that, as these measures roll on and off, we will see—in the way we look at the economy—localised areas of weakness or strength. Clearly, there are some areas that are still being affected by restrictions. We know, for example, that border communities, as I think was mentioned earlier by Senator Davey, are being affected as border restrictions come on and off. We know that there are still parts of the tourism sector that are being affected more heavily. But, because of those ons and offs, we're not expecting to see large regional differences from single measures.

**CHAIR:** Senator Siewert, a final question, and then we're moving on.

**Senator SIEWERT:** Okay. The chamber of commerce and industry has made some comments about different levels of JobSeeker payment, suggesting that people that are newly on it should get less, for example. Have you been asked to provide any advice about that proposal by the chamber?

**Mr Yeaman:** I'll just refer that to Ms Wilkinson.

**Ms Wilkinson:** As I say, we've provided advice around a range of different things to do with the JobSeeker payment. The thing I would just note is that it's already the case that people who are on JobSeeker and people in receipt of the COVID supplement receive a range of different payments depending on their eligibility for supplements and depending on their circumstances. So there exists a range. In terms of the broader discussions, there's a wide range of advice that we're providing that go to lots of different issues.

**CHAIR:** Thanks, Senator Siewert. If we have time at the end, I'll come back to you, because I know you never run out of questions!

Do you know how many Australians Treasury expects to be on JobSeeker by the end of the June quarter? Do you have those numbers?

**Ms Wilkinson:** I don't have that figure with me. The Department of Social Services will have that figure.

**CHAIR:** You don't—

**Ms Wilkinson:** I don't, no.

**CHAIR:** for purposes of your own?

**Ms Wilkinson:** I don't have that, no.

**CHAIR:** Perhaps this one will be for DSS too. When do you expect or when does Treasury expect the number of Australians on JobSeeker to return to the pre-pandemic level—in the order of between 700,000 and 800,000, I think?

**Ms Wilkinson:** Again, that's a question for DSS. We have projections for the unemployment rate. That's what we're responsible for.

**Mr Yeaman:** As per MYEFO, we have the unemployment rate coming back to around 5¾ per cent by 2022-23 and 5¼ per cent by 2023-24, and, as we've discussed, if anything, we think we're probably tracking ahead of those forecasts so far.

**CHAIR:** You'd obviously have the information when you provide it to government, in terms of what you're advising them on JobSeeker. But, for the purposes of today's hearing, that information is not available?

**Ms Wilkinson:** That's right. I just don't have it with me, sorry, Senator.

**CHAIR:** But Treasury would know that information?

**Ms Wilkinson:** In terms of the projections for JobSeeker, they are the responsibility of the Department of Social Services who get those costings through Finance—

**CHAIR:** But you would have them—

**Ms Wilkinson:** We contribute, particularly our projections—

**Mr Yeaman:** We provide advice on the economic and labour market projections which underpin that but there are a range of other administrative factors that feed into those numbers. There are administrative changes—

**CHAIR:** I'm just trying to confirm that it is information that you would like to know and would form part of your thinking as you're advising government. I went to the secretary on this. He didn't give a number. He talked in generality. But do you have a number people who are on JobKeeper and who will transfer to JobSeeker at the finalisation of that program? Do you have a number?

**Ms Wilkinson:** As the said secretary said, this is very hard to predict and there are lots of things which move in both directions and—

**CHAIR:** You haven't provided a number to government?

**Ms Wilkinson:** The information that's important for this consideration are things like: how many people who are on JobKeeper are actually working on zero or very low hours? That's quite important. We know that that's come down a long way. In the early phase of JobKeeper, my recollection, and I can confirm this, was of the order of 25 per cent of people on JobKeeper were on zero or very low hours. That's come down to, again I can confirm, around 10 per cent. Our best guess would be that between now and the end of March that will come down further, because with people on JobKeeper we are seeing two things happening. Some of them are transitioning to other firms and some of them are getting more hours with their existing employers. That's all moving in that direction. We're not expecting that there's going to be a very large stock of people who are on zero hours at the end of JobKeeper who will have to transition. Even if they do transition, some of them will transition into jobs rather than transitioning into unemployment.

**CHAIR:** Again, to be clear is your answer that you don't know how many? I'm not casting judgement on that. But is the real answer that you don't know or have you got scenarios where, under certain conditions, you have provided some of that analysis to government? I guess my worry is that government's ending programs without actually understanding the human impact. I know we talk in tens of thousands or hundreds of thousands—but people's jobs and livelihoods. Do you have a number or numbers under various scenarios? I don't think it's top secret—if you've got it. I'm not trying to go to what advice you've given to government about increasing the rate of JobSeeker. I'm trying to understand what you think will happen when one program finishes. We have got 1.6 million Australians on it or maybe less by that time. What happens?

**Ms Wilkinson:** The MYEFO forecasts are that there would be 1.3 million people on JobKeeper in the second half of JobKeeper 2.0. Of those we expect that there'd be a very small proportion who are still going to be on zero or low hours at the end of that. That could be of the order of 100,000 people, which was roughly the number of people at the end of JobKeeper 1, whose firms exited from JobKeeper 1, and who were on zero or low hours and who moved to other employment, so moved away from those firms. The challenging thing—

**CHAIR:** Moved away, like lost their jobs?

**Ms Wilkinson:** Some of them lost their jobs and some of them moved to other jobs. There are different components that we have estimates of. As the secretary said, this is rapidly evolving in terms of how the economy is continuing to evolve and how the labour market is continuing to evolve. What will matter at the end of March is how the adjustments take place within businesses and how the labour market adjusts, and that will depend partly on the broader state of the labour market at that time and how much further it has improved. We have certainly discussed all of these different components and factors with government, but there is not a number that is the number of people who are going to transition in one way or another.

**CHAIR:** Do you know how many firms are going to close or fold at the end of JobKeeper 2?

**Ms Wilkinson:** No, we don't.

**CHAIR:** Is it impossible to have a good level of knowledge about that?

**Ms Wilkinson:** I think that would be a very challenging thing to project. We don't typically make projections at the firm level. Of the 500,000 or so firms who are on JobKeeper 2 at the moment, about 40 or 45 per cent are sole traders and about 55 per cent are larger businesses. There are lots of different things that are happening within individual firms. We always get some firm turnover, but we don't typically provide projections in aggregate of the number of businesses that we're expecting to exit.

**CHAIR:** But we're in an unusual situation where we have at least 500,000 businesses who for the last 12 months have been relying on JobKeeper payments to keep going.

**Ms Wilkinson:** Yes.

**CHAIR:** And we've delayed insolvencies in certain situations, I imagine.

**Ms Wilkinson:** Yes.

**CHAIR:** But you don't have any understanding of what that might look like at the end of JobKeeper?

**Ms Wilkinson:** We don't have those estimates.

**Mr Yeaman:** We're looking as closely as we can at the overall strength. I would just come back to the point that the overall strength in the economy is there. We're seeing that recovery underway. Part of it is that it's also a moving target, because the data we're looking at around these firms, in terms of their turnover and some of their profitability, is often lagged till September and we're trying to make decisions around March. So it's quite difficult to build up a rich picture of how those individual businesses are going to be in March based on that dated data.

**CHAIR:** In terms of the next quarter, the January to March quarter, how many firms have submitted to you that they meet the decline-in-turnover test?

**Mr Hirschhorn:** I might defer a little bit to my colleague Mr O'Halloran, but my understanding of the timing is that it's sort of right now, because it's February, because it's a lag—

**CHAIR:** Yes, I think submissions to the ATO were due by the end of the January.

**Mr Hirschhorn:** I might pass to my colleague as to whether we have any early data. I'm not sure if we do, but I'll pass to my colleague.

**CHAIR:** Okay.

**Mr O'Halloran:** Chair, you're right: the claims or the declarations were due at the end of January. To be honest, I'm just looking for the figures now; we can certainly provide them on notice. They have been coming in at a reasonable rate given that there was a bit of a Christmas hiatus, for want of a better word. I apologise; I just haven't got the figure handy. I may find it before we finish. But certainly the rate is effectively the same as for the previous months. There's been a mixture of some new registrants—picking up the point made by the senator who spoke previously about people who have, if you like, found that they thought it was beneficial and appropriate and that they were eligible to come forward. So that pattern is there. I can't just pick up the figure while sitting here at the moment, but I'm happy to take it on notice and provide it to you, of course. But, by and large, it's projected to be a bit lower coming out of December and January, but certainly it is well progressed even though it is after 31 January.

**CHAIR:** If you have the figure, you will provide it for us?

**Mr O'Halloran:** Yes, I am happy to.

**CHAIR:** Do you know how many employees have been supported by JobKeeper in January, in the first two fortnights of this quarter?

**Mr O'Halloran:** The cumulative figure is still in the order of 1.5 million or 1.6 million, as mentioned earlier.

**CHAIR:** So there hasn't been much movement in this early stage from the December quarter to the March quarter?

**Mr O'Halloran:** No. With rounding, it might be closer to 1.65 million or 1.66 million or something. But it's of the same order; that's correct.

**Ms Wilkinson:** I think that's the month of December. I think we're a bit confused.

**Mr Hirschhorn:** Yes. Can we just take this whole question about, let's say, this JobKeeper 3 phase on notice, because the numbers rolling in right now, the numbers that I have in front of me, show that we're midway through analysing the numbers. We don't have a firm position on January yet.

**CHAIR:** So you have numbers in front of you that are—

**Mr Hirschhorn:** I have mature numbers in front of me for the months of October, November and December.

**CHAIR:** What do you have for January?

**Mr Hirschhorn:** I have incomplete numbers for January.

**CHAIR:** But you still have them?

**Mr Hirschhorn:** I have some numbers.

**CHAIR:** Can I have your incomplete numbers for January, then?

**Mr Hirschhorn:** Can I say these numbers are completely unreliable, because things are coming in.

**CHAIR:** Why have you got them? Why have they provided unreliable numbers to you in a brief for this hearing?

**Mr Hirschhorn:** They tell me what is the position at a date for the ones we've processed, but they are coming in.

**Ms Wilkinson:** I think it's more that they're incomplete. Our experience in recent months I think has been that about 90 per cent of the applications come in by about the 20th or 25th day of the subsequent month. So, we're only halfway there for the applications for the month of January. We're only halfway through, so we're getting the normal stream of applications come through.

**Mr Hirschhorn:** Senator, I suppose I can tell you these numbers, but they are not going to tell you how January is panning out. I think it would be much better for your purposes if you let us take it on notice and we gave you numbers in a week or two, when they mature for the month.

**CHAIR:** Okay. What I'm trying to get is whether you have an understanding in JK3 as to whether there's been any change between JK2 and JK3, and you're telling me that the data is not complete on that to give me an accurate picture.

**Mr Hirschhorn:** It's too early to tell—

**CHAIR:** I accept that.

**Mr Hirschhorn:** and in two weeks time we'll be able to give you a much better picture.

**CHAIR:** Okay. Can you also, then, give us the November and December figures as part of that?

**Mr Hirschhorn:** Yes, I can certainly give you the November and December figures. I can give you those now. In terms of supported individuals, in October we had 1.63 million, in November we had 1.6 million and in December we had 1.54 million.

**CHAIR:** And they are complete?

**Mr Hirschhorn:** Well, things will be dribbling in, but they're pretty much complete.

**CHAIR:** And the firms are in the order of 500,000?

**Mr Hirschhorn:** Yes. The firms for October were 512,000; November, 506,000; and December, 493,000.

**CHAIR:** Thank you. I've just got some questions on early-release super.

**Mr Hirschhorn:** Senator, before we go to that, you asked a question earlier about—

**CHAIR:** I might just need the stats.

**Mr Hirschhorn:** Given the small number, I don't want to be particularly precise, but I can say that we have received less than $10 million of repayments so far.

**CHAIR:** That was of the—

**Mr Hirschhorn:** Of the $50 million. I spoke about 10 entities, but about $50 million.

**CHAIR:** You've actually received $10 million?

**Mr Hirschhorn:** We have actually received $10 million.

**CHAIR:** Can you give me—I'm not necessarily up with the names, but is that from one or two or three? We had 10 companies.

**Mr Hirschhorn:** That's from a roughly proportionate percentage. I'd say it's about one-fifth.

**CHAIR:** So, two.

**Mr Hirschhorn:** Can I say that we're getting to very small, granular numbers.

**CHAIR:** I know. I understand. Alright. So, early-release super: Mr Hirschhorn, I don't know whether it's you or one of your colleagues. Can you just give the committee an update on that scheme? We asked you a question on notice about the breakdown of early-release data by postcode, and I think the answer came back saying that you're not in a position to do that; you did it by jurisdictional level, by state level.

**Mr Hirschhorn:** Yes.

**CHAIR:** Can I just follow that up and ask why you don't have that by postcode? I would have thought you would have had that information. Or is it because the funds have that?

**Mr Hirschhorn:** We have the tax file numbers of everybody who's claimed. It is a theoretically possible data-matching task. It is just a very complex and time-consuming one.

**CHAIR:** Is it? Okay.

**Mr Hirschhorn:** So we made that decision not to do it, because we didn't see the value in it.

**CHAIR:** I don't think you said that in the answer to the question on notice. It just said, 'We don't have that available.' So, it's not just a matter of searching your database by postcode?

**Mr Hirschhorn:** No. And I'm not going to profess to be a data analysis expert, but my understanding in probing the team is that this is actually quite complex, the way our databases work, to meld them together.

**CHAIR:** It's quite interesting information, though, one would have thought—to get a picture of where the applications for early-release super were coming from. Treasury wouldn't have it, would you?

**Ms Wilkinson:** We don't have it.

**CHAIR:** You'd rely on ATO, and the ATO's saying it's too onerous.

**Mr Hirschhorn:** It's possible, but we've made the decision that—

**CHAIR:** You haven't done it.

**Mr Hirschhorn:** it's too complex for the benefit.

**CHAIR:** Okay. Can you just go back and assure me that it is an onerous—

**Mr Hirschhorn:** I can absolutely assure you now—

**CHAIR:** I would like to take you at your word, but I would like to get the information that we're after.

**Mr Hirschhorn:** Yes.

**CHAIR:** Okay, if you could do that—thank you. On the final release super: do we have the final tallies of what's gone out in that program?

**Mr Hirschhorn:** Yes. We have what are effectively final tallies. I'll give you some numbers. These are what we approve, not what actually goes out of the super funds, but the two are correlated.

**CHAIR:** Yes.

**Mr Hirschhorn:** APRA gives data about what has come out of APRA related funds.

**CHAIR:** Out of the funds themselves, yes.

**Mr Hirschhorn:** As a rule of thumb, self-managed super funds seem to be about one per cent, so if you wanted to try to gross up their numbers—

**CHAIR:** Yes.

**Mr Hirschhorn:** We gave approval, or processed, about $20 billion of withdrawal requests in round 1 and $18 billion in round 2. About 950,000 people only claimed in round 1, about 600,000 people only claimed in round 2 and about 1.5 million people claimed in both. So in total, a little over three million people participated in the scheme.

**CHAIR:** Could we get a reconciliation of previous questions on notice where you've broken it down by age and gender? Could you update that?

**Mr Hirschhorn:** We can provide that. I think we are planning to do a summary report, which I think will be available relatively soon—not tomorrow and not next week—and which should have a lot of this data in it as well.

**CHAIR:** Has the ATO done that on its own initiative?

**Mr Hirschhorn:** The ATO is in the course of doing that.

**CHAIR:** What sort of a report is it? Is it just on details of the scheme—

**Mr Hirschhorn:** Yes.

**CHAIR:** Can you give me an update on any of the compliance issues? You had some issues around fraudulent applications; has there been any update on those?

**Mr Hirschhorn:** Yes. The lack of compliance that we were most concerned about was fraud, where people were taking other people's money. I think we've spoken previously about the fraud rate that we have detected, including the attempted fraud rate, being at about 0.05 per cent of applications. That ratio has remained the same; our estimate is that it's 0.05 per cent

We're also aware that in dealing with the funds, APRA has put forward a number of about 1,700 frauds or fraud attempts. That's about 0.04 per cent. We wouldn't expect those numbers to be exactly the same because some we head off before they go to the funds, but I think that gives pretty good confidence that it's around that level of fraud in the scheme, which is, again—

**CHAIR:** So around 1,700.

**Mr Hirschhorn:** APRA said 1,700—a smidge over 1,700. I think they published 1,703 recently.

**CHAIR:** And yours would align with that, roughly?

**Mr Hirschhorn:** Ours aligns roughly—ours is a little bigger.

**CHAIR:** Have you had to refer any matters to the police?

**Mr Hirschhorn:** Yes. We have referred matters. To give you a sense of it: we received about 8,000 of what are called 'suspicious matter reports' overall. We've received more, but some were duplicates. We looked into those and in about 6,700 of the 8,000 we found that there was no fraud, so they were false alarms. We referred 1,200 to the Serious Financial Crimes Taskforce and 100 are still under consideration. Of the 1,200 we have referred to the Serious Financial Crimes Taskforce, they have commenced six operations and that covers a significant percentage of the 1,200.

**CHAIR:** Because it was organised—

**Mr Hirschhorn:** An operation might cover a particular attack, which might have attacked multiple superannuation funds.

**CHAIR:** Okay. Is that all the information you can give me on that, presuming those matters are currently under—

**Mr Hirschhorn:** Yes, I'd prefer not to give much more on that.

**CHAIR:** But you're saying to me that there are six operations under investigation which cover a large proportion of the 1,200 referrals that you made?

**Mr Hirschhorn:** Yes. Of course, we have done other work around, in a sense, ordinary compliance—whether people were eligible—and the numbers have increased slightly since the last time we spoke. Based on ATO data—just based on data holdings that we have—we have high confidence that 94 per cent of the applications were eligible.

**CHAIR:** That is, there was some financial hardship?

**Mr Hirschhorn:** They met one of the tests. Through pilot work in some components of the population, our estimate is that, probably, overall compliance remains somewhere between 96 and 98 per cent.

**CHAIR:** In terms of people being eligible?

**Mr Hirschhorn:** Yes, people being eligible. Maybe the other thing I'd say is that, of those people in the pilot, we did about 2,000 reviews, which have resulted in 540 or so amendments to people's tax returns. The primary mechanism for, let's say, a relatively innocent breach is for us to make that amount assessable in their next tax return.

**CHAIR:** That's the penalty?

**Mr Hirschhorn:** The primary penalty is that the amount they withdrew is taxable.

**CHAIR:** That's about a quarter of the ones that you had a look at?

**Mr Hirschhorn:** Yes.

**CHAIR:** So the other ones were—

**Mr Hirschhorn:** Were fine. In a sense, if you did a back-of-the-envelope between the 92 and the 96 to 98, the 98 would say, when we've reviewed it, that about three-quarters were actually eligible.

**CHAIR:** I think the last time we met with Commissioner Jordan we raised the issue of financial abuse through the early release super. I think he said he wasn't fully aware of it but would have a look at it.

**Mr Hirschhorn:** Yes. We have had a look at it, and we spoke, I think, to Financial Counselling Australia as well. We had some informal discussions to get their ideas as to how we might identify it. It's fair to say, I think, that as an offensive behaviour it's hard to spot, but we did go through our tip-offs and complaints to try to find examples of potential financial abuse, and we found one example. Whether it was financial abuse or very poor spousal behaviour, we found one example. It was not where somebody was forced to take money out and had it stolen by their partner but where a spouse refused to release money to a former partner from their self-managed super fund. We looked—and absence of evidence is not evidence of absence—but we did not find any indication that there was significant financial abuse.

**CHAIR:** I appreciate that. Thank you for that update. This might form part of the report you're going to do, but how many applications were rejected?

**Mr Hirschhorn:** I've got some numbers there. In the phase 1 we received 2.54 million applications overall and we approved 2.45 million. So we rejected 90,000 applications in round 1.

**CHAIR:** Was the main reason for rejection not meeting the eligibility requirement?

**Mr Hirschhorn:** That was mainly self-assessed. It was probably more that people claimed a second time—you're only allowed to claim once—or other reasons.

**CHAIR:** Within that phase?

**Mr Hirschhorn:** Within that phase; you're only allowed to claim once per phase.

**CHAIR:** People were trying to get more out?

**Mr Hirschhorn:** Yes. In the second phase, we received 2.24 million applications and we approved 2.1 million. So that's 140,000 that we rejected.

**CHAIR:** For the same reason?

**Mr Hirschhorn:** I'd have to take on notice whether we have more detail on that, but I suspect it was mostly that.

**CHAIR:** Thank you very much, Mr Hirschhorn. I appreciate the numbers you've given me.

**Senator PATERSON:** I have just a quick follow-up question on the early release super scheme. I am not sure if you saw, Mr Hirschhorn, yesterday, former Prime Minister Paul Keating made some comments about the scheme? It was reported in the *Financial Review*. He said people were withdrawing their super to pay for 'a new Kia car or a new pair of skis or something else'. To your knowledge, how many Kia cars or skis have been purchased with money withdrawn from super under the early release scheme?

**Mr Hirschhorn:** I am aware of that article. It's fair to say we are concerned at the tax office with whether people are eligible to withdraw money from the scheme and processing it and, indeed, helping the super funds who have the obligation to release. We do not even attempt to track what people ultimately spend their money on. Once it is released, it is their money and no doubt Australians spend it wisely as they would in their ordinary life.

**Senator PATERSON:** So there is no data set on Kia sales or ski sales?

**Mr Hirschhorn:** Certainly the tax office would not collect that data.

**Senator PATERSON:** You're not aware of it? Treasury, are you aware of a data set on Kia—

**Mr Preston:** No, sorry. I'm not aware of any data that would be used. I'm not sure where Mr Keating got those claims from. We tend to refer to the ABS survey work done late last year, suggesting around 60 per cent of people nominated paying for household bills or groceries, mortgage, rent, and around 20 per cent were suggesting they would save the money. They are the figures we tend to look to in terms of where the money went.

**Senator PATERSON:** That seems like a more robust methodology to work out how people are spending their money than pure speculation based on no data.

**Senator SIEWERT:** I want to just go back to the early release super scheme. Do you know how many applied for super who were on the JobSeeker payment? Is that data that you have?

**Mr Hirschhorn:** I do not have that data in front of me. I'm not sure if we are able to find that data. In response to the Chair's previous question, we have produced and will produce again data on how many people on income bands but I'm not sure if we've identified, say, the particular people in a particular income band or on JobSeeker or not. Maybe this might be one which is better done in a table, in a question on notice, but I can tell you that in, for example, people earning less than $18,000, so between $1 and $18,200, there were about 200,000 applications in the second phase.

**Senator SIEWERT:** That's in the second phase?

**Mr Hirschhorn:** Yes.

**Senator SIEWERT:** Do we know for the first phase?

**Mr Hirschhorn:** I think I have tranche 2 data in front of me rather than total scheme data. I would have to come back to you with that number.

**Senator SIEWERT:** I would appreciate it if you could, thank you. I will go back to where I left off, asking about the types of payments. The question related to the Chamber of Commerce and Industry and the answer that Ms Wilkinson gave. First, you gave quite a cute answer in terms of the different levels of payment. The base rate doesn't vary, so I will be much more specific. Are you considering different base rates of a payment? Have you given advice on different base rates for the JobSeeker payment?

**Ms Wilkinson:** Again, as the secretary said earlier when you were asking for details of the advice that we've provided and the different things that governments are considering, it's just not appropriate for us to talk about the range of different things on a matter which is under consideration by government at the moment. I wasn't trying to be cute. It is genuinely the case, and for good reason, that under the JobSeeker program there are different payments that are available to different people in different circumstances. That is a feature of the current program. I think sometimes that's lost in the debate: different people on JobSeeker are actually earning different amounts. But I just don't think it's appropriate to talk about the different sorts of information or advice that are or aren't being provided at the moment.

**Senator SIEWERT:** Have you been asked to provide advice on different base rates?

**Ms Wilkinson:** No, we haven't been asked to provide advice on different base rates.

**Senator SIEWERT:** Have you been asked to provide advice on differences in payments for those who are long-term unemployed and those who have been on the payment for a shorter time?

**Ms Wilkinson:** Again, this is just another way of asking what advice has been provided.

**Senator SIEWERT:** I'm not asking for the advice. I'm asking: have you been asked to provide that advice?

**Ms Wilkinson:** I appreciate that. I think the challenge is that it goes to a question of what are the different options that may or may not be considered. I'm certainly aware of the ACCI proposal. I'm sure the government's aware of the ACCI proposal. There are a range of different considerations that feed into any discussion around adjustments to payment rates.

**Senator SIEWERT:** Have you been asked to provide advice on the Commonwealth rent assistance payment and the sufficiency of that?

**Ms Wilkinson:** Commonwealth rent assistance is clearly one of the payments associated with people who are on JobSeeker, so advice on Commonwealth rent assistance is usually provided as part and parcel of advice around any matters to do with JobSeeker.

**Senator SIEWERT:** Should I take that as a yes?

**Ms Wilkinson:** Again, we have been asked to provide advice through the normal expenditure review cabinet process on a range of matters to do with welfare payments.

**Senator SIEWERT:** Thank you. I will go back to the issue of the immediate effect on consumers that the removal of the JobSeeker payment will have, and I ask: have you looked at it electorate by electorate?

**Ms Wilkinson:** Again, I'll turn to my colleague Mr Yeaman, who is responsible for the consumption forecasts. I think the way in which we do our consumption forecasts is not a bottom-up electorate-by-electorate process where we then aggregate them into an aggregate consumption forecast. As Mr Yeaman outlined at the-outset, it's much more stepping back, looking at the range of different factors that influence consumption, including things like income, and taking all of those into account when you're coming up with your forecasts.

**Mr Yeaman:** I can confirm that. The answer is no.

**Senator SIEWERT:** Thank you. Have you looked at the impact not just on consumption but on the amount of money it takes out of the economy electorate by electorate?

**Mr Yeaman:** No.

**Senator SIEWERT:** Is that something that you have ever done?

**Mr Yeaman:** Not to my knowledge, no.

**Senator SIEWERT:** Have you seen the analysis that's been done that suggests that it takes out around $2 million per electorate per fortnight?

**Mr Yeaman:** I haven't. No, I'm not familiar with that analysis.

**Senator SIEWERT:** If it is correct, what impact would taking $2 million per fortnight out of electorate spending have on each of those electorates?

**Mr Yeaman:** Without having seen the analysis, it's very hard to comment. Clearly, electorates are not a category we look at closely. They all vary in size, geographic distribution and income, so it would be quite a complex exercise to work out what the impact of a net reduction of a certain amount would be to economic activity within each electorate. So the short answer is no. I'm not familiar with that analysis, so it's very hard to comment.

**Senator SIEWERT:** Thank you. You commented earlier about the levels of saving that people have at the moment. Are you aware of what the saving level is for those on JobSeeker?

**Mr Yeaman:** Not off the top of my head. As a general rule, my understanding is that savings rates for lower-income households would generally be lower than those for higher-income and middle-income households but I haven't seen recent data to confirm that. We can have a look at it for you if you like.

**CHAIR:** Senator Siewert, do you have a final question? You've had a pretty good trot.

**Senator SIEWERT:** It would be appreciated if you could take that on notice. As you've articulated, and from the data I have seen today, people are spending the increase in the JobSeeker payment. Some have probably saved a bit, but they have actually been meeting their needs in terms of spending it. Would that be a correct interpretation of what is happening at the moment?

**Mr Yeaman:** In the broad. I don't have the precise figures in front of me and I haven't looked at them in the immediate past. We can take it on notice and confirm it for you. My understanding is that, broadly, you are right: we do generally expect low-income households to spend relatively more of the income they receive than middle- and high-income households. But my recollection is that, even among low-income households, with the level of support that has come in, we are seeing some of that being saved and put towards mortgage payments and into offset accounts, which I think the Reserve Bank governor has talked about a number of times as well. So it is a combination: we are seeing some going into saving and we are seeing some go into current consumption. But we are happy to take that on notice.

**CHAIR:** I thank officers for their evidence to the committee today. We really appreciate it. We accept how busy everybody has been and continues to be. Your evidence significantly assist us with the work we've been asked to do by the Senate, so the committee does appreciate it. For any questions taken on notice, please provide answers by 25 February 2021 or seek an extension as some do.

**Committee adjourned at 13:57**
